# A Near Telomere‐to‐Telomere Genome of *Belamcanda chinensis* Provides Insights Into Genome Evolution and the Biosynthesis of Characteristic Isoflavones

**DOI:** 10.1111/pbi.70612

**Published:** 2026-03-14

**Authors:** Yuan‐Yuan Wang, Bi‐Huan Chen, Gui‐Sheng Xiang, Yi‐Na Wang, Run Yang, Xiao‐Bo Li, Shi‐Yan Yuan, Yu‐Cheng Zhao, Guang‐Hui Zhang, Min‐Jian Qin, Sheng‐Chao Yang

**Affiliations:** ^1^ College of Agronomy and Biotechnology, National‐Local Joint Engineering Research Center on Gemplasm Innovation & Utilization of Chinese Medicinal Materials in Southwest, the Key Laboratory of Medicinal Plant Biology of Yunnan Province, Yunnan Agricultural University Kunming Yunnan China; ^2^ Yunnan Characteristic Plant Extraction Laboratory Kunming Yunnan China; ^3^ Department of Resources Science of Traditional Chinese Medicines School of Traditional Chinese Pharmacy, and State Key Laboratory of Natural Medicines, China Pharmaceutical University Nanjing China; ^4^ Yunnan Province Key Laboratory of Cross‐Border Chinese Herbal Materials, Honghe University Mengzi Yunnan China

**Keywords:** *Belamcanda chinensis*, genome, glycosylation, isoflavones biosynthesis, methylation

## Abstract

*Belamcanda chinensis* is a non‐leguminous medicinal plant rich in bioactive isoflavones; however, the lack of a high‐quality reference genome has limited elucidation of its isoflavone biosynthetic and modification network. Here, we present the first near telomere‐to‐telomere genome assembly of *B. chinensis* (4.18 Gb), generated using Illumina survey reads, PacBio HiFi and Oxford Nanopore long reads, and Hi‐C scaffolding, achieving high completeness and accuracy (assembly BUSCO: 98.70%; LAI: 17.2). Ks/synteny‐depth analyses and fossil‐calibrated dating, with calibration at four fossil nodes, indicate two lineage‐specific WGD events (~54.6 and ~27.3 MYA). These events drove significant expansions of key gene families involved in stress response and secondary metabolism. Leveraging this genome, we identified two key O‐methyltransferases (BcOMT03 and BcOMT33), which are responsible for catalysing the biosynthesis of quality‐marker compound irisflorentin. Meanwhile, BcUGT009, BcUGT119, BcUGT124, and BcUGT032 were characterised as glycosyltransferases with 7‐O catalytic activity. Structural modelling and site‐directed mutagenesis further elucidated the catalytic mechanism of BcUGT009, and its K404A mutant exhibited a significant increase in relative activity. Cross‐species comparative analyses further revealed that convergent expansion of these key enzyme families underlies isoflavone biosynthetic capacity in both leguminous and non‐leguminous plants. This study not only reveals the ancient polyploidization events of *B. chinensis* , the amplification of lineage‐specific gene families and the biosynthetic pathway of characteristic isoflavones, but also provides a reference genome and functionally validated tailoring enzymes that will facilitate future heterologous pathway reconstruction and metabolic engineering of these isoflavones.

## 1 Introduction

**FIGURE 1:**
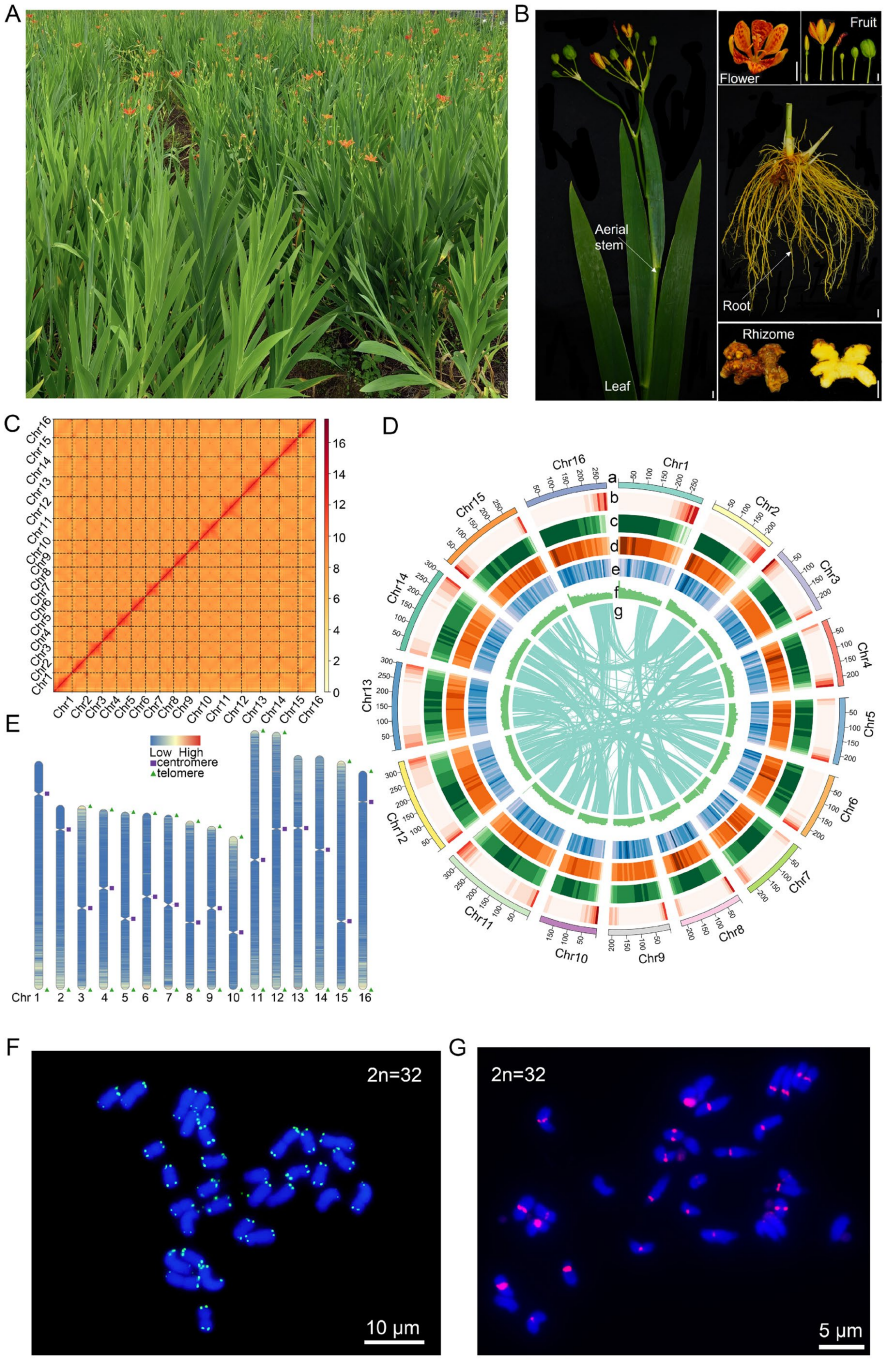
Morphology, near‐T2T genome assembly, and cytogenetic validation of *B. chinensis* . (A) Image of the whole plant. (B) Morphology of leaf, aerial stem, flower, fruit, root, and rhizome. Scale bars, 1 cm. (C) Hi‐C contact heatmap of chromosomal pseudomolecules, showing strong intra‐chromosomal interactions and high assembly continuity. (D) Genomic landscape of the 16 pseudochromosomes, displayed as linear tracks from outside to inside: (a) chromosome length; (b) gene density; (c) repeat sequence density; (d) *Gypsy* density of repetitive sequence; (e) *Copia* density of repetitive sequence; (f) GC content; (g) collinearity. (E) Distribution of centromeres (purple squares), telomeres (green triangles), and gene density (red, high; blue, low) across the genome. (F) Fluorescence in situ hybridization (FISH) of telomeric repeats (AACCCT)₃ on metaphase chromosomes; strong green signals localise to chromosome ends. Scale bars, 10 μm. (G) FISH localization of the centromere‐specific tandem repeat (red) on metaphase chromosomes. Scale bars, 5 μm. DNA counterstained with DAPI (blue).

The Iridaceae family, comprising 69 genera and over 2000 species (Plants of the World Online [POWO]) (POWO 2025), is renowned for its large, brightly coloured, and uniquely shaped flowers, which hold exceptional ornamental value. Beyond their ornamental appeal, many species are pharmacologically significant. *Belamcanda chinensis* (L.) DC is the sole species of the genus *Belamcanda* in Iridaceae and possesses both ornamental and medicinal value (Figure 1A,B). As a traditional herb in China, it has been widely used to clear away heat, eliminate phlegm, relieve sore throat, disperse stagnation, and diminish inflammation for thousands of years (Wang et al. 2024). Previous pharmacological studies demonstrated that the extract of *B. chinensis* rhizome has many biological effects, such as antiviral, anti‐tumour, antibacterial, and antithrombotic (Xin et al. 2015). At present, various preparations of *B. chinensis* rhizome have been listed and used in national drug standards such as the Pharmacopoeia of the People's Republic of China (National Pharmacopoeia Commission 2020).

The primary pharmacologically active components of *B. chinensis* are isoflavones (Zhang et al. 2016), which serve as important precursors for the biosynthesis of phytoalexin derived from the phenylpropanoid pathway (García‐Calderón et al. 2020; Sharma et al. 2024). In plants, isoflavones often undergo post‐modification reactions such as methylation and glycosylation, which enhance their chemical stability, bioavailability, and bioactivity (Li, et al. 2023), thereby expanding structural diversity and generating more complex secondary metabolites. In *B. chinensis* , O‐methylation and O‐glycosylation produce bioactive isoflavones such as irisflorentin, tectoridin, and tectorigenin. Irisflorentin, a quality control marker for *B. chinensis* in the Chinese Pharmacopoeia, exhibits significant potential in neuroprotection, anti‐inflammatory, and immunomodulatory (Patel 2023). Tectoridin and tectorigenin provide defence against various biotic stresses, including pathogens and pests (Cheng et al. 2023). Despite their importance, the biosynthesis of these modified isoflavones in *B. chinensis* remains poorly understood, particularly in non‐leguminous species.

While the biosynthetic pathways of isoflavones have been extensively characterised in leguminous plants, their presence and functional significance in non‐leguminous species, particularly within the Iridaceae family, have garnered increasing attention. In *B. chinensis* and related Iris species, isoflavones play crucial defensive and pharmacological roles, prompting recent efforts to elucidate their modification enzymes. For instance, a reversible *7‐O*‐glycosyltransferase (Bc7OUGT) was functionally characterised from *B. chinensis* , which interconverts tectorigenin and tectoridin, and its catalytic mechanism was revealed through structural analysis (Cheng et al. 2023). Similarly, UGT73CD1 from *Iris tectorum* Maxim. was identified as a promiscuous glycosyltransferase capable of *7‐O*‐glycosylating diverse flavonoids and isoflavones (Huang et al. 2021). Furthermore, Zhang et al. (2021) showed that multiple inducible glycosyltransferases (BcUGT1‐8) in *B. chinensis* primarily catalyse *7‐O‐* and some *3′‐O*‐glycosylation of isoflavones such as tectorigenin and irigenin. However, other characteristic isoflavones produced by *B. chinensis* , such as glycosyltransferases that catalyse the production of iristectorin B and iristectorin A, and methyltransferases that are responsible for modifying dichotomitin to produce irisflorentin, have not been determined.

Previous transcriptomic and metabolomic analyses of six organs in *B. chinensis* revealed spatial concordance between isoflavone accumulation and gene expression in underground tissues, providing a foundation for candidate gene selection (Tian et al. 2018). However, transcriptome data alone cannot resolve complete biosynthetic pathways or reveal genomic architecture. The lack of a high‐quality reference genome has hindered the systematic identification of gene families such as OMTs and UGTs, the reconstruction of biosynthetic gene clusters, and the understanding of how whole‐genome duplications and repetitive landscapes have shaped isoflavone diversification in *B. chinensis* .

Recent advances in sequencing technologies now enable telomere‐to‐telomere (T2T) genome assemblies, which offer unprecedented completeness and accuracy for studying complex genomic regions, including centromeres, telomeres, and repetitive element‐rich intervals. A near‐T2T genome is particularly crucial for *B. chinensis* , given its large genome size (~4.18 Gb) and high repetitive content, which complicate gene annotation and evolutionary analysis. Such a genome would not only elucidate the genomic basis of isoflavone biosynthesis but also provide insights into lineage‐specific adaptations shaped by polyploidy and transposon activity.

To address these problems, we generated a near‐T2T genome assembly of *B. chinensis* by integrating Survey, HiFi, Hi‐C, and ONT sequencing. Leveraging this resource, we performed genome‐wide identification and functional characterisation of O‐methyltransferases and UDP‐glycosyltransferases involved in isoflavone modification. This study provides a genomic foundation for understanding the evolution of specialised metabolism in a non‐leguminous medicinal plant and identifies enzyme tools for synthetic biology applications.

## 2 Results

### 2.1 A Near Telomere‐To‐Telomere Genome Assembly of *B. chinensis*

*B. chinensis* is a diploid species with a chromosome number of 2*n* = 2*x* = 32 (Figure S1). Genome size estimation based on kmerfreq (*k* = 17) and GCE indicated a haploid genome size of approximately 3.99 Gb, with a low heterozygosity rate of only 0.42% (Figure S2). Comprehensive statistics for genome sequencing and assembly are summarized in Table 1.

**TABLE 1:** *B. chinensis* genome sequencing and assembly.

Genome‐sequencing	Size	Depth (×)
Survey (Gb)	588.95	~124×
HiFi (Gb)	171.31	~40×
ONT (Gb)	97.20	~23×
Hi‐C (Gb)	1065.35	~225×
Estimated genome size (Gb)	3.99	
Estimated heterozygosity (%)	0.42	
Number of contigs	64	
Total length of contigs (bp)	4 191 429 687	
Average length of contigs (bp)	65 491 088	
Contigs N50 (bp)	209 613 489	
Longest contig (bp)	336 376 737	
Contigs N90 (bp)	66 287 037	
GC content (%)	40.18	
Total sequence length (bp)	4 178 654 823	

Hi‐C contact maps revealed strong intra‐chromosomal interaction signals and a near‐complete absence of abnormal inter‐chromosomal contacts, demonstrating high contiguity and structural fidelity of the chromosome‐scale assembly (Figure 1C). A 4.18‐Gb near‐T2T genome assembly was generated, comprising 16 chromosome‐scale pseudomolecules assembled from 64 contigs with a contig N50 of 209.6 Mb (Figure 1D; Tables S1–S3). Telomeric repeat arrays were identified at both ends of 12 pseudochromosomes, while the remaining four chromosomes retained a single telomere each; the number of telomeric repeat units per chromosome terminus ranged from 339 to 2861 (Figure 1E; Table S4). The genomic coordinates and lengths of all 16 centromeres were defined by integrating Hi‐C interaction profiles with computational predictions (Figure 1E; Table S5).

Fluorescence in situ hybridization (FISH) was performed to independently validate the structural integrity of the chromosome‐scale assembly. Signals from a telomere‐specific probe (AACCCT)_3_ were detected at chromosomal termini, and a centromere‐specific probe derived from the major tandem repeat localised precisely to the primary constrictions (Figure 1F,G), confirming the correct placement of both telomeric and centromeric regions in the assembly.

### 2.2 Genome Annotation and Evaluation

Approximately 90.22% of the *B. chinensis* near‐T2T genome was annotated as repetitive sequences, dominated by LTR retrotransposons (77.19%), particularly *Gypsy* (32.28%) over *Copia* (5.54%); DNA transposons account for 10.32% (Table S6). LTR insertion time analysis revealed a major amplification burst between 0 and 6 MYA, peaking within the last 1 MYA (Figure S3).

A total of 33 962 protein‐coding genes were annotated, with a mean transcript length of 1297.73 bp, an average of 5.3 exons per gene, a median CDS length of 1017 bp, 7244 mono‐exonic loci, and 96.4% of transcripts possessing a CDS/cDNA ratio ≥ 84% (Table S7). In addition, 6459 non‐coding RNAs were annotated, including 778 tRNAs, 4968 rRNAs, 478 snRNAs, and 235 miRNAs (Table S8). Functional annotation was achieved for 33 291 genes (98.02%), with strong support from multiple public databases: Nr (96.44%), KEGG (95.32%), TrEMBL (95.59%), InterProScan (96.27%), and GO (76.01%) (Table S9). The annotation BUSCO evaluation achieved a high completeness score of 97.5%, indicating that most gene annotations are credible (Table S10). A total of 25 856 duplicated genes were identified, with whole‐genome duplication (WGD, 62.35%) as the predominant mode, followed by transposed (TRD, 22.26%), dispersed (DSD, 8.78%), tandem (TD, 4.61%), and proximal duplications (PD, 2.00%) (Table S11). TD and PD genes show low synonymous substitution rate (Ks) values, indicating recent duplication and relaxed purifying selection, while PD genes exhibit the highest Ka/Ks, suggesting accelerated sequence evolution (Figure S4B,C). In contrast, WGD genes have the lowest Ka/Ks, reflecting strong functional constraint (Figure S4C). KEGG enrichment analysis of different types of genes revealed that TD genes were significantly enriched in biosynthesis of various plant secondary metabolites, flavonoid metabolism, and tyrosine metabolism (Figures S5–S9).

Assembly quality was comprehensively validated by multiple orthogonal metrics. PanDepth results showed high coverage of different types of sequencing data on each chromosome (Figure S10). The assembly achieved a BUSCO completeness of 98.70% (Table S12), a Merqury consensus quality value (QV = 48.70; per‐base error rate = 1.35 × 10^−5^), and an LTR Assembly Index (LAI = 17.2), all exceeding reference‐quality thresholds. CRAQ analysis yielded near‐perfect accuracy scores (R‐AQI = 99.91, S‐AQI = 100) (Table S13), while OMArk confirmed high completeness (98.43% detected: 60.84% single‐copy, 37.59% duplicated, 1.57% missing) and strong taxonomic consistency (90.27%) (Figure S11).

### 2.3 Comparative Genome and Evolutionary Analysis of *B. chinensis*

Orthogroup analysis across 16 species (14 + 2), including *B. chinensis* , identified 35 037 orthogroups encompassing 92.9% of all predicted genes (Table S14). Of these, 6041 orthogroups were conserved across all species (core orthogroups), while 362 were unique to *B. chinensis* . Within the *B. chinensis* genome, 1757 single‐copy orthologs, 16 670 multi‐copy orthologs, 1222 species‐specific paralogs, and 2236 unclustered genes were annotated (Figure S12A,B; Table S15).

**FIGURE 2:**
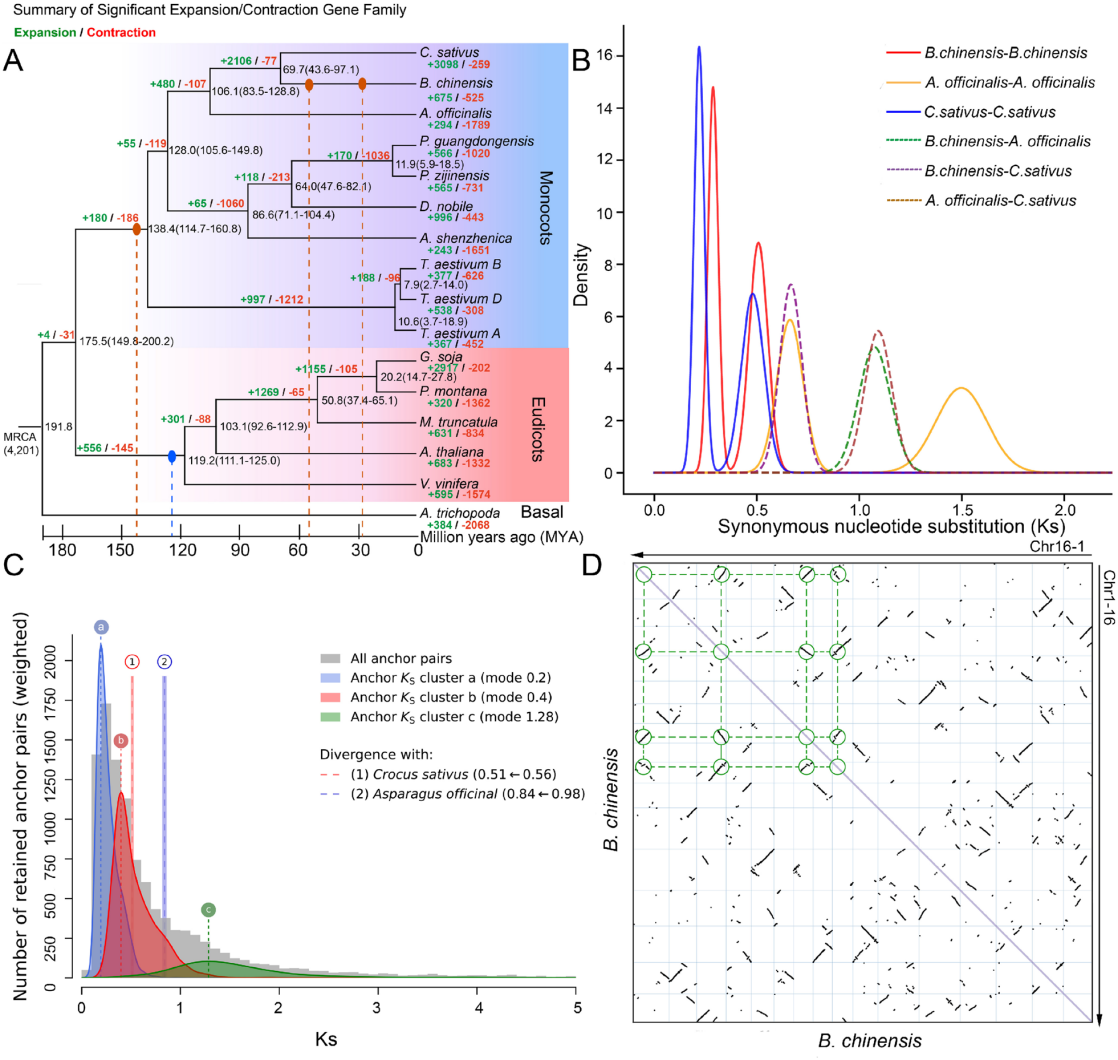
Comparative genomic and evolutionary analysis of *B. chinensis* . (A) Phylogenetic tree of *B. chinensis* and 15 other species, with node ages (MYA) and 95% confidence intervals. Green and red labels indicate significantly expanded and contracted gene families, respectively. Blue and red‐brown ellipses denote whole‐genome triplication (WGT) and whole‐genome duplication (WGD) events inferred from synteny and Ks analyses. (B) Synonymous substitution rates (Ks) for orthologous gene pairs among *B. chinensis* , *Crocus sativus* , and *Asparagus officinalis* , computed using WGDI. (C) Ks distributions of paralogous gene pairs in *B. chinensis* (peaks a, b, c) and orthologous pairs between *B. chinensis* and its close relatives ( *C. sativus* , *A. officinalis* ), generated using Ksrates. The divergent Ks value for orthologs between *B. chinensis* and *C. sativus* was revised from 0.56 to 0.51 (1), while the Ks value between *B. chinensis* and *A. officinalis* was updated from 0.98 to 0.84 (2). (D) Syntenic dot plot of the *B. chinensis* genome (Chr1‐16), generated using JCVI, showing intra‐chromosomal collinearity consistent with two recent WGD events.

Phylogenomic analysis of *B. chinensis* and 15 other species, based on 205 conserved gene families and calibrated with four well‐established fossil constraints, resolved *B. chinensis* as the sister lineage to *C. sativus* (Iridaceae), with a divergence time of ~69.7 MYA (95% CI: 43.6–97.1 MYA) (Figure 2A).

To reconstruct its ancient polyploidy events, we integrated synteny‐anchored Ks distributions, synteny‐depth profiling, and fossil‐calibrated molecular dating. Ks distribution analysis of *B. chinensis* revealed distinct peaks at 0.287 and 0.508 (Figure 2B), suggesting at least two whole‐genome duplication (WGD) events. Using the Ksrates package to adjust evolutionary rates among lineages, we identified three distinct peaks in Ks distributions for all syntenic blocks: 0.2 (a), 0.4 (b), and 1.28 (c) (Figure 2C; Figure S13). These peaks suggest two *B. chinensis* ‐specific WGD events occurring after divergence from *C. sativus* and *A. officinalis* , in addition to a shared monocotyledonous WGD event (τ WGD) (Figure 2C). Using a lineage‐specific synonymous substitution rate of 3.66 × 10^−9^ subs/site/year, derived from the *B. chinensis* ‐ *C. sativus* split, the two Ks peaks correspond to WGD events at approximately 54.6 and 27.3 MYA. Both occurred after the divergence from *C. sativus* , confirming their lineage specificity to *B. chinensis* . This inference is corroborated by syntenic depth analyses: self‐alignment of the *B. chinensis* genome exhibited a predominant 4:4 collinearity pattern, while comparative alignment with the ancestral monocot karyotype (AMK‐A) yielded a 1:8 depth ratio, consistent with three cumulative WGD events (including the shared monocot τ event) (Figure 2D; Figures S14 and S15).

In the Iridaceae species *B. chinensis* and *C. sativus* , the number of significantly expanded gene families exceeded the number of significant contracted gene families. Specifically, *B. chinensis* exhibited significant expansion in 675 gene families and contraction in 525 gene families (Figure 2A). GO enrichment analysis of the expanded families showed strong overrepresentation in biological process (BP) category related to biotic defence, including defence response to oomycetes, defence response to bacterium, and systemic acquired resistance, as well as responses to abiotic stresses such as salt stress and water deprivation. Cellular component (CC) category highlighted enrichment in plant‐type cell wall, plasma membrane, and vacuole, while molecular function (MF) category analysis emphasized protein serine/threonine kinase activity, transmembrane transporter activity, and ubiquitin ligase activity—all critical for signal transduction and immune regulation (Figures S16–S18). KEGG pathway enrichment further confirmed significant expansion in plant secondary metabolism, particularly flavonoid biosynthesis (ko00941) and phenylpropanoid biosynthesis (ko00940), which constitute the core pathways for isoflavone production in *B. chinensis* (Figure S19). These results indicate that lineage‐specific gene family expansions have enriched the functional gene repertoire of *B. chinensis* , providing a genomic basis for its enhanced stress resilience and capacity for specialised metabolite biosynthesis.

### 2.4 TD and WGD Facilitate BcOMTs Replication for Isoflavone Biosynthesis in *B. chinensis*

**FIGURE 3:**
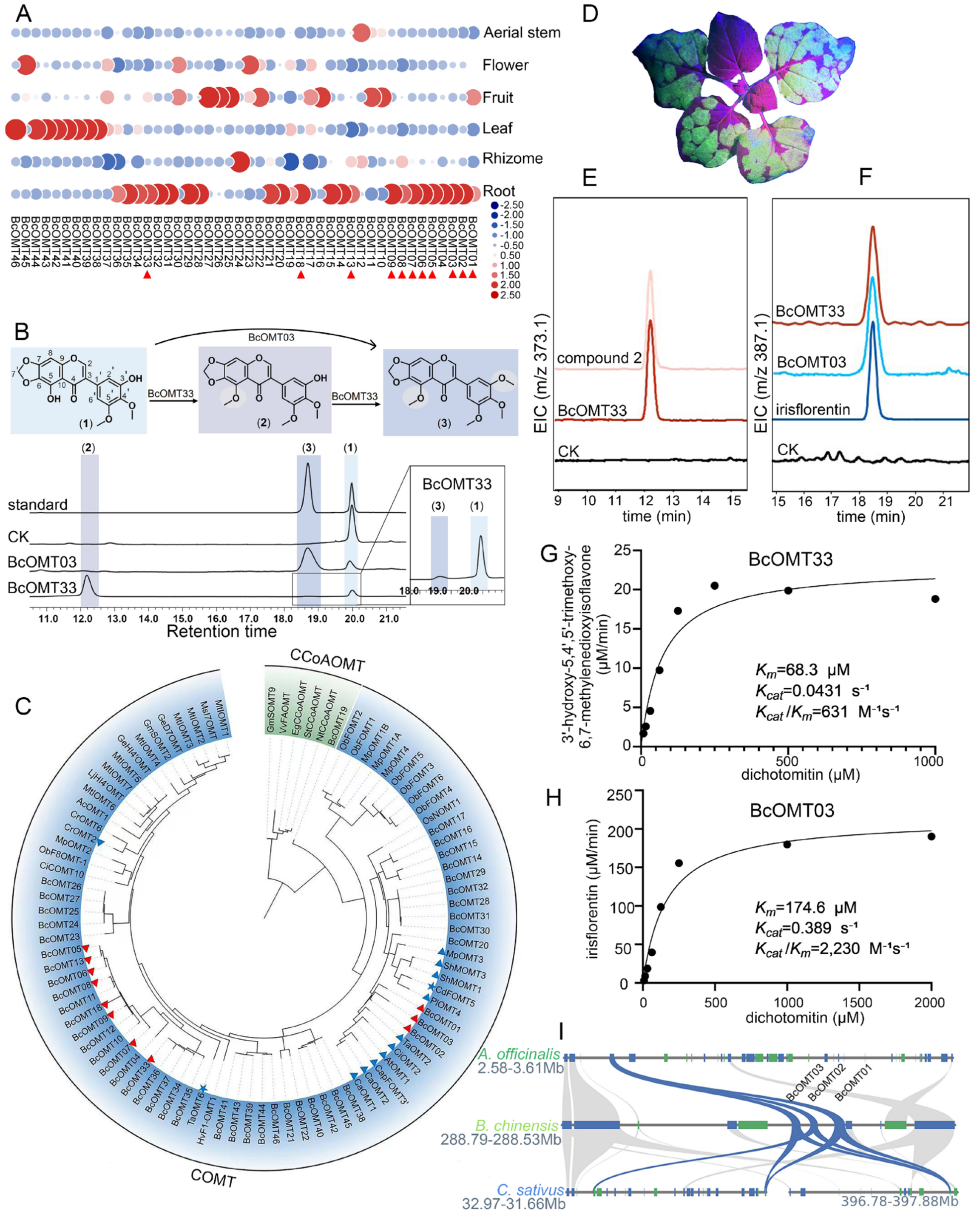
Functional characterisation of BcOMT03 and BcOMT33. (A) Expression heatmap of all BcOMTs in six different organs of *B. chinensis* . Red triangles indicate candidate genes with high root expression. (B) HPLC chromatogram of the in vitro enzymatic reactions of BcOMT03 and BcOMT33 using dichotomitin (1) as the substrate. (C) Phylogenetic tree of BcOMTs and 47 plant OMTs; COMT and CCoAOMT clades highlighted. Blue triangles indicate 3′/5′‐methylation activity; blue pentagrams indicate 5‐O‐methylation activity. (D) *N. benthamiana* was infiltrated with *Agrobacterium* carrying a GFP tag; 4 days later, green fluorescence was observed under UV illumination. Extraction ion chromatogram (EIC) of 3′‐hydroxy‐5,4′,5′‐trimethoxy‐6,7‐methylenedioxyisoflavone (E) and irisflorentin (F) in the extract of *N. benjamina* infiltrated with BcOMT03/BcOMT33. CK = pEAQ‐HT‐GFP empty vector. (G, H) Kinetic analysis of BcOMT33 (G) and BcOMT03 (H) on dichotomitin (1). (I) Microsynteny of the BcOMT01‐03 cluster among *B. chinensis* , *C. sativus* , and *A. officinalis* .

Methylation modification drives structural diversification of isoflavones in *B. chinensis* , such as irisflorentin (**3**). Irisflorentin (**3**) is a quality control compound of *B. chinensis* , which has four oxymethyl groups at C5, C3′, C4′, and C5′ positions. Irisflorentin (3) is predominantly accumulated in the root of *B. chinensis* (Table S22) (Tian et al. 2018). To study the biosynthesis of irisflorentin (**3**), 46 *BcOMTs* were identified and renamed in the *B. chinensis* genome (Table S16). 11 of them exhibited high root‐specific expression (FPKM > 70), and they were prioritised as candidates for irisflorentin biosynthesis (Figure 3A,C; red triangles). In vitro enzymatic assays using dichotomitin (**1**) as substrate and S‐adenosylmethionine (SAM, **12**) as methyl donor revealed that BcOMT03 and BcOMT33 catalyse O‐methylation at C5 and C3′ positions, converting dichotomitin (**1**) to irisflorentin (**3**). BcOMT33 primarily catalyses the formation of 3′‐hydroxy‐5,4′,5′‐trimethoxy‐6,7‐methylenedioxyisoflavone (**2**) with minor production of irisflorentin (**3**) (Figure 3B; Figure S20–S23 Table S17). This complementary activity was further confirmed in *Nicotiana benthamiana* transient expression assays (Figure 3D–F; Figure S24).

Under the optimal reaction conditions (Figure S25), the kinetic analysis of the purified enzymes demonstrated different catalytic efficiencies: BcOMT33 exhibited higher substrate affinity (*K*_*m*_ = 68.3 μM) but a lower conversion (*k*_*cat*_ = 0.0431 s^−1^), resulting in a catalytic efficiency of 631 M^−1^ s^−1^ (Figure 3G); in contrast, BcOMT03 exhibits lower affinity (*K*_*m*_ = 174.6 μM) but a higher turnover rate (*k*_*cat*_ = 0.389 s^−1^), achieving 2.23 × 10^3^ M^−1^ s^−1^ (Figure 3H).

Phylogenetically, all BcOMTs cluster within the COMT superfamily, with BcOMT03 grouping with known 3′/5′‐OMTs, supporting that they may have similar catalytic functions (Figure 3C; Table S18). Chromosome location analysis revealed that 80% of *BcOMTs* are distributed on chromosomes 5, 13, and 14 (Figure S26). Tandem duplication (TD) is prevalent in the *B. chinensis* genome, as evidenced by adjacent BcOMT gene pairs such as *BcOMT01*, *BcOMT02*, and *BcOMT03* on pseudochromosome 1. Collinearity analysis showed that the *BcOMT01‐03* gene cluster is conserved in *B. chinensis* , *C. sativus* , and *A. officinalis* , indicating its origin before the divergence of these species (Figure 3I). To assess the contribution of WGD, the average Ks value of all *BcOMTs* paralogous gene pairs was calculated, yielding a mean of ~0.47, which aligns closely with the timing of the first lineage‐specific WGD event in *B. chinensis* (Table S19). These findings suggest that both TD and WGD events have significantly contributed to the expansion of the *BcOMTs* gene family.

### 2.5 BcUGT009/124/119/032 Are Isoflavone Glycosyltransferases With Substrate Promiscuity

**FIGURE 4:**
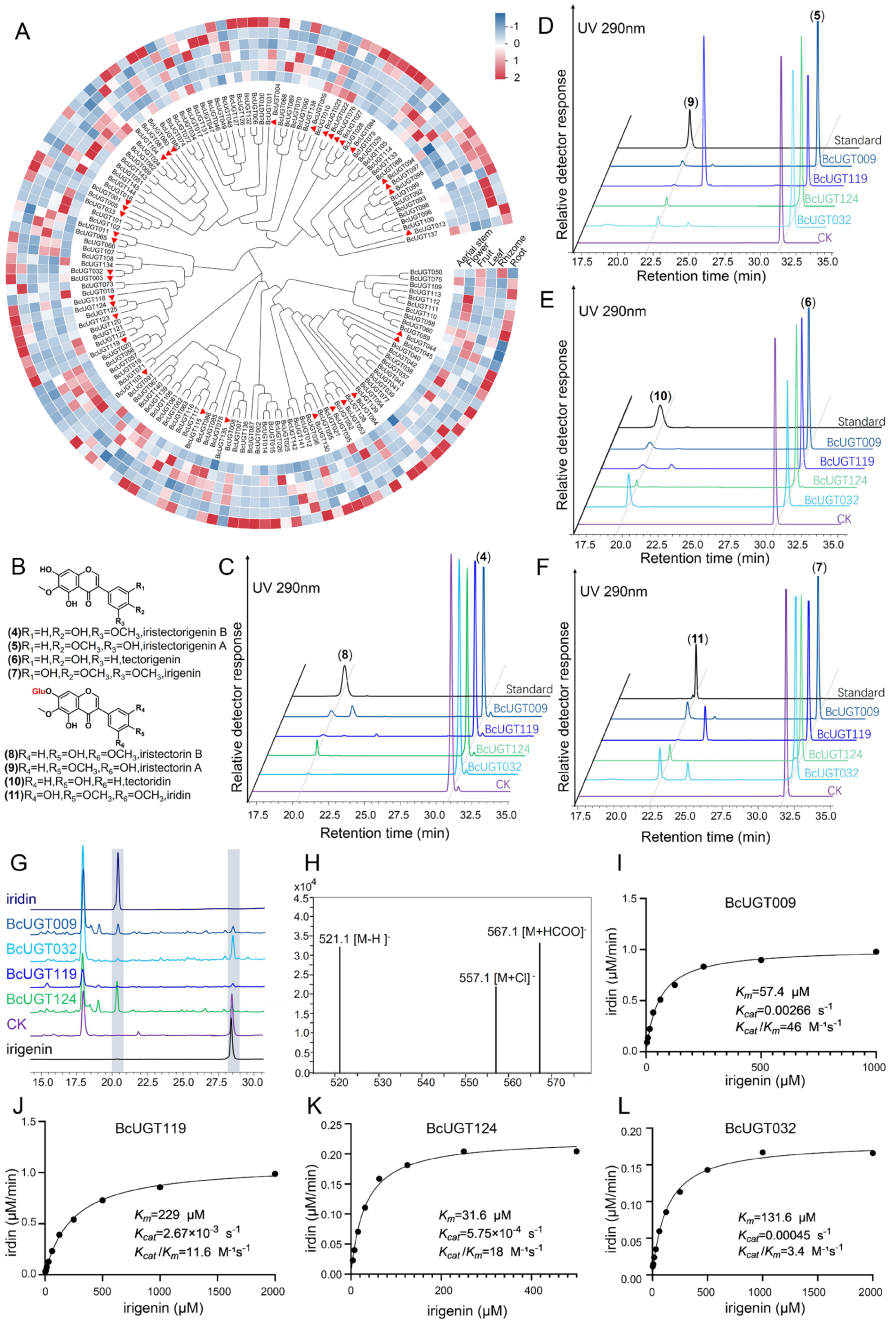
Overview of functional BcUGTs in this study. (A) Expression heatmap of all BcUGTs in six different organs of *B. chinensis* . Red triangles represent candidate BcUGTs selected for functional characterisation. (B) Chemical structures of substrates (4–7) and corresponding glycosylated products (8–11). (C–F) HPLC chromatograms (UV 290 nm) demonstrating that BcUGT009, BcUGT119, BcUGT124, and BcUGT032 enzymatically convert iristectorigenin B (4), iristectorigenin A (5), tectorigenin (6), and irigenin (7) into iristectorin B (8), iristectorin A (9), tectoridin (10), and iridin (11), respectively. CK = negative control (empty vector). (G) HPLC chromatogram of the leaves extract of *N. benthamiana* after transient overexpression of the BcUGT009/124/119/032 gene. (H) LC\MS analysis of iridin in the extract of *N. benthamiana* leaves. (I‐L) Enzyme kinetics analysis of BcUGT009 (I), BcUGT119 (J), BcUGT124 (K), and BcUGT032 (L) using irigenin (7) as substrate.

In addition to methylation modifications, glycosylation plays a crucial role in the biosynthesis and accumulation of bioactive isoflavones in *B. chinensis* , particularly in roots and rhizomes where compounds, such as iristectorin B (**8**), iristectorin A (**9**), tectoridin (**10**), and iridin (**11**) are predominant (Table S22) (Tian et al. 2018). To identify the responsible glycosyltransferases, 145 UGT‐coding genes were annotated and renamed in the *B. chinensis* genome (Table S20). All annotated BcUGTs belong to the GT1 superfamily (Table S21), and ~50% are located on chromosomes 8, 10, 12, and 14 (Figure S26). Phylogenetic analysis revealed that BcUGTs distribute across 19 subfamilies, 5 of which are lineage‐specific (Figure S27). Candidate *UGTs* with high root/rhizome expression (FPKM > 20) were prioritised for functional characterisation (Figure 4A; red triangles).

13 candidate BcUGTs were successfully cloned and expressed in *Escherichia coli* BL21 (DE3). In vitro assays using UDP‐Glu as sugar donor and iristectorigenin B (4), iristectorigenin A (5), tectorigenin (6), and irigenin (7) as substrates demonstrated that BcUGT009, BcUGT119, BcUGT124, and BcUGT032 catalyse *7‐O*‐glucosylation, yielding iristectorin B (8), iristectorin A (9), tectoridin (10), and iridin (11), respectively (Figure 4B–F; Figure S28). However, due to the lack of corresponding standards, we cannot completely verify the product structure. Further work is needed to confirm the specificity and complete characterisation of glycosylation products. Although it was previously reported that BcUGT119 glycosylated tectorigenin and irigenin at *C7* (Zhang et al. 2021), this study extends its substrate spectrum to include iristectorigenin B and iristectorigenin A and further reveals that all four enzymes exhibit broad substrate promiscuity. Transient expression in *N. benthamiana* showed that the corresponding glycosylation product iridin could also be detected by adding substrate irigenin (Figure 4G,H).

Kinetic characterisation under optimised conditions revealed substantial functional differences among the four BcUGTs (Figure S29). BcUGT009 emerged as the most efficient catalyst, with a catalytic efficiency (*k*_*cat*_/*K*_*m*_) of 46 M^−1^ s^−1^, arising from a favourable balance of moderate substrate affinity (*K*_*m*_ = 57.4 μM) and the highest turnover rate (*k*_*cat*_ = 2.66 × 10^−3^ s^−1^). Although BcUGT124 exhibited the strongest substrate binding (*K*_*m*_ = 31.6 μM), its low turnover limited its overall efficiency to 18 M^−1^ s^−1^. In contrast, BcUGT032 and BcUGT119 displayed markedly lower efficiencies (3.4 and 11.6 M^−1^ s^−1^, respectively), with the low efficiency of BcUGT032 attributed to weak substrate affinity (*K*_*m*_ = 131.6 μM) (Figure 4I–L). Collectively, these results identify BcUGT009 as the most efficient and broadly active glycosyltransferases with 7‐O catalytic activity among the characterised BcUGTs, suggesting that it is likely the predominant contributor to isoflavone glycosylation in *B. chinensis* under physiological conditions.

The collinearity analysis of these four glycosyltransferases showed that compared with *BcUGT009* and *BcUGT032*, *BcUGT119* and *BcUGT124* from the same gene subfamily were not collinear with dicotyledons, which may indicate that they had independent evolution of monocotyledons (Figure S30). These enzymes play important roles in the *B. chinensis* isoflavone glycosides.

### 2.6 Molecular Mechanism of UDP‐Glu Glycosyl Transfer Catalysed by BcUGT009

**FIGURE 5:**
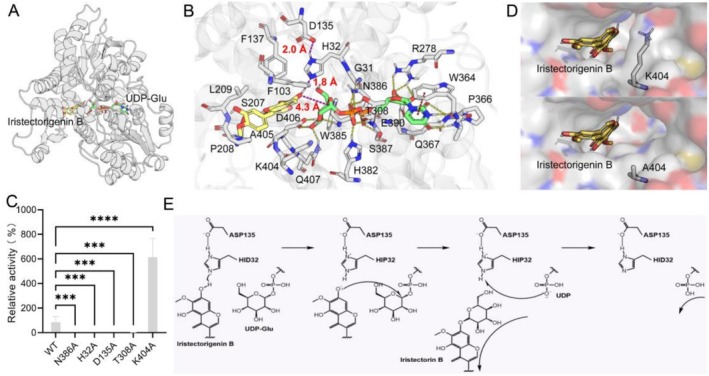
Structural basis underlying the glycosylation mechanism of BcUGT009. (A) Predicted ternary complex of BcUGT009/iristectorigenin B/UDP‐Glu. (B) The binding mode of iristectorigenin B and UDP‐Glu shows a suitable near‐attack conformation. The purple dotted line represents the key hydrogen bond distance and nucleophilic attack distance during the glycosylation transfer process. The yellow dotted line indicates the hydrogen bond interaction between UDP‐Glu and amino acid residues. The red dotted line represents the π‐π stacking interaction. (C) Changes in the relative catalytic activities of wild‐type BcUGT009 and its mutants after adding the same dose of iristectorigenin B. Statistical significance was determined by one‐way ANOVA with Dunnett's multiple‐comparison correction against the wild type as a common control. Data are presented as mean ±95% CI of three independent biological replicates (*n* = 3), with asterisks indicating ****p < 0.0001. (D) Comparison of substrate channels between wild‐type BcUGT009 (top) and BcUGT009‐K404A mutant (bottom). (E) Glycosylation mechanism of BcUGT009.

To analyse the molecular mechanism by which BcUGT009 catalyses the glycosylation transfer between iristectorigenin B and UDP‐Glu, we first constructed the binary complex structure of BcUGT009/UDP‐Glu through Boltz2. Then, we used AutoDock Vina to dock iristectorigenin B into the substrate pocket of the binary complex, and selected the appropriate near‐attack conformation for analysis (Figure 5A). As shown in Figure 5B, UDP‐Glu stably binds through hydrogen bonds and π‐π stacking interactions with surrounding amino acid residues, with its glucose portion pointing toward the binding site of the substrate. Targeted mutagenesis showed that, with N386A and T308A, product formation by BcUGT009 was almost abolished (Figure 5C), underscoring the essentiality of these residues for UDP‐Glu conversion. The hydroxyl group at the glycosylation site of iristectorigenin B forms a hydrogen bond network with H32 and D135. The steric hindrance of the surrounding residues restricts the conformational changes of the substrate, which efficiently locates the glycosylation site to the catalytic center. The results of site‐directed mutagenesis indicated that the mutations of H32A and D135A would also cause a complete loss of enzyme activity (Figure 5C), suggesting that H32 is a catalytic base and D135 is a catalytic pair that may stably protonate H32. Further analysis of the substrate channel indicated that the steric hindrance of K404 limited the size of the pocket, making it difficult for the glycosylation site of the substrate to approach the glycosylation donor (4.3 Å). The computational mutation of K404A could significantly increase the substrate channel (Figure 5D). To verify this, we conducted a site‐directed mutagenesis experiment on BcUGT009‐K404A. The results indicated that theproduct formation of this mutant was 2.3–7.2 times higher than that of the wild type (Figure 5C; Figure S31), enabling a more efficient glycosylation transfer reaction. Based on these results, we propose the catalytic mechanism of BcUGT009 (Figure 5E). Among them, H32, as a catalytic base, removes the proton from the glycosylation site of iristectorigenin B to enhance its nucleophilicity, and D135 can stabilise the protonated catalytic histidine. The generated nucleophilic receptor then attacks the C1 of UDP‐Glu to replace UDP and form the product.

### 2.7 Gene Family Expansion and Isoflavone Biosynthesis in Legumes and Non‐Leguminous Plants

**FIGURE 6:**
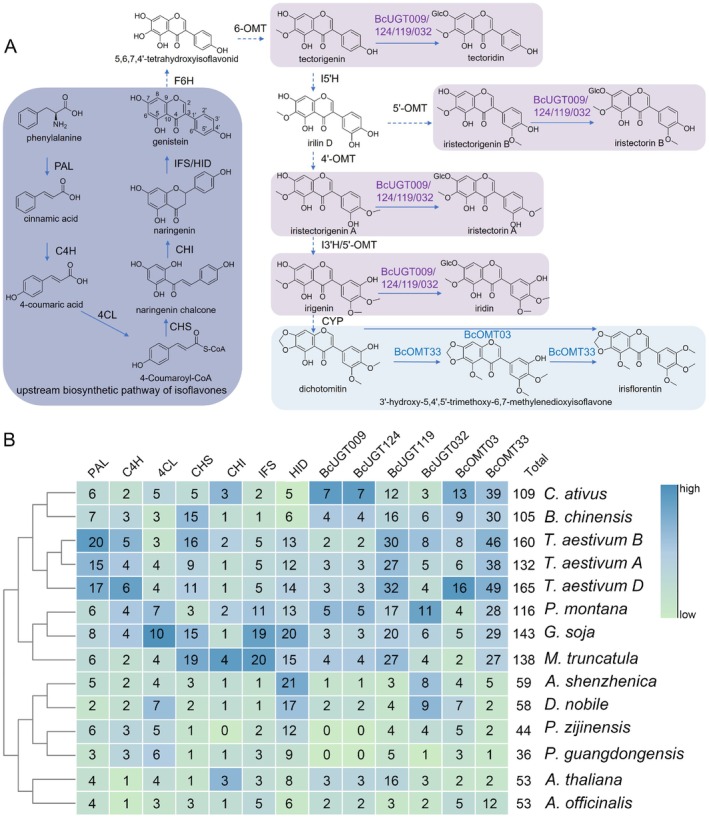
Isoflavone biosynthesis and genetic basis in *B. chinensis* and other species. (A) The isoflavone biosynthetic pathway in *B. chinensis* . This diagram illustrates the metabolic route from phenylalanine to various end products, encompassing both upstream skeleton formation and downstream specific modification steps. (B) Comparison of homologous gene copy numbers for the isoflavone pathway across different plant species.

Although isoflavones have long been regarded as symbolic secondary metabolites of legumes, they also exist in several non‐leguminous lineages such as Iridaceae and Poaceae. To clarify the taxonomic distribution of their biosynthetic capacity, we first reconstructed the isoflavone pathway in *B. chinensis* (Figure 6A). Starting from phenylalanine, the route proceeds through the general phenylpropanoid enzymes phenylalanine ammonia‐lyase (PAL), cinnamate‐4‐hydroxylase (C4H), and 4‐coumaric acid coenzyme A ligase (4CL), followed by the flavonoid core enzymes chalcone synthase (CHS) and chalcone isomerase (CHI) to yield naringenin. A pivotal branch point is governed by isoflavone synthase (IFS), which redirects the flux toward the isoflavone branch. Subsequent tailoring reactions include hydroxylation catalysed by cytochrome P450 mono‐oxygenases such as isoflavone 3′‐hydroxylase (I3′H) and isoflavone 5′‐hydroxylase (I5′H), O‐methylation by O‐methyltransferases, and glycosylation by UDP‐dependent glycosyltransferases, which together generate the characteristic isoflavones such as tectorigenin and iridin.

To assess whether a shared genetic basis underlies this biosynthetic capability, we surveyed orthologous and paralogous genes within 13 core pathway enzyme families across 12 phylogenetically diverse plant genomes (Wang et al. 2022; Meng et al. 2022). As shown in Figure 6B, species that accumulate isoflavones, including *P. montana* , *G. soja* , *M. truncatula* , *B. chinensis* , and *T. aestivum* , tend to have a higher number of gene copies in these biosynthetic families compared to those that lack the metabolite. This expansion is observed in both legumes and non‐legumes, suggesting that the genetic framework for isoflavone biosynthesis may not be limited to legumes, but could involve the convergent or parallel expansion of key enzyme families. However, this association requires further experimental validation, including evidence from metabolomic and genomic data, to better understand its role in isoflavone biosynthesis.

## 3 Discussion

High‐quality genome assembly is essential for understanding how species evolve and develop new biological functions. We present a near‐T2T genome assembly of *B. chinensis* (4.18 Gb). This resource enabled us to trace the origins of its expanded genome size and dissect the genetic mechanisms underlying characteristic isoflavone diversification, providing new insights into how specialised metabolic pathways evolve in non‐leguminous plants. As a limitation, while centromere positions are supported by Hi‐C interaction patterns and centromere‐repeat FISH, future CENH3‐based mapping would be valuable to further refine the boundaries of active centromeric chromatin when species‐specific reagents become available.

### 3.1 Recent Transposon Bursts and Ancient WGD Events Jointly Drove the Notable Expansion of the *B. chinensis* Genome

The genome of *B. chinensis* (4.18 Gb) ranks among the larger genomes sequenced in angiosperms, a feature shaped by the synergistic effects of repetitive sequence amplification and polyploidization events. More than 90% of the genome consists of repetitive sequences, with LTR retrotransposons—particularly *Gypsy* elements—dominating a major amplification wave within the last 6 million years, directly contributing to the physical enlargement of the genome. More importantly, Ks peak analysis and synteny‐depth scanning revealed two distinct, lineage‐specific whole‐genome duplication (WGD) events, dated to approximately 54.6 and 27.3 MYA. These timings align closely with major global climatic upheavals (the Palaeocene‐Eocene Thermal Maximum and the Oligocene‐Miocene transition), making them compatible with the “stress‐driven polyploidization” hypothesis (Zachos et al. 2008). This correspondence is correlational, however, and alternative drivers of polyploidization cannot be excluded. In this scenario, genomic redundancy resulting from WGD furnishes the raw material for subsequent adaptive evolution. Thus, the relatively large genome size of *B. chinensis* is a historical outcome shaped by both recent transposon activity and ancient environmental pressures, which collectively provided ample sequence space for subsequent functional innovation.

### 3.2 Expanded Stress‐Responsive and Metabolic Gene Families Constitute the Genetic Basis of Chemical Defence in *B. chinensis*

Genomic redundancy created favourable conditions for the expansion and neofunctionalization of gene families. Our study found that the significantly expanded gene families in *B. chinensis* are not randomly distributed but are highly enriched in two major functional modules: basic stress response and specialised metabolic biosynthesis. The former includes genes involved in defence responses against pathogens (e.g., oomycetes, bacteria), abiotic stress tolerance, and signal transduction; the latter is precisely directed toward the phenylpropanoid and flavonoid biosynthetic pathways. This coordinated expansion strategy represents an efficient evolutionary adaptation. On the one hand, an enhanced perception and signalling network enables the plant to respond rapidly to environmental challenges. On the other hand, an expanded biosynthetic capacity allows for the massive production of structurally diverse isoflavones, such as tectoridin and tectorigenin.

### 3.3 Gene Family Expansion and Divergent Isoflavone Biosynthesis Pathways in Non‐Leguminous Plants

In *C. sativus* , while genes related to flavonoid and isoflavone biosynthesis have expanded, this expansion does not result in significant isoflavone accumulation. Instead, the plant focuses on carotenoid biosynthesis, which plays a critical role in photoprotection and pollinator attraction, rather than in chemical defence through isoflavones. In contrast, *B. chinensis* accumulates significant amounts of isoflavones, such as tectoridin and tectorigenin, which play crucial roles as chemical defences against pathogens and herbivores in its dry slope habitat. The expansion of gene families involved in isoflavone biosynthesis in *B. chinensis* likely facilitates the production of these defence compounds, supporting the idea that this plant uses isoflavones for chemical defence rather than for attracting pollinators, as seen in *C. sativus* . Despite the expansion of isoflavone‐related genes in both species, their different metabolic outputs suggest that gene expansion may not always directly correlate with metabolite accumulation. In *B. chinensis* , the Isoflavone Synthase (IFS) gene, which is homologous to leguminous IFS, may function differently than the typical IFS found in legumes. The single copy of the IFS gene in *B. chinensis* may be adapted to serve the plant's chemical defence needs, but its mechanism could be distinct from that in legumes. Further studies are needed to investigate whether *B. chinensis* utilises a unique IFS enzyme or an alternative metabolic pathway for isoflavone production.

In summary, this study outlines a coherent narrative of adaptive evolution: environmental stresses likely triggered ancestral WGD events in *B. chinensis* , after which the genome underwent further expansion via transposon activity. The resulting genetic redundancy was then shaped by natural selection, enhancing basal stress resistance on one hand, and refining an efficient isoflavone synthesis and modification pathway on the other. This genome resource will enable reverse genetics coupled with untargeted metabolomics and population‐scale haplotype‐metabolite association studies to strengthen functional assignments and accelerate breeding applications.

## 4 Materials and Methods

### 4.1 Genome Sequencing, Assembly, and Validation

#### 4.1.1 Plant Material and Nucleic Acid Preparation

Two‐year‐old *B. chinensis* was collected in the Botanic Garden of China Pharmaceutical University (Nanjing, China). Fresh leaves were harvested for high‐molecular‐weight (HMW) genomic DNA extraction using an optimised CTAB method. DNA integrity was confirmed by pulsed‐field gel electrophoresis, and purity/concentration were assessed using NanoDrop One, Qubit 3.0 Fluorometer, and 0.75% agarose gel electrophoresis.

#### 4.1.2 Genome Sequencing

Long‐read data were generated on both Oxford Nanopore and PacBio HiFi platforms; Hi‐C libraries were quantified and sequenced on the DNBSEQ platform with paired‐end 350‐bp reads. For rapid genome characterisation, raw DNBSEQ reads were trimmed with Trimmomatic v0.39 (Bolger et al. 2014) to remove adapters and low‐quality bases. Clean reads were then analysed with Kmerfreq v4.0 and GCE v1.0.2 (Liu et al. 2013) to estimate optimal k‐mer, genome size, repeat content, and heterozygosity, while ploidy was assessed using Smudgeplot v0.2.5 (Ranallo‐Benavidez et al. 2020).

#### 4.1.3 Genome De Novo Assembly

HiFi and > 50 kb ONT reads were assembled with Hifiasm v0.19.7‐r598 (Cheng et al. 2021); redundant contigs were removed after remapping with Minimap2 v2.26 (Li 2021) and Khaper (https://github.com/lardo/khaper). The primary contig assembly produced by Hifiasm exhibited an assembly BUSCO completeness of 98.7% v5.4.7 (Manni et al. 2021) and was used as the backbone for subsequent scaffolding.

Hi‐C reads were cleaned with Trimmomatic, aligned to the contigs with Bwa‐mem2 v2.2.1 (Li 2013), and parsed into valid pairs with Pairtools v1.0.3 (Open2C et al. 2024). Contigs were anchored and oriented into 16 pseudomolecules with YaHS v1.1 (Zhou et al. 2023). Contact maps were generated with Juicer v1.6 (Durand et al. 2016) and manually curated in Juicebox v1.11.08; valid versus mis‐joined interactions were quantified with HiC‐Pro v3.1.0 (Servant et al. 2015) and visualised with plotHicGenome v0.1.0 (https://github.com/Atvar2/plotHicGenome). Remaining gaps were closed with QuarTeT v1.2.1 (Lin et al. 2023). Finally, cleaned HiFi and ONT reads were remapped to the scaffolded assembly with minimap2, and two rounds of error correction were applied with NextPolish2 v0.2.0 (Hu et al. 2024) to yield the near‐T2T genome of *B. chinensis* .

#### 4.1.4 Telomeres and Centromeres Annotation

Telomeric repeats were first detected with QuarTeT; reads carrying these motifs were extracted from both HiFi and ONT data, aligned to the assembly with minimap2, and the telomeric gaps were closed semi‐manually. Centromeres were inferred by scanning the genome for centromere‐associated tandem arrays with pyTanFinder (Kirov et al. 2018) and BLAST+ v2.15.0 (Camacho et al. 2009), followed by cross‐validation against the Hi‐C contact map to pinpoint the most likely centromeric position on each chromosome.

#### 4.1.5 Cytogenetic Validation

Root tips were pretreated with p‐dichlorobenzene/α‐bromonaphthalene (2 h), hypotonized (0.075 M KCl, 10 min), fixed (Carnoy, 3:1), and stored at −20°C. Metaphase spreads were obtained by cellulase‐pectinase digestion (2%–1%, 37°C, 30–60 min) followed by squash and air dry. Slides were pepsin treated (1 ng μL^−1^, 0.01 N HCl, 37°C, 2 h), post fixed (4% paraformaldehyde, 3 min), and dehydrated. FISH used FAM‐labelled telomere probe (AACCCT)_3_ and Cy3‐labelled centromere probe (RepeatExplorer2 tandem repeat) in 50% formamide/2 × SSC/10% dextran sulfate at 37°C overnight; washes in 2 × SSC at 42°C. Chromosomes were counterstained with DAPI (0.5 μg mL^−1^) and captured on an Olympus BX53/BX63 microscope.

#### 4.1.6 Genome Quality Assessment

The sequencing data of WGS (Survey), HiFi, ONT, and Hi‐C were mapped to the genome, and the mapping rate and coverage rate were calculated by PanDepth v2.25 (Yu et al. 2024) to evaluate the accuracy of the genome. Genome quality was assessed with Meryl v1.4.1 and Merqury v1.3 (Rhie et al. 2020), while CRAQ v1.0.9 (Li, Xu, et al. 2023) was employed to evaluate single‐base accuracy. The LTR Assembly Index (LAI) was calculated using LAI vbeta3.2 (Ou and Jiang 2018) to assess genome assembly quality based on repeat sequences. OMArk v0.3.0 (Nevers et al. 2025) was used to evaluate genome completeness and taxonomic consistency against the Viridiplantae reference. Additionally, BUSCO analysis using the “embryophyta_odb10” database was performed to evaluate both the genome assembly accuracy and the completeness of annotated protein sequences.

### 4.2 RNA‐Seq Data Source and Expression Quantification

RNA‐seq data were retrieved from a previously published organ transcriptome of *B. chinensis* (Tian et al. 2018; BioProject PRJNA430284), covering six organs (root, rhizome, aerial stem, leaf, flower, and fruit) with three biological replicates from three independent 3‐year‐old plants. Libraries were strand‐specific and sequenced on Illumina HiSeq 2500 (125‐bp paired‐end). Read preprocessing followed Tian et al. (2018). Adapters and low‐quality bases were removed using Trimmomatic, and rRNA reads were filtered with RiboDetector. Clean paired‐end reads were then quantified against the reference transcriptome using Salmon v1.10.1. Transcript abundances were summarised to the gene level and reported as FPKM values for each annotated gene/candidate gene.

### 4.3 Metabolomics Data Source and Metabolite Distribution Summary

Metabolite profiling data for *B. chinensis* were obtained from a previously published study (Tian et al. 2018), in which isoflavones were quantified across six organs. In the present study, these published metabolomics results were not re‐generated experimentally; instead, they were used as independent evidence of tissue‐enriched metabolite accumulation to support pathway reconstruction and candidate‐gene prioritisation based on genome‐anchored expression profiles. To facilitate transparency and avoid redundancy, key isoflavone/isoflavone‐glycoside distribution patterns across organs were summarised from the published dataset and compiled in Table S22, which is referenced in the corresponding Results sections.

### 4.4 Genome Annotation and Evolutionary Analysis

#### 4.4.1 LTR‐RTs Insertion Time Estimation

Repetitive sequences in the *B. chinensis* genome were annotated using EDTA v2.1.0, a robust tool validated across multiple plant species. To identify and classify long terminal repeat retrotransposons (LTR‐RTs), we used LTR_Finder v1.07 (Xu and Wang 2007), GenomeTools v1.6.3 (https://github.com/genometools/genometools), and LTR_retriever v2.9.0. LTR‐RT pairs were extracted using Bedtools v2.30.0 (Quinlan and Hall 2010), and sequence alignments were performed with MAFFT v7.520 (Rozewicki et al. 2019) to identify mutations. The insertion time of LTR‐RTs was calculated using the formula T = K/(2 × *r*), where r is the nucleotide mutation rate (7e^−9^, based on *A. thaliana* ), and K is the average number of substitutions per site. The R v4.4.1 (R Core Team 2020) package was used for these calculations.

#### 4.4.2 Gene Prediction and Functional Annotation

Non‐coding RNA annotation was performed using Infernal v1.1.5 (Nawrocki and Eddy 2013) for miRNA and snRNA based on the Rfam database, tRNAscan‐SE v2.0.12 (Chan et al. 2021) for tRNA, and Barrnap v0.9 (https://github.com/tseemann/barrnap) for rRNA.

Protein‐coding genes were predicted using three approaches: (1) ab initio prediction with Augustus v3.5.0 (Stanke et al. 2008) and Helixer v0.3.2 (Holst et al. 2023); (2) homology‐based prediction with Miniprot v0.13 r248 (Li 2023) using protein annotations from closely related species (e.g., *Iris pallida* , *Apostasia shenzhenica*, and *Dendrobium nobile*); (3) RNA‐seq‐aided analysis with HISAT2 v2.2.1 (Kim et al. 2019), StringTie v2.2.1 (Shumate et al. 2022), and TransDecoder v5.7.1 (Bushmanova et al. 2019), as well as de nove transcriptome assembly with Trinity v2.15.1 (Grabherr et al. 2011). PASA v2.5.3 (Haas et al. 2003) was used to map assembled transcripts to the genome, and EVidenceModeler v2.1.0 (Haas et al. 2008) integrated the results from these methods into a comprehensive gene structure.

Functional annotation of protein‐coding genes was performed by aligning the whole‐genome protein sequences against local Nr, KEGG, SwissProt, and other databases using DIAMOND v2.1.9 (Buchfink et al. 2021). Additionally, protein sequences were annotated against the Pfam, GO, and KO databases using PfamScan, InterProScan, and KofamScan, respectively.

#### 4.4.3 Comparative Genome

To infer the evolutionary position of *B. chinensis* , we selected 13 representative plant species with sequenced genomes: *Apostasia shenzhenica* (GCA_002786265.1), *Asparagus officinalis* (GCA_001876935.1), *Dendrobium nobile* (GCA_022539455.1), *Glycine soja* (GCA_004193775.2), *Medicago truncatula* (GCA_000219495.2), *Platanthera guangdongensis* (GCA_039583875.1), *Platanthera zijinensis* (GCA_039513925.1), *A. trichopoda* (GCA_000471905.1), *Pueraria montana* (PRJCA016835), *Arabidopsis thaliana* (PRJCA007112), *Vitis vinifera* (PRJCA012093), *C. sativus* (https://doi.org/10.6084/m9.figshare.21988667), *Triticum aestivum* (http://plants.ensembl.org), among which *A. trichopoda* as an outgroup. OrthoFinder v2.5.5 (Emms and Kelly 2019) identified orthologous genes across the 16 (14 + 2) species, including three sub‐genomes of *T. aestivum* . We extracted 205 common conserved gene families from single‐copy genes of the 16 species and two‐copy genes of *C. sativus* and *G. soja* (Chen et al. 2020). The amino acid sequences of these gene families were aligned using PRANK v170427 (Löytynoja 2021), and the alignments were converted into codon alignments by PAL2NAL v14 (Suyama et al. 2006). ModelTest‐NG v0.1.7 selected the best model for phylogenetic analysis, and RAxML‐NG v1.2.2 (Kozlov et al. 2019) constructed the maximum likelihood (ML) phylogenetic tree. ASTRAL v5.7.8 integrated gene trees from 205 common conserved genes. The divergence time of the above 16 species was estimated by the Mcmctree program from PAML v4.10.7 (Yang 2007). The following higher‐level divergence nodes were used as calibration points (Kumar et al. 2022): the Rosids‐Cucurbitales split (109.8–124.4 MYA, represented by *V. vinifera* and *P. montana* ), the Fabales‐Rosales split (17.6–54.3 MYA, represented by *P. montana* and *G. soja* ), the Monocots‐Dioscoreales/Pandanales grade (72.2–114.1 MYA, represented by *P*. *zijinensis* and *A*. *shenzhenica*), and the angiosperm crown node (179.9–205.0 MYA, represented by *A. trichopoda* and *T. aestivum* ). Expansion and contraction of orthologous gene families in *B. chinensis* were analysed using the CAFE5 v1.1 (Mendes et al. 2020) model, and the results were visualised with CafePlotter v0.1.1 (https://github.com/moshi4/CafePlotter/). KEGG and GO enrichment analyses of significantly expanded and contracted genes were performed using the ClusterProfiler v4.6.2 (Wu et al. 2021) R package.

For evolutionary analysis of specific enzyme families (e.g., BcOMTs, BcUGTs), protein sequences were aligned using MAFFT, and the alignments were trimmed with trimAl v1.4.1 (Capella‐Gutiérrez et al. 2009). Maximum‐likelihood phylogenetic trees were constructed using IQ‐TREE v2.2.2.7 (Nguyen et al. 2015) and visualised with Evolview (Subramanian et al. 2019). Tissue‐specific expression patterns (FPKM values) for candidate genes were visualised as heatmaps using TBtools‐II (Chen et al. 2023).

OrthoFinder v 2.5.5 (Emms and Kelly 2019) was used to identify the orthologous genome shared by *B. chinensis* with 13 other species, and the threshold value was 1e^−5^ and the similarity was 70%.

#### 4.4.4 Whole‐Genome Duplication (WGD) Analysis

WGDI v0.6.5 (Sun et al. 2022) was used to infer duplication events of *B. chinensis* , *A. officinalis* , *and C. sativus* with their ancestral monocot karyotype (AMK‐A). Genomic synteny dot plots were generated by WGDI, and Ks values between homologues were calculated. The formula “T = Ks/2*r*” was used to calculate the divergence time when WGD events occur (where “Ks” represents the synonymous substitution rate and “*r*” represents the rate of synonymous substitutions per year per site). Since the rate of evolution differs significantly among species, we employed Ksrates v1.1.4 (Sensalari et al. 2022) to adjust the Ks values, ensuring more precise identification of whole‐genome duplications and divergence times among the studied species. Another method used JCVI v1.4.16 (Tang et al. 2024) for whole‐genome syntenic analysis.

#### 4.4.5 Duplication Mode Classification

BLAST+ was used to search and align the gene and protein sequences of *B. chinensis* (E value < 1 × 10^−10^). Then, using the stricter version of DupGen_finder (Qiao et al. 2019), DupGen_finder.unique.pl, different types of duplicate genes in *the B. chinensis* genome were classified. The software KaKs_Calculator v3.0 (Zhang 2022) estimated the Ka (number of substitutions per nonsynonymous site), Ks (number of substitutions per synonymous site), and Ka/Ks values of gene pairs produced by different gene replication in *B. chinensis* .

### 4.5 Enzyme Identification, Characterisation, and Validation

#### 4.5.1 Candidate Gene Screening

To annotate gene domains in the *B. chinensis* genome, PfamScan v1.6 was used with the Pfam v36.0 database (http://ftp.ebi.ac.uk/pub/databases/Pfam), screening for BcOMTs and BcUGTs containing PF01596 and PF00201 domains, respectively. CAZy family assignment was performed with dbCAN3 using HMMER (Zheng et al. 2023), applying an E‐value threshold of 1e‐15 and coverage ≥ 0.35.

#### 4.5.2 Heterologous Expression and Protein Purification

Candidate BcOMTs and BcUGTs were subcloned into the expression vector pET‐28a through BamHI restriction sites to construct the recombinant plasmids (primers in Tables S23 and S24), and these plasmids were transformed into *E. coli* BL21 (*DE3*) competent cells. Recombinant strains were cultured in LB medium containing 100 μg/mL kanamycin at 37°C until the OD_600_ reached 0.6. Isopropyl‐β‐D‐thiogalactopyranoside (IPTG) was then added to a final concentration of 0.2 mM, and the culture was continued at 17°C overnight. Cells were collected by centrifugation at 5000 rpm for 20 min at 4°C.

The cell pellet was resuspended in 50 mL ice‐precooled 50 mM Tris–HCl buffer (pH 8.0) and crushed using an ultrasonic crusher (Scientz‐IID, China). The cell debris was centrifuged at 10000 rpm for 30 min at 4°C to obtain the supernatant. To purify the recombinant protein with a 6‐His tag, the supernatant was loaded onto a nickel affinity chromatography column through a 0.8 μm filter. Wash the column with 15 mL of 50 mM Tris–HCl buffer (pH 8.0) containing 20 mM imidazole and 15 mL of 50 mM Tris–HCl buffer (pH 8.0) containing 50 mM imidazole, respectively.

Proteins were eluted with 15 mL elution buffer (50 mM Tris–HCl, pH 8.0, containing 250 mM imidazole). The eluted protein was concentrated by using an Amicon ultracentrifuge filter (Biomax‐30; Millipore, USA).

#### 4.5.3 Enzyme Assays of BcOMTs and BcUGTs

Enzymatic assays for BcOMTs were conducted in 150 μL reactions containing 50 μL purified recombinant protein, 2 mM dichotomitin, 2 mM SAM, and 50 mM Tris–HCl buffer (pH 8.0) to complete the volume. For BcUGTs, the assays were performed in 200 μL reactions with 95 μL purified recombinant protein, 2 mM substrate (iristectorigenin A, iristectorigenin B, tectorigenin, irigenin), and 3 mM UDP‐Glu. Both reaction mixtures were incubated at 37°C for 12 h and terminated by adding an equal volume of methanol. Negative controls were prepared using *E. coli* BL21 (*DE3*) transformed with the empty pET‐28a vector.

#### 4.5.4 Detection of Catalytic Reaction Products

High Performance Liquid Chromatography (HPLC) was performed using a Shimadzu LC‐2050C 3D system (Japan) with a C18 column (5 μm, 4.6 × 250 mm; Agilent). The flow rate was 1.0 mL/min, the column temperature was 30°C, and the detection wavelength was set at 270 nm. Gradient elution conditions for BcOMTs and BcUGTs reactions are detailed in Tables S25 and S25, respectively. Semi‐preparative HPLC was conducted using a Zorbax SBC18 column (5 μm, 9.4 × 250 mm; Agilent).

Reaction products were further analysed using Agilent 1290 UPLC/6540 Q‐TOF LC/MS, with chromatographic conditions as described above but using pure water instead of formic acid water. All NMR spectra (^1^H, ^13^C, HSQC, COSY, HMBC, NOESY) for compound **2** were acquired on a Bruker ASCEND 800 MHz spectrometer, with tetramethylsilane (TMS) serving as the internal reference standard.

#### 4.5.5 Biochemical and Kinetic Characterisation

The enzymatic activities of BcOMT03 and BcOMT33 were assayed with dichotomitin as methyl acceptor and SAM as methyl donor, while BcUGT009/124/119/032 were tested using irigenin and UDP‐Glu as substrates. Reactions (150 μL) contained 50 μg purified enzyme in 50 mM Tris–HCl buffer (pH 5–10) and were incubated at 37°C for 2 h to determine optimal pH. Protein concentrations were determined with the BCA Protein Quantification Kit (Abkine, Wuhan, China) according to the manufacturer's instructions. Temperature optima were assessed by incubating reactions at 20°C–50°C (in 5°C increments) under optimal pH for 2 h.

For kinetic analysis, initial velocities were measured at 30 min with dichotomitin or irigenin concentrations ranging from 7.8125 to 2000 μM, while SAM or UDP‐Glu was held at saturating levels (1 mM). Reactions were quenched with methanol, and product formation was quantified by HPLC. Kinetic parameters (*K*_*m*_, *K*_*cat*_ and *K*_*cat*_ */K*_*m*_) were derived by fitting data to the Michaelis–Menten equation using nonlinear regression in GraphPad Prism 10.1.2 (GraphPad, USA).

#### 4.5.6 Transient Expression in *N. Benthamiana*

Transient expression assays were performed in *N. benthamiana* to validate the in planta activity of BcOMTs and BcUGTs. The coding sequences of BcOMT03, BcOMT33, BcUGT009, BcUGT119, BcUGT124, and BcUGT032 were subcloned into the vector pEAQ‐HT (primers in Table S27). Constructs were transformed into *Agrobacterium tumefaciens* GV3101 and verified by Sanger sequencing (Tsingke Biotech).

For infiltration, *Agrobacterium* cultures were grown at 28°C for 24 h, resuspended in infiltration buffer (10 mM MES, 10 mM MgCl_2_, 200 μM acetosyringone, pH 5.6), and adjusted to OD_600_ = 0.2. After 2 h of incubation at room temperature, cultures were infiltrated into leaves of 5‐week‐old *N. benthamiana* plants using a needleless syringe (Laforest et al. 2025). Since *N. benthamiana* lacks endogenous isoflavone precursors, 40 μM of dichotomitin (for BcOMT assays) or 40 μM of irigenin (for BcUGTs assays) was infiltrated into the same leaf areas 4 days post‐Agrobacterium infiltration. Leaf samples were harvested 2 days later, flash‐frozen in liquid nitrogen, and stored at −80°C for LC–MS analysis. An empty vector was used as a negative control, and protein expression was monitored under UV light.

#### 4.5.7 Computational Simulation and Identification of Catalytic Sites

The binary complex structure of BcUGT009 and UDP‐Glu was predicted using Boltz2, yielding a highly reliable model (pTM = 0.947, ipTM = 0.982, confidence score = 0.927, average pLLDDT = 0.914). Following assessment of protonation states (H32 as HID, D135 deprotonated), Iristectorigenin B was docked into the substrate pocket using AutoDock Vina. The resulting near‐attack conformation was selected and refined with MOE for further analysis.

To experimentally verify predicted sites, site‐directed mutagenesis was conducted. A UDP‐Glu overproduction platform was engineered in *S. cerevisiae* BY4742 by overexpressing *PGM1*, *PGM2*, and *UGP1* (Liu et al. 2021). Mutations were introduced into the wild‐type Y33‐BcUGT009 plasmid (primers in Table S28), and confirmed mutants were transformed into the engineered strain via the lithium acetate method. After 5‐day fermentation in 24‐deepwell plates (with 0.1 mM substrate added on Day 2), mutant activities were evaluated by comparing HPLC peak areas to the wild‐type enzyme.

## Author Contributions

S.‐C.Y., M.‐J.Q., G.‐H.Z., and Y.‐C.Z. designed the project. Y.‐Y.W., B.‐H.C., and S.‐Y.Y. prepared materials and performed the experiments. G.‐S.X. and Y.‐Y.W. performed the bioinformatics analysis and analysed the data. Y.‐Y.W. wrote the manuscript. X.‐B.L., Y.‐N.W., and R.Y. made constructive comments on the work and manuscript. S.‐C.Y., M.‐J.Q., G.‐H.Z., and Y.‐C.Z. revised the manuscript. All authors read and approved this manuscript.

## Conflicts of Interest

The authors declare no conflicts of interest.

## Supporting information


**Data S1:** pbi70612‐sup‐0001‐DataS1.pdf.

## Data Availability

The original genome sequence data of this study have been deposited in the NCBI BioProjects database and can be accessed through the accession code PRJNA1130555.
