# Supplementary material for: A Near Telomere‐to‐Telomere Genome of Belamcanda chinensis Provides Insights Into Genome Evolution and the Biosynthesis of Characteristic Isoflavones
Source: Plant Biotechnol J. 2026 Mar 14;24(6):4285–301. doi: 10.1111/pbi.70612 (PMC13205724; doi:10.1111/pbi.70612)
Supplement: Supplementary file 1 — Data S1: pbi70612‐sup‐0001‐DataS1.pdf. [file PBI-24-4285-s001.pdf]

Supplementary Materials for

A near telomere-to-telomere genome of *Belamcanda chinensis* provides insights into genome evolution and the biosynthesis of characteristic isoflavones

Yuan-Yuan Wang [1,2†], Bi-Huan Chen [1,2†], Gui-Sheng Xiang [1,2], Yi-Na Wang [1,2], Run Yang [1,2], Xiao-Bo Li [1,2], Shi-Yan Yuan [1,2], Yu-Cheng Zhao [3], Guang-Hui Zhang [1,2*], Min-Jian Qin [3*], Sheng-Chao Yang [1,2,4*]

[1] College of Agronomy and Biotechnology, National-Local Joint Engineering Research Center on Gemplasm Innovation & Utilization of Chinese Medicinal Materials in Southwest, The Key Laboratory of Medicinal Plant Biology of Yunnan Province, Yunnan Agricultural University, Kunming, Yunnan 650201, China.

[2] Yunnan Characteristic Plant Extraction Laboratory, Kunming, Yunnan 650106, China.

[3] Department of Resources Science of Traditional Chinese Medicines, School of Traditional Chinese Pharmacy, and State Key Laboratory of Natural Medicines, China Pharmaceutical University, Nanjing 210009, China.

[4] Yunnan Province Key Laboratory of Cross-Border Chinese Herbal Materials, Honghe University, Mengzi, Yunnan 661199, China.

[†] These authors contributed equally to this work

* Correspondence: Guang-Hui Zhang (zgh73107310@163.com); Min-Jian Qin (minjianqin@163.com); Sheng-Chao Yang (shengchaoyang@163.com)

SUPPLEMENTARY METHODS

Biochemical regents and standard

RNA was extracted from *B. chinensis* roots and rhizomes using the HiPure HP Plant RNA Mini Kit RNA of Magen (Guangzhou, China). Reverse transcription was performed with the PrimeScript™ II 1st Strand cDNA Synthesis Kit (TaKaRa, Dalian, China). *B. chinensis* cDNA was used as a template, and the candidate BcOMTs and BcUGTs genes were amplified by polymerase chain reaction (PCR) using Phanta Max Super-Fidelity DNA polymerase (Vazyme Biotech, Nanjing, China). Standards dichotomitin (**1**), irisflorentin (**3**), iristectorigenin B (**4**), iristectorigenin A (**5**), tectorigenin (**6**), irigenin (**7**), iristectorin B (**8**), iristectorin A (**9**), tectoridin (**10**), and iridin (**11**) were purchased from Yunnan Xili Biotechnology Co., Ltd (Kunming, China). S-Adenosyl-L-methionine (SAM) (**12**) and uridine diphosphate glucose (UDP-Glu) (**13**) were purchased from Solarbio Science & Technology Co., Ltd (Beijing, China). The purity of the standard used is ≥98%.

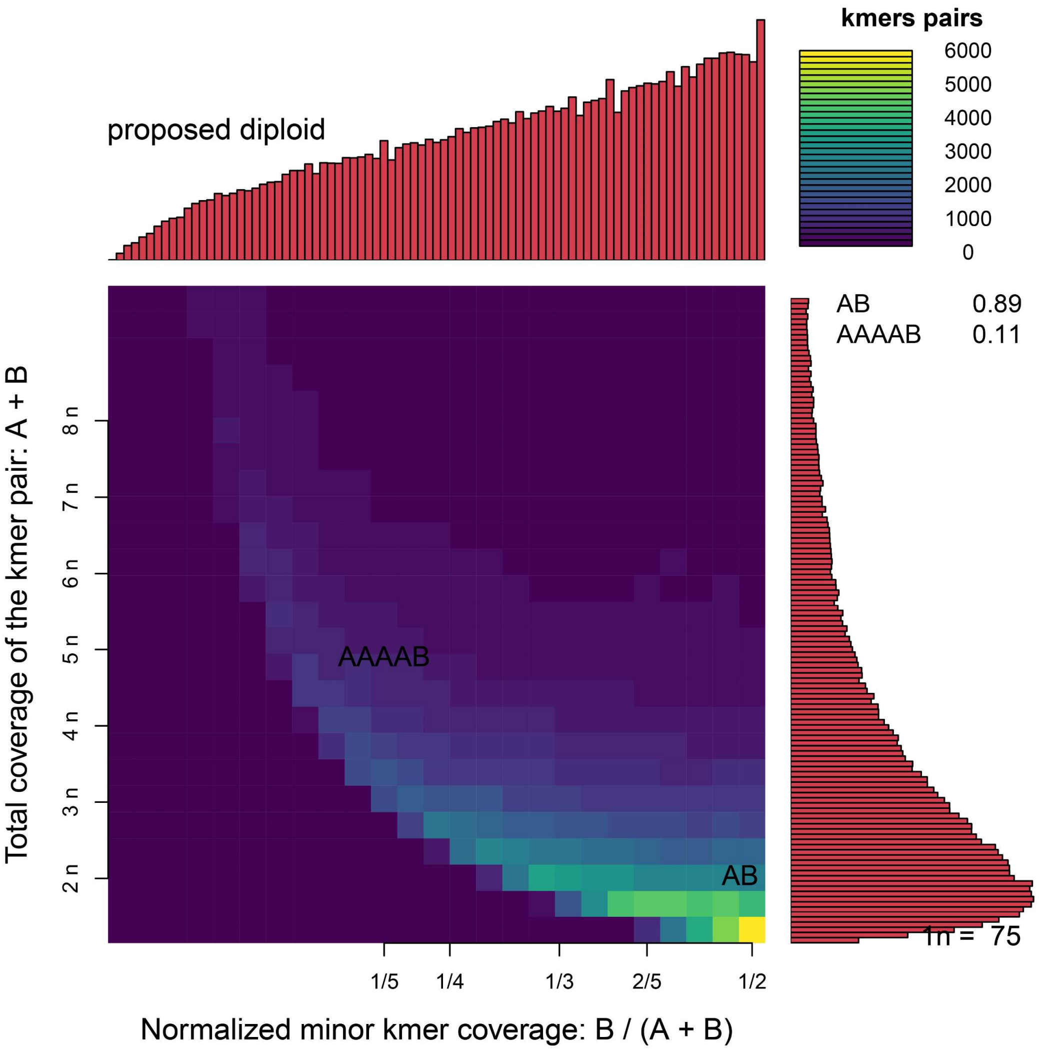

Figure S1 Ploidy analysis using Smudgeplot. Each haplotype structure presents a "smudge" on the map, and the heat of the smudge indicates the frequency of the haplotype structure in the genome.

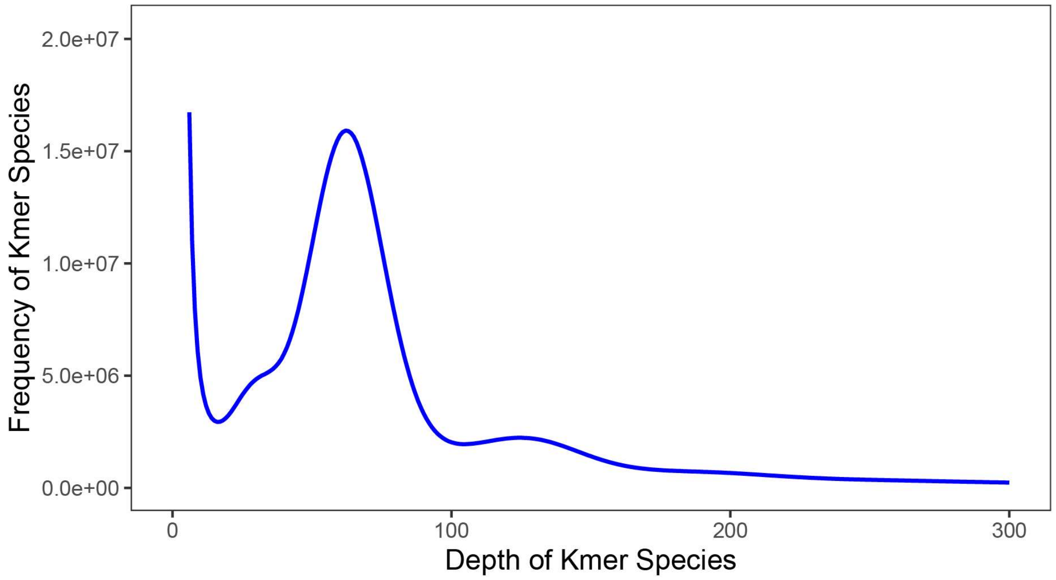

Figure S2 The k-mer (k=17) frequency-depth distribution map of the *B. chinensis* genome shows the depth on the horizontal axis and the frequency of k-mer on the vertical axis.

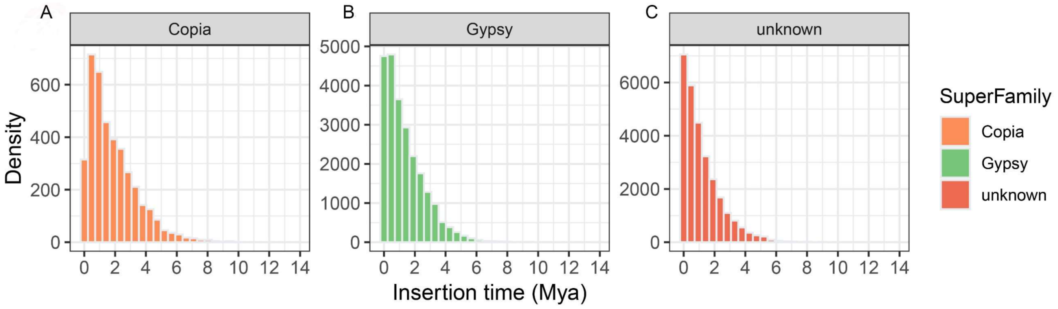

Figure S3 The *Copia* (A), *Gypsy* (B), and unknown (C) LTR-RTs insert time in the *B. chinensis* genome.

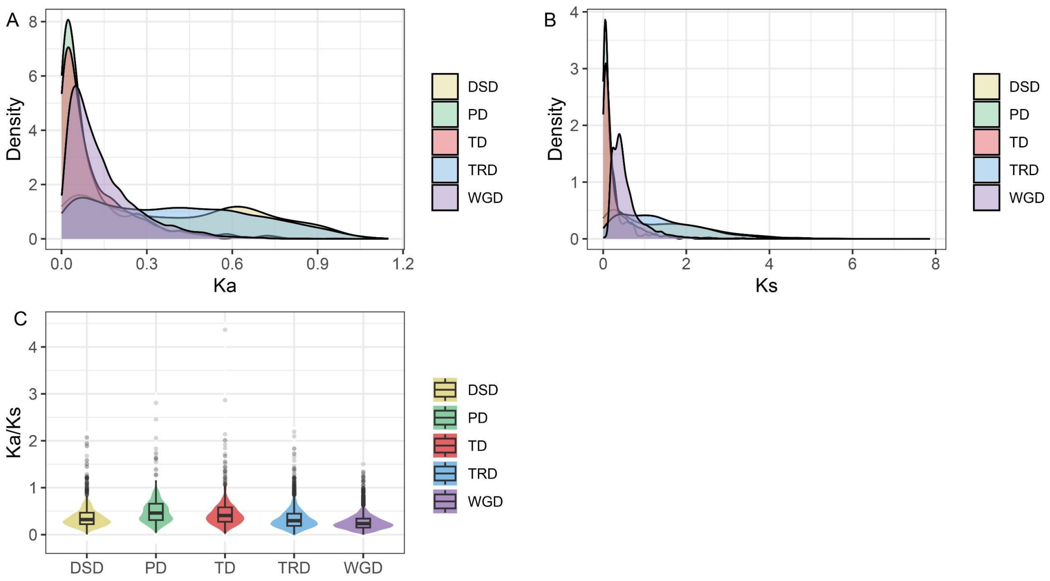

Figure S4 Analysis of Ka and Ks of different gene replication types. Ka (A) and Ks (B) distribution of different types of duplicated genes (DSD, PD, TD, TRD, and WGD). (C) Distributions of Ka/Ks ratio gene pairs from different types of duplicated genes (DSD, PD, TD, TRD, and WGD). The points in the boxplot are outliers, and the center line is the average value.

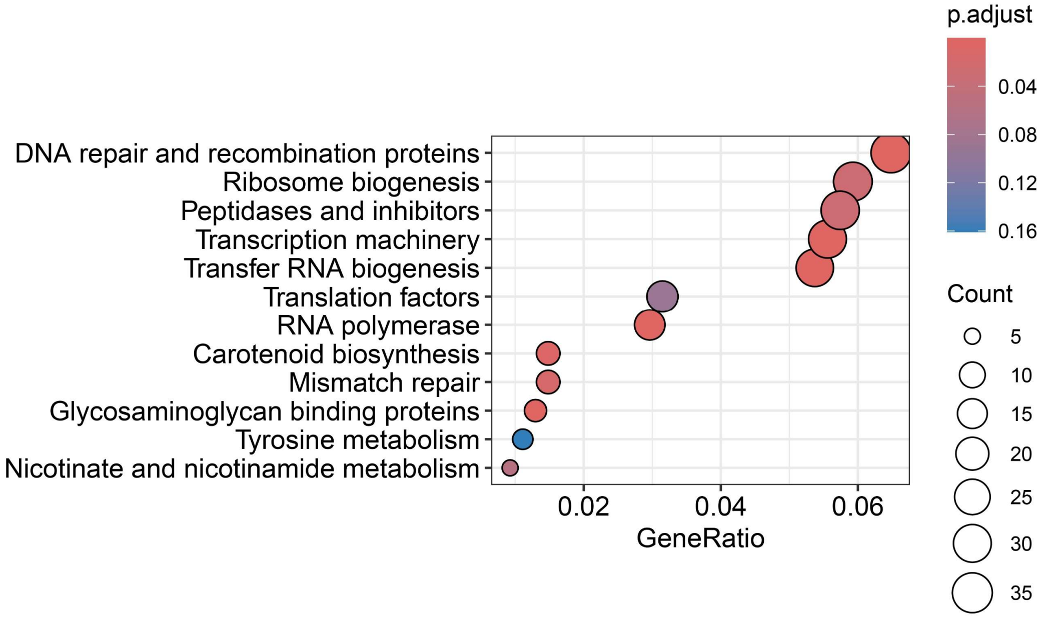

Figure S5 KEGG signaling pathway enrichment analysis of the DSD genes in the *B. chinensis* genome.

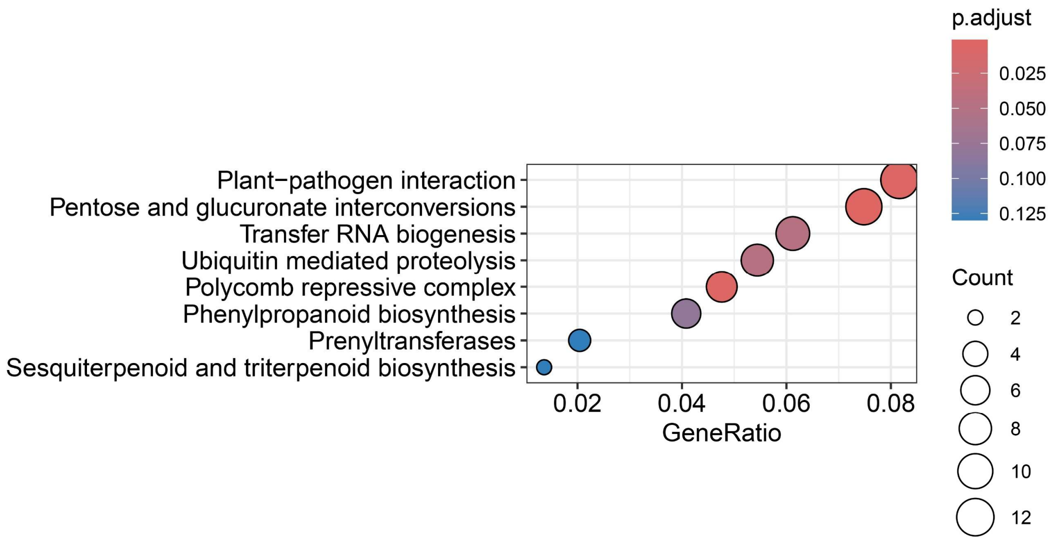

Figure S6 KEGG signaling pathway enrichment analysis for the PD genes in the *B. chinensis* genome.

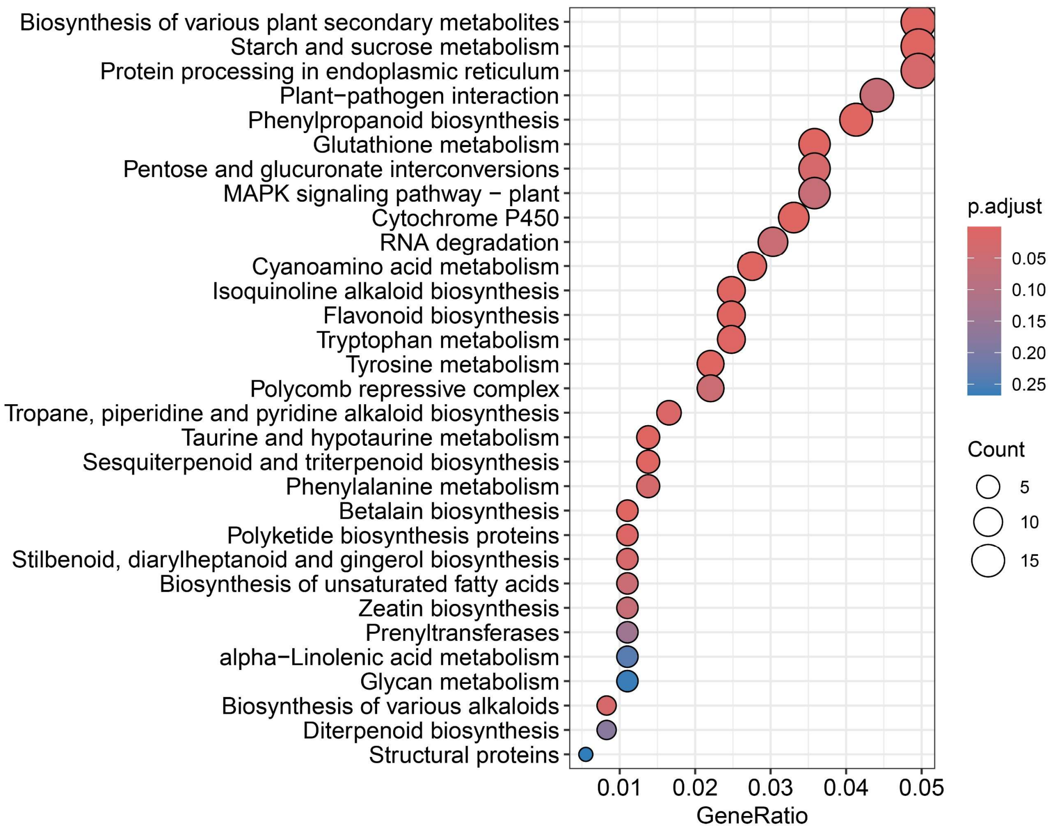

Figure S7 KEGG signaling pathway enrichment analysis of the TD genes in the *B. chinensis* genome.

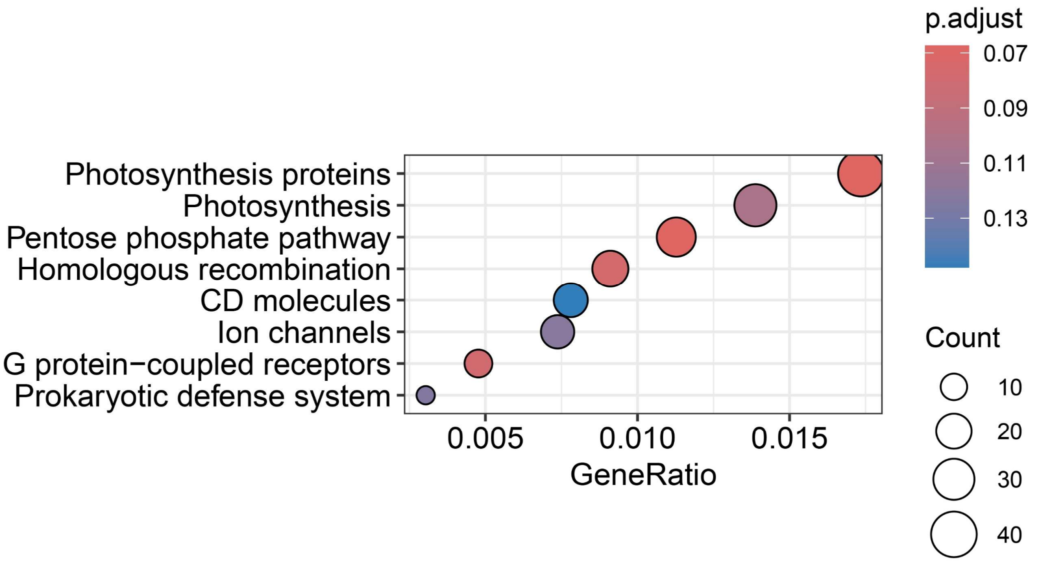

Figure S8 KEGG signaling pathway enrichment analysis of the TRD genes in the *B. chinensis* genome.

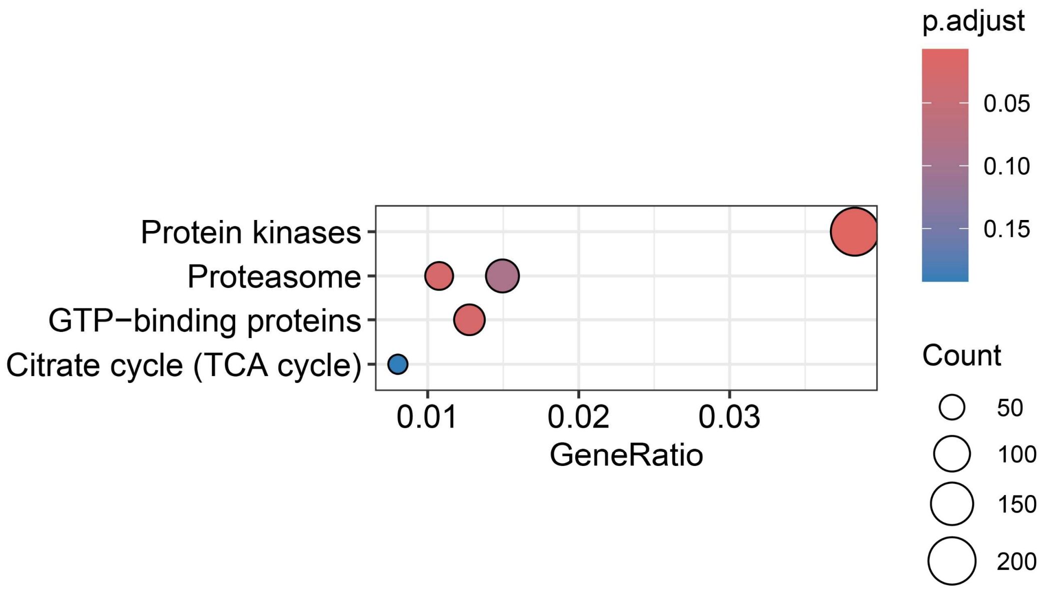

Figure S9 KEGG signaling pathway enrichment analysis of the WGD genes in the *B. chinensis* genome.

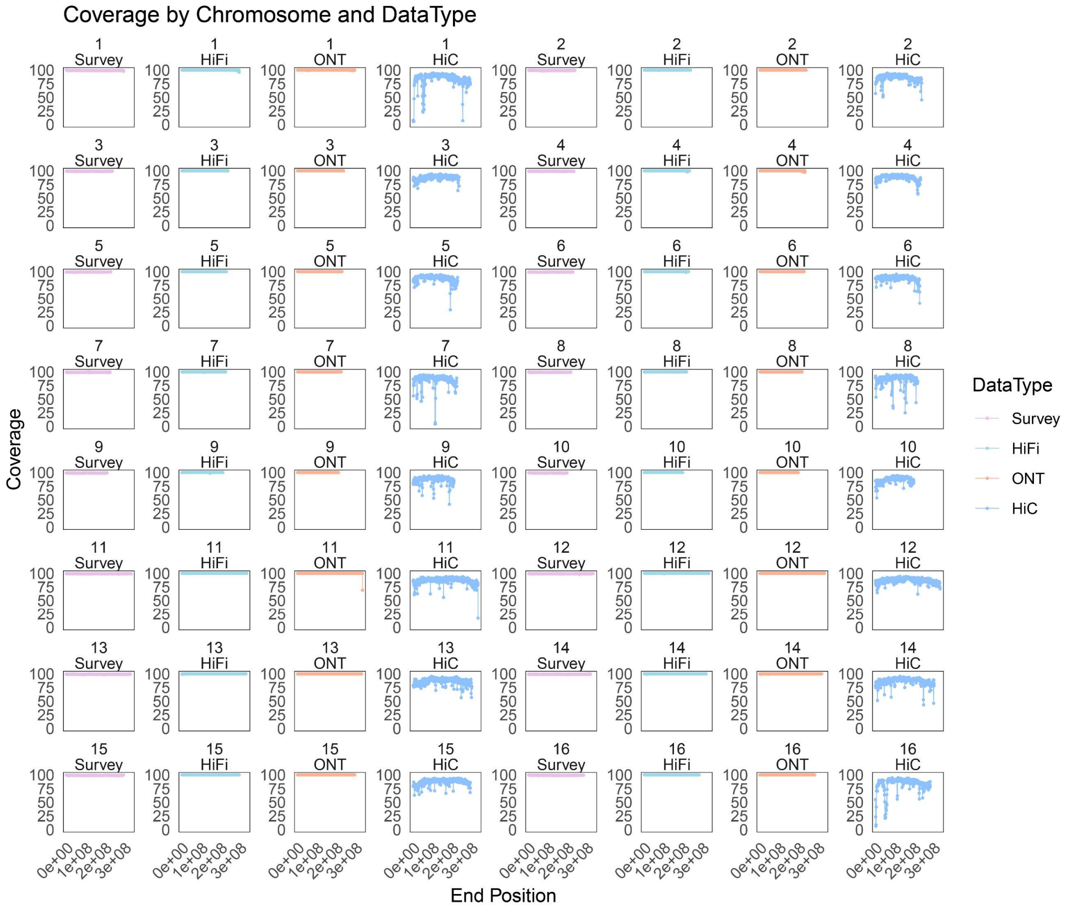

Figure S10 The genomic coverage of Survey, HiFi, ONT, and HiC on each chromosome.

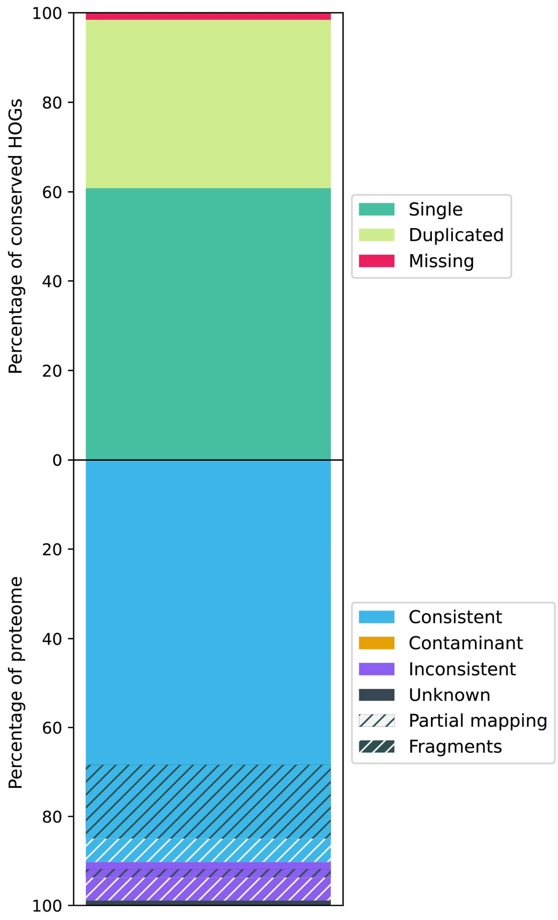

Figure S11 OMArk's visualization result for the *B. chinensis* genome assembly.

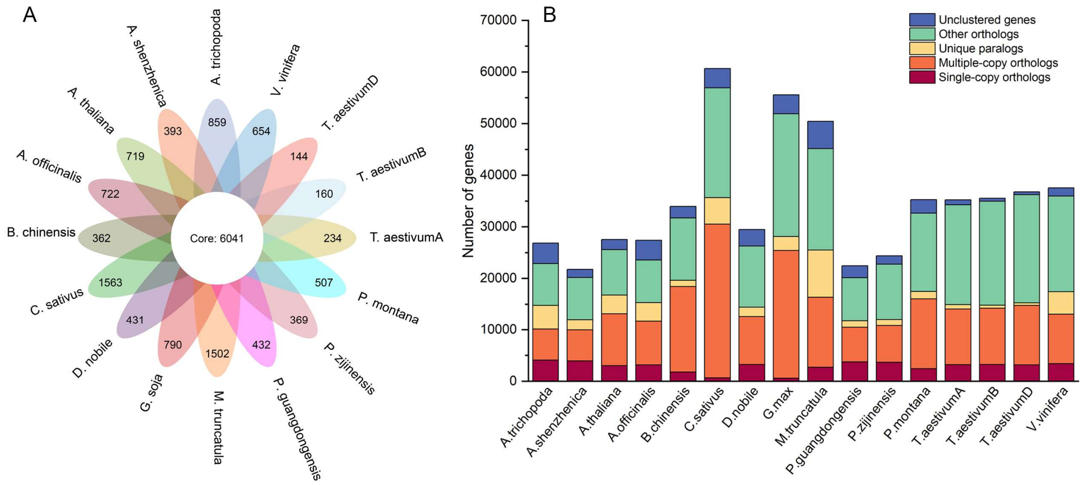

Figure S12 The comparative analysis of genomes of 16 (14+2) species revealed the distribution of core gene families and species-specific genes. (A) The flower plot shows the common orthogroups (center) of 16 species and their species-specific orthogroups. (B) Homologous gene analysis results of 16 different species.

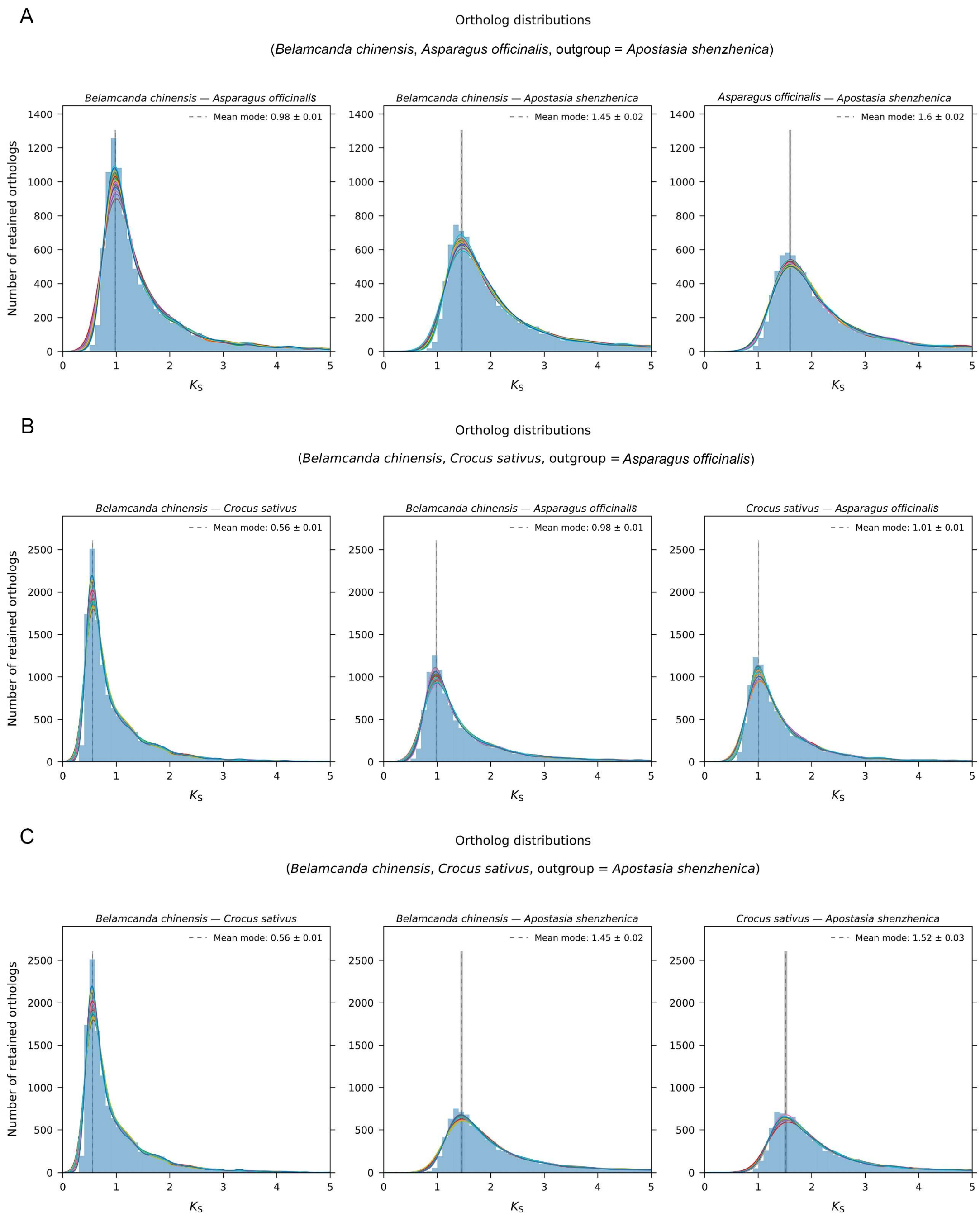

Figure S13 Ks distributions of orthologous gene pairs used for branch-specific rate calibration. (A) *B. chinensis-Asparagus officinalis-Apostasia shenzhenica*; (B) *B. chinensis-Crocus sativus-A. officinalis*; (C) *B. chinensis-C. sativus-A. shenzhenica*. Dashed gray vertical lines indicate the mean-mode Ks estimates. Light blue histograms represent the frequency distribution of orthologous Ks values, with the first 20 bootstrapped kernel density estimates (KDEs) overlaid as colored curves.

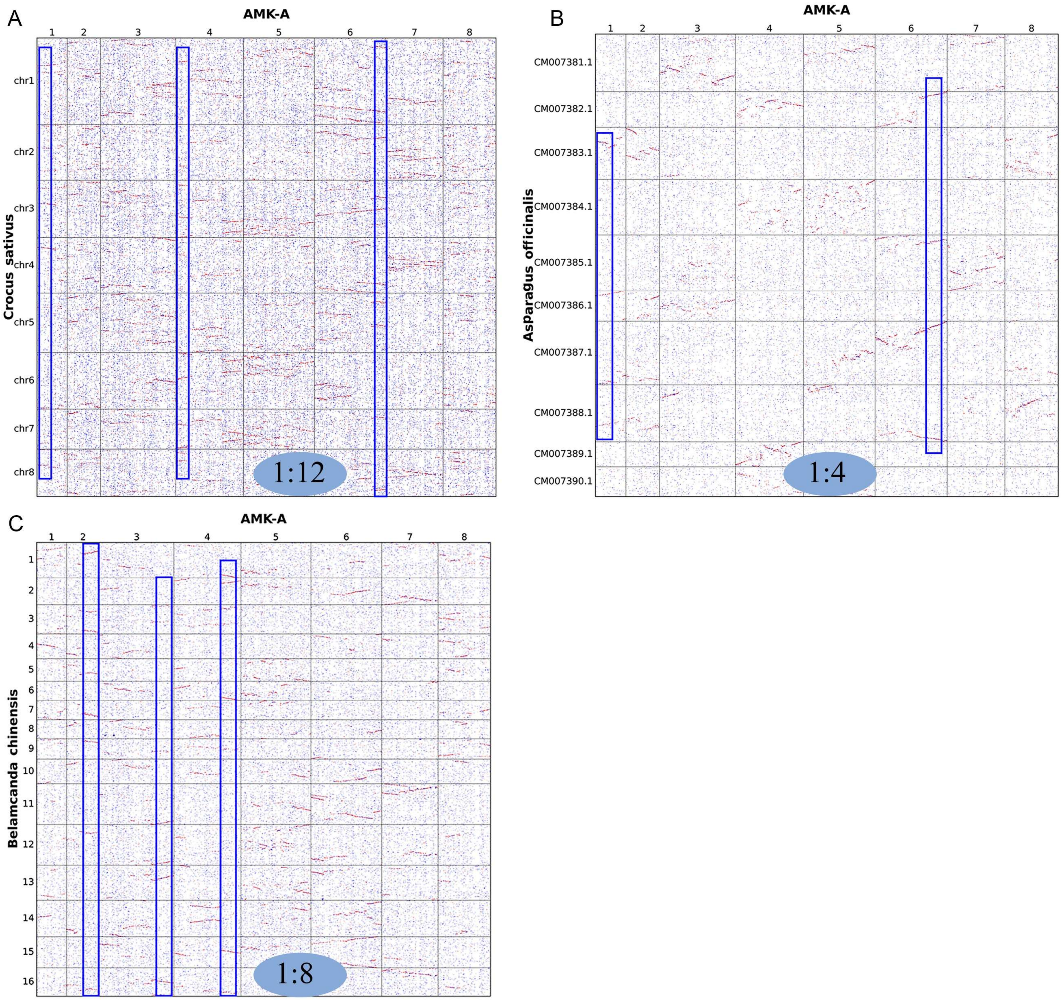

Figure S14 WGDI visualization of dot plots collinearity between *C. sativus* (WGD+WGD+WGT) (A), *A. officinalis* (WGD+WGD) (B), *B. chinensis* (WGD+WGD+WGD) (C), and AMK-A.

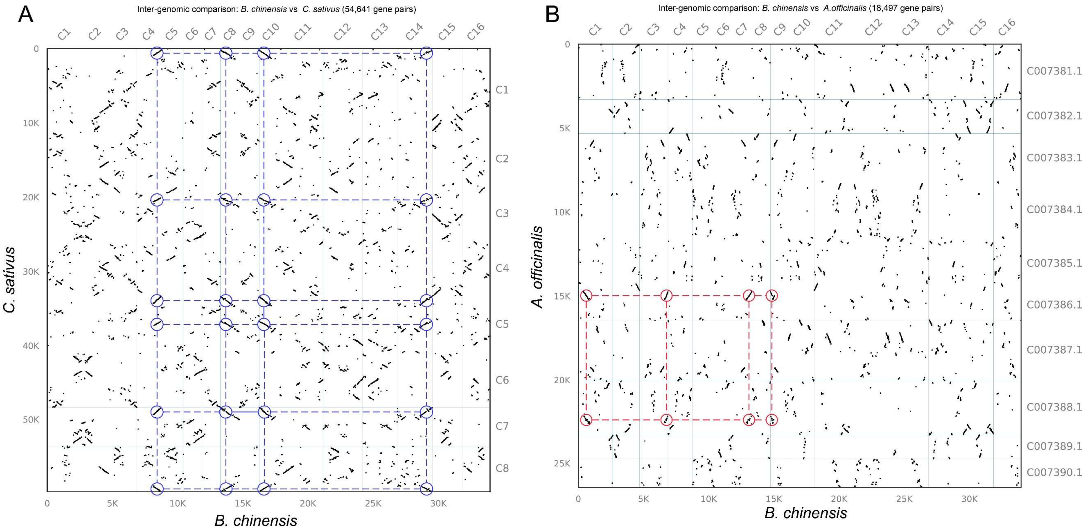

Figure S15 JCVI visualization of synteny blocks and synteny pattern between *C. sativus* and *B. chinensis* (A), *A. officinalis* and *B. chinensis* (B).

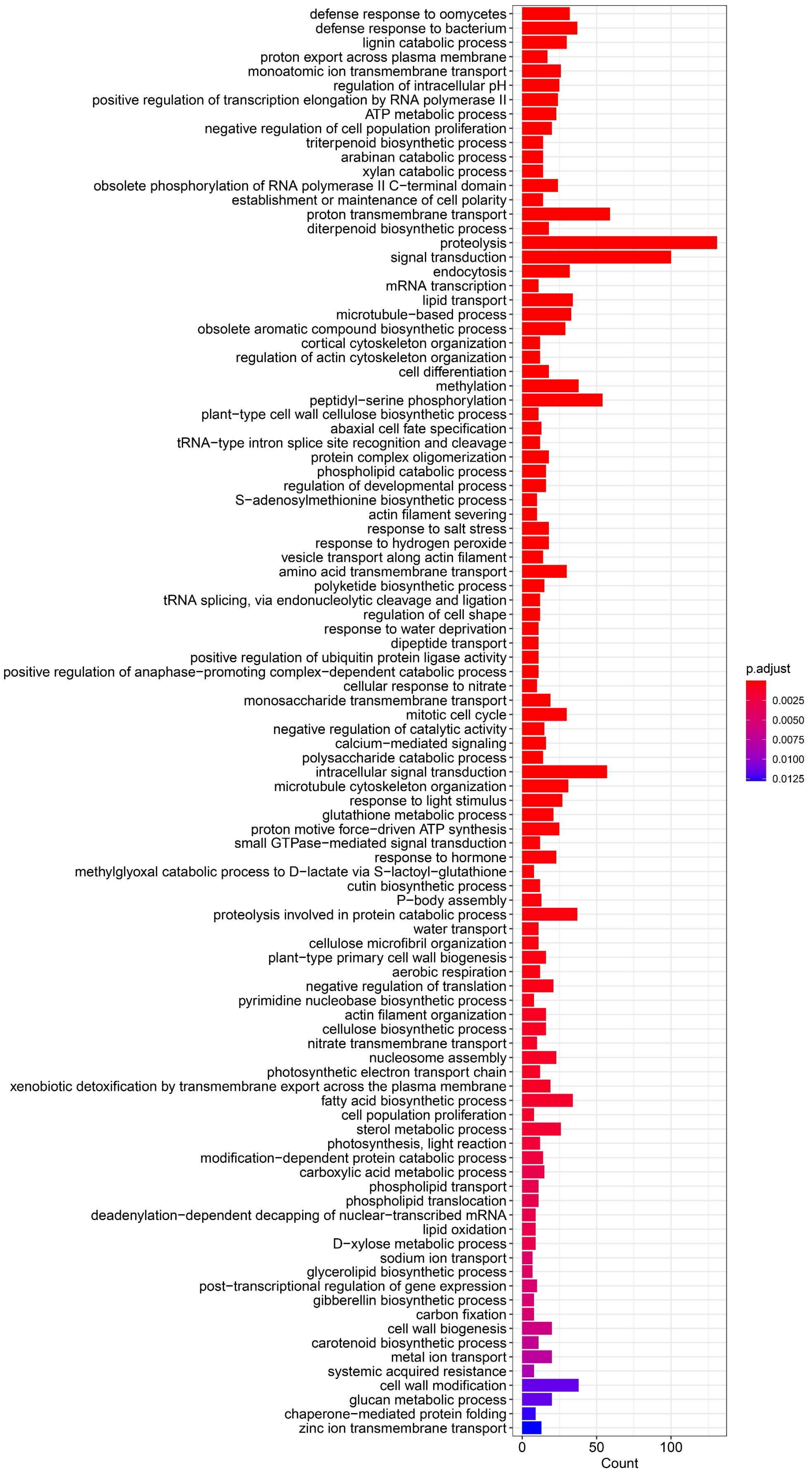

Figure S16 GO enrichment for the biological process (BP) category of significantly expanded gene families in *B. chinensis*.

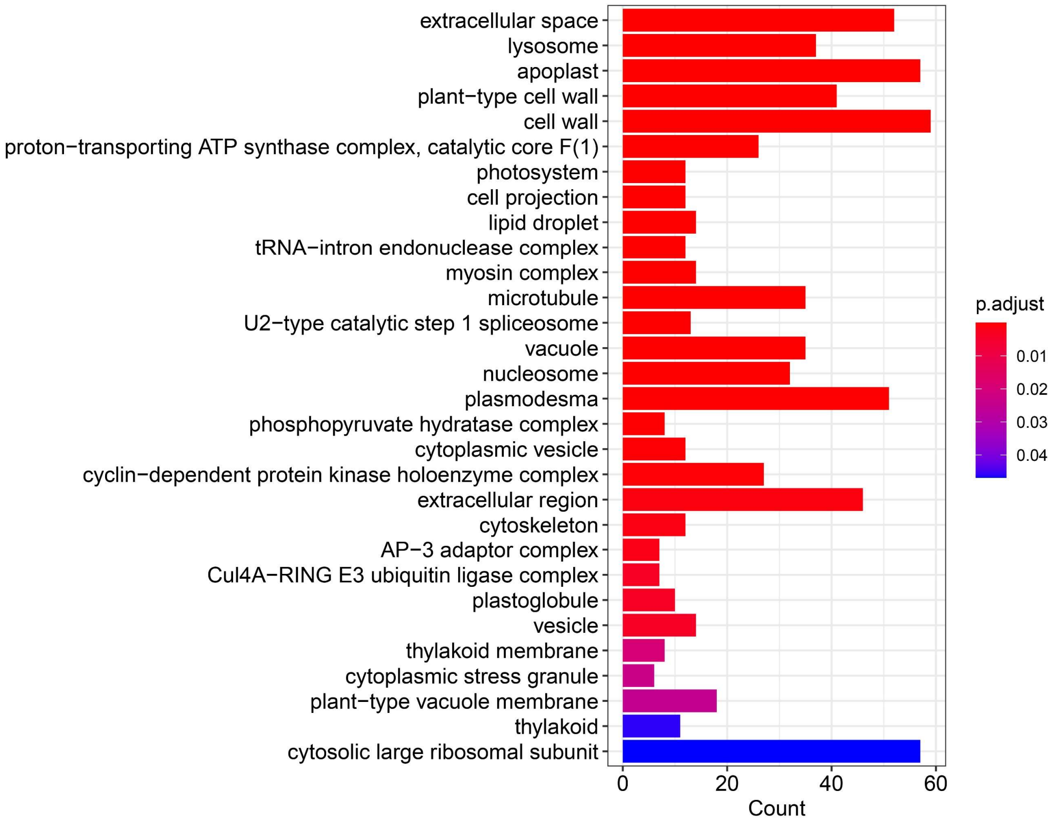

Figure S17 GO enrichment for the cellular component (CC) category of significantly expanded gene families in *B. chinensis*.

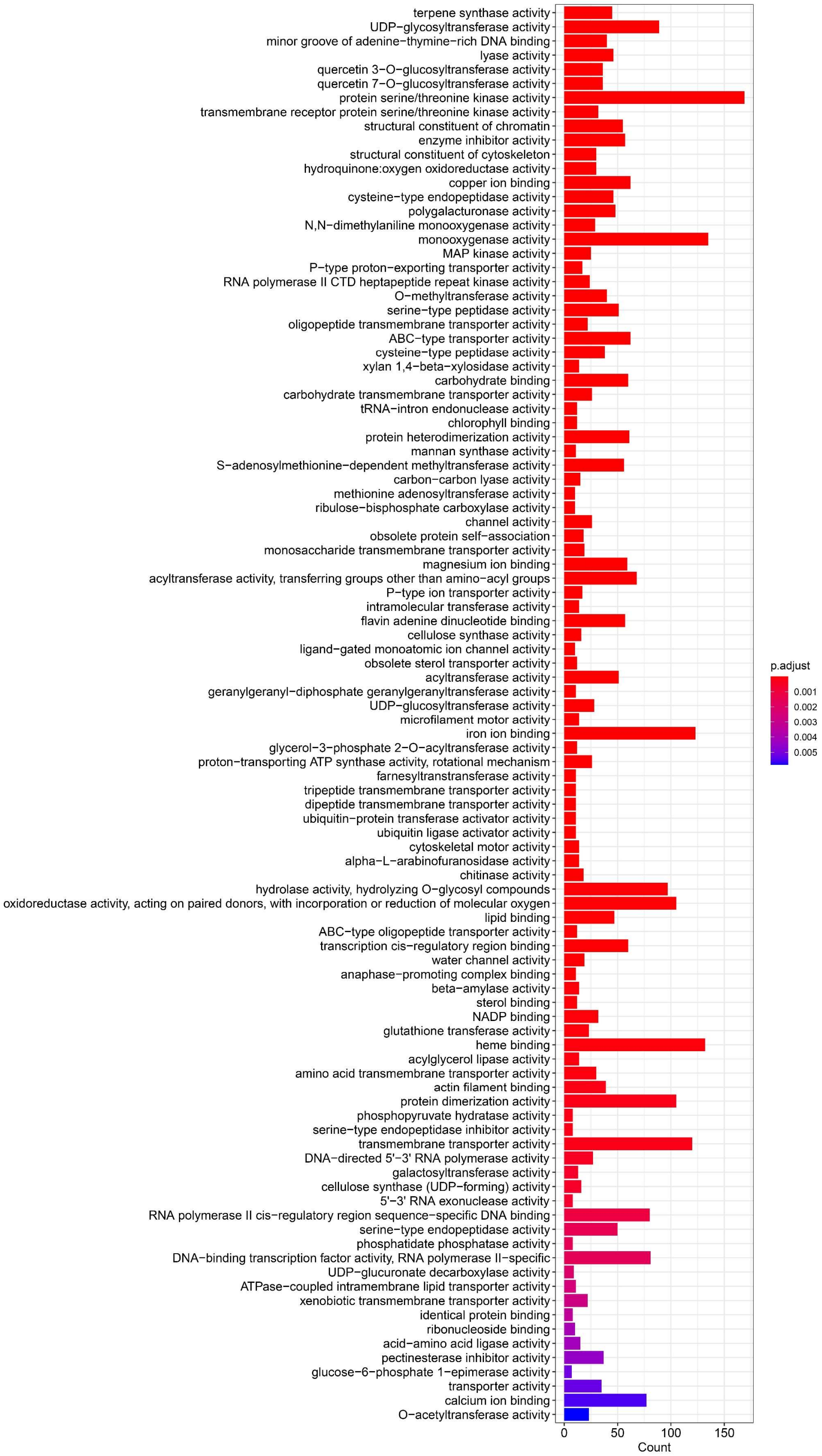

Figure S18 GO enrichment for the molecular function (MF) category of significantly expanded gene families in *B. chinensis*.

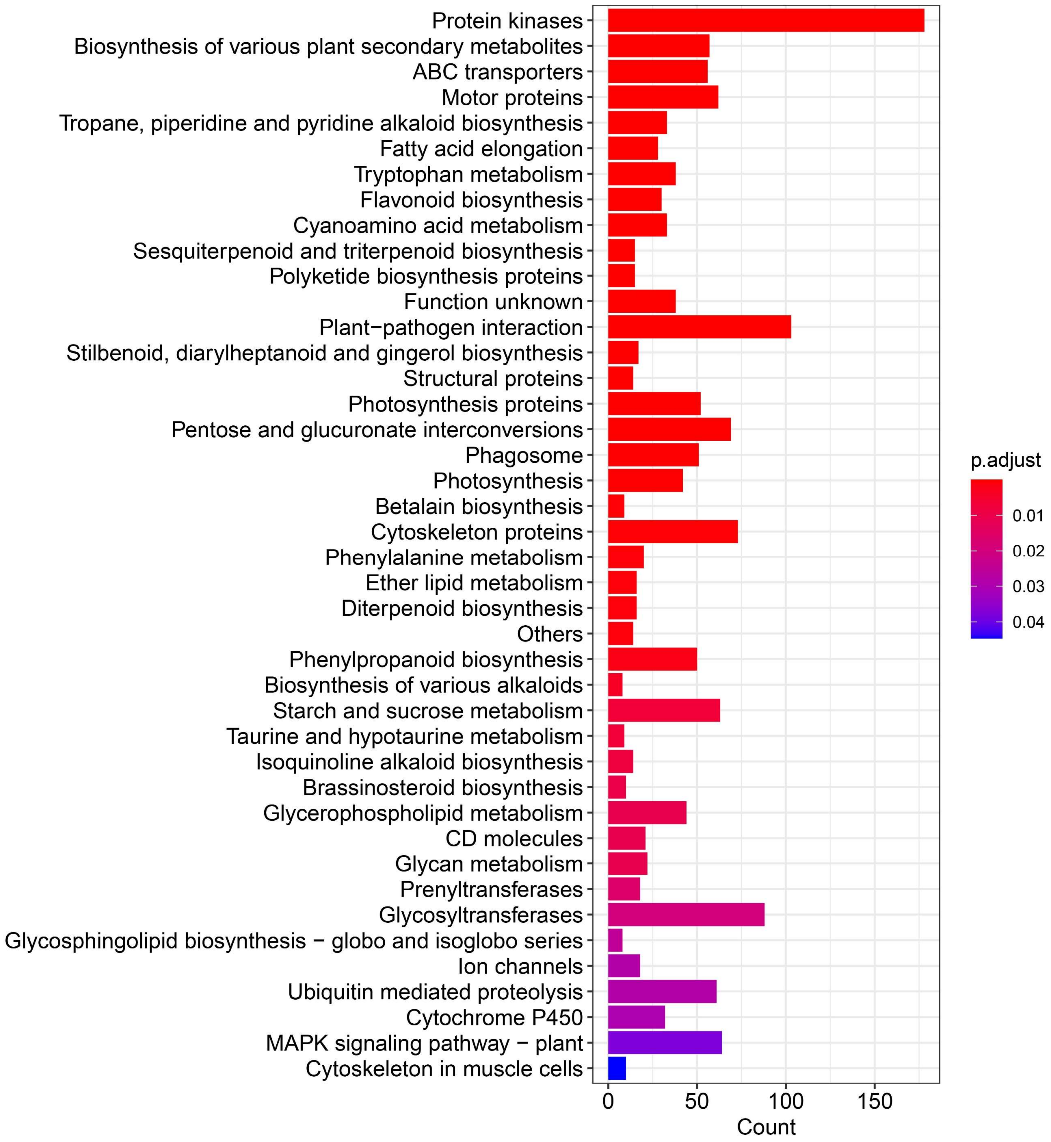

Figure S19 KEGG enrichment of significantly expanded gene families in *B. chinensis*.

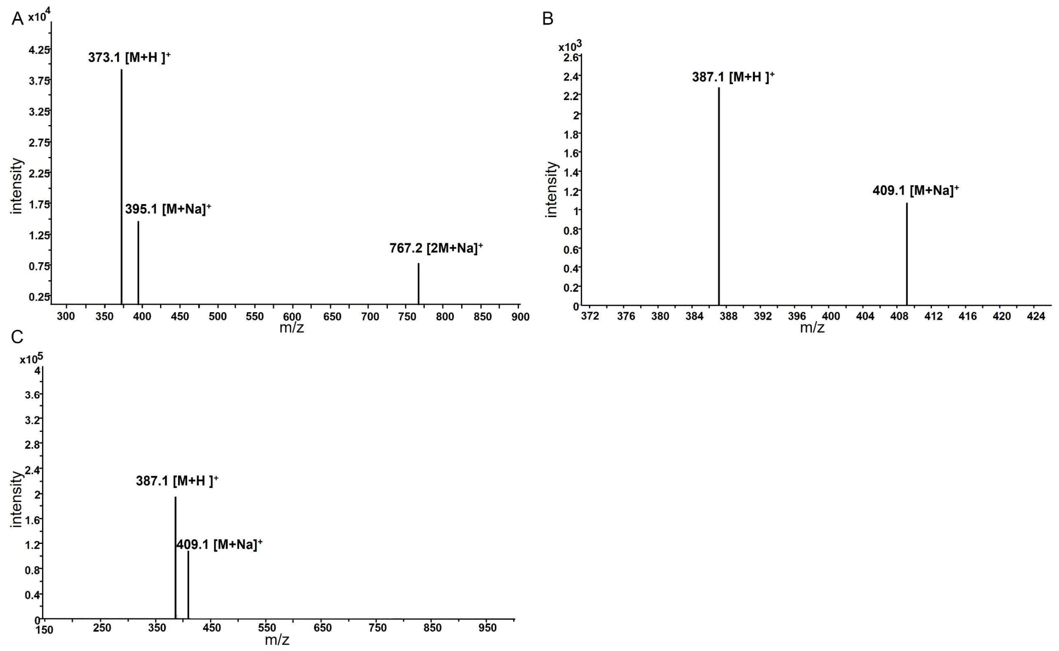

Figure S20 LC\MS analysis of 3'-hydroxy-5,4',5'-trimethoxy-6,7-methylenedioxyisoflavone (2) (A), irisflorentin (3) in the sample (B), and standard irisflorentin (3) (C).

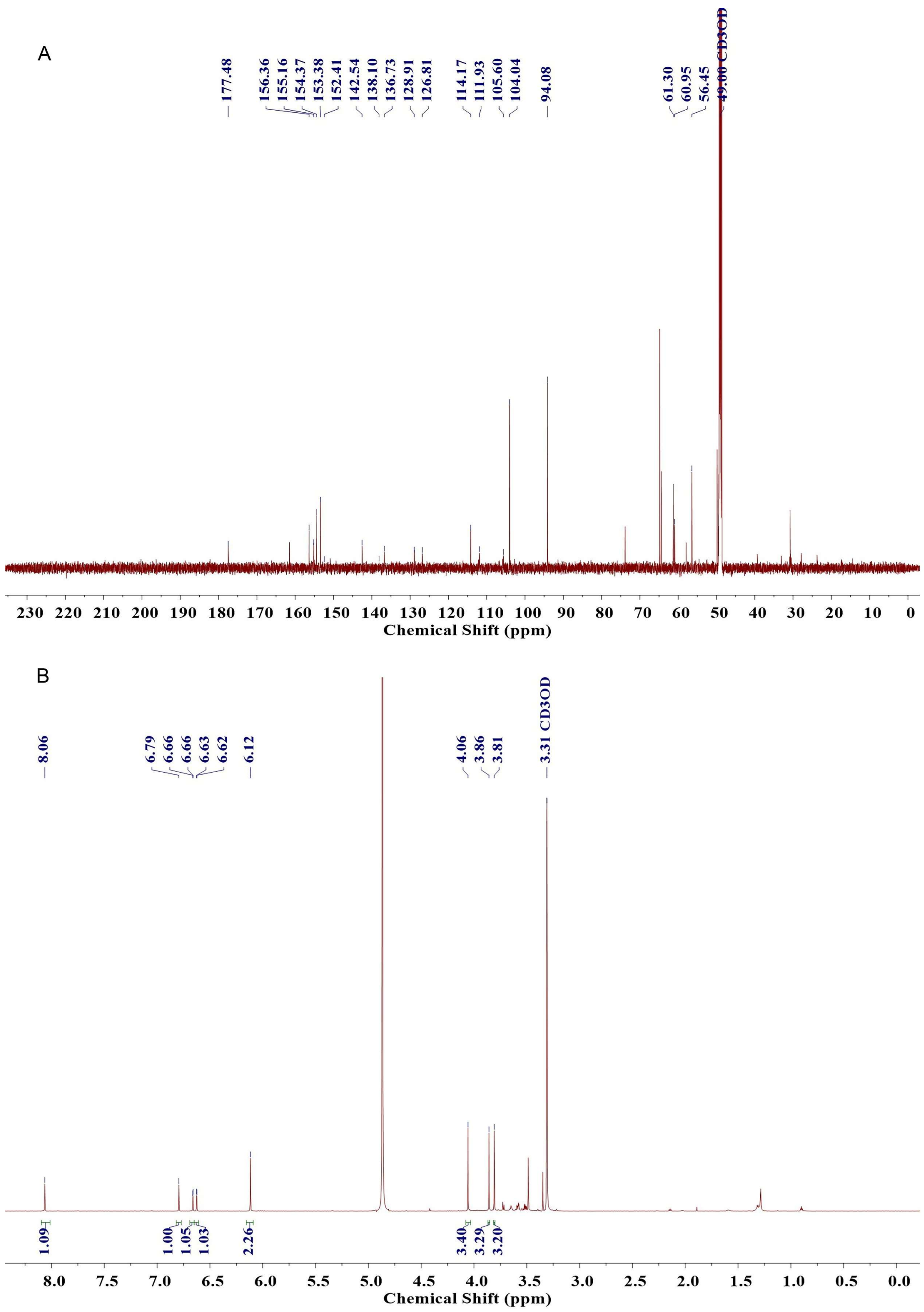

Figure S21 The nuclear magnetic resonance (NMR) spectrum of compound **2**. (A) ^{13}C NMR spectrum of compound **2** (200 MHz, MeOD). (B) ^{1}H NMR spectrum of compound **2** (800 MHz, MeOD).

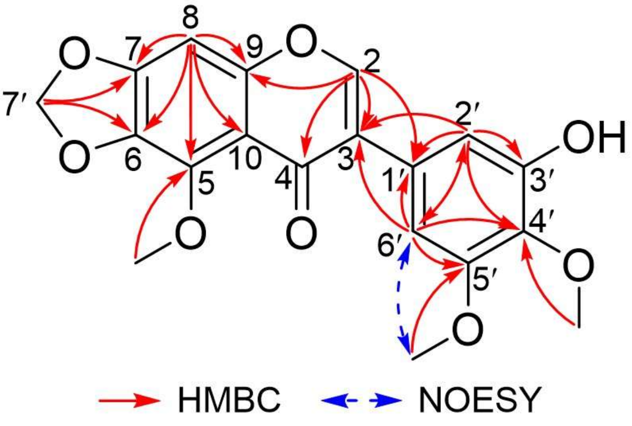

Figure S22 HMBC and NOESY correlation signal diagram of compound **2**.

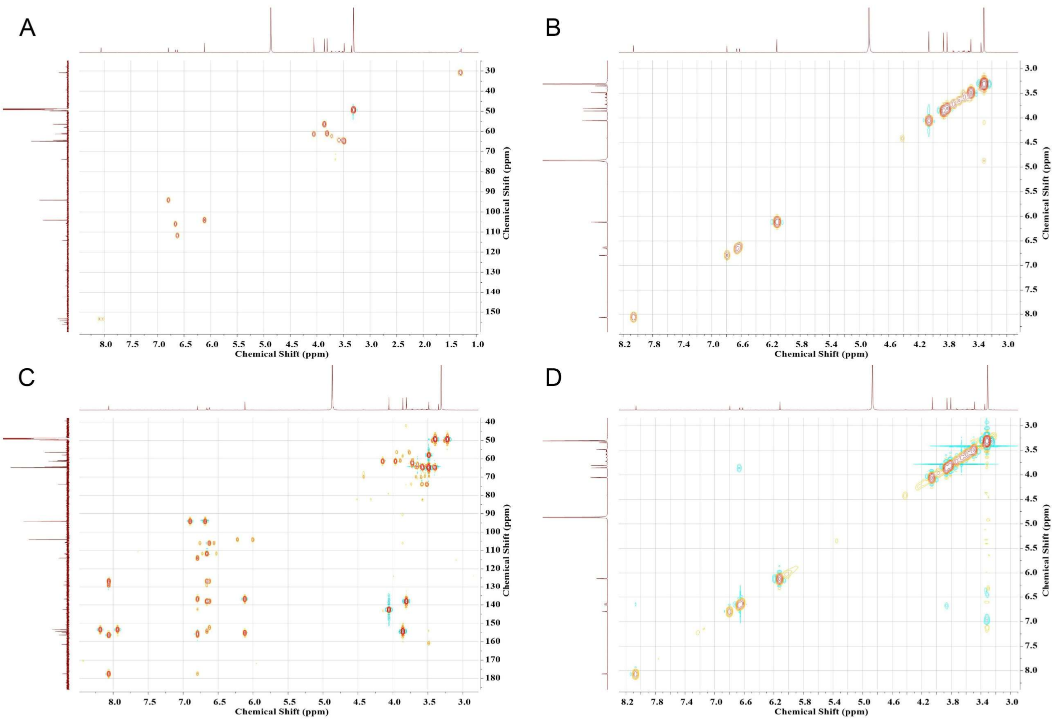

Figure S23 Two-dimensional nuclear magnetic resonance (2D NMR) spectrum of compound **2** included HSQC (A), COSY(B), HMBC (C), and NOESY (D).

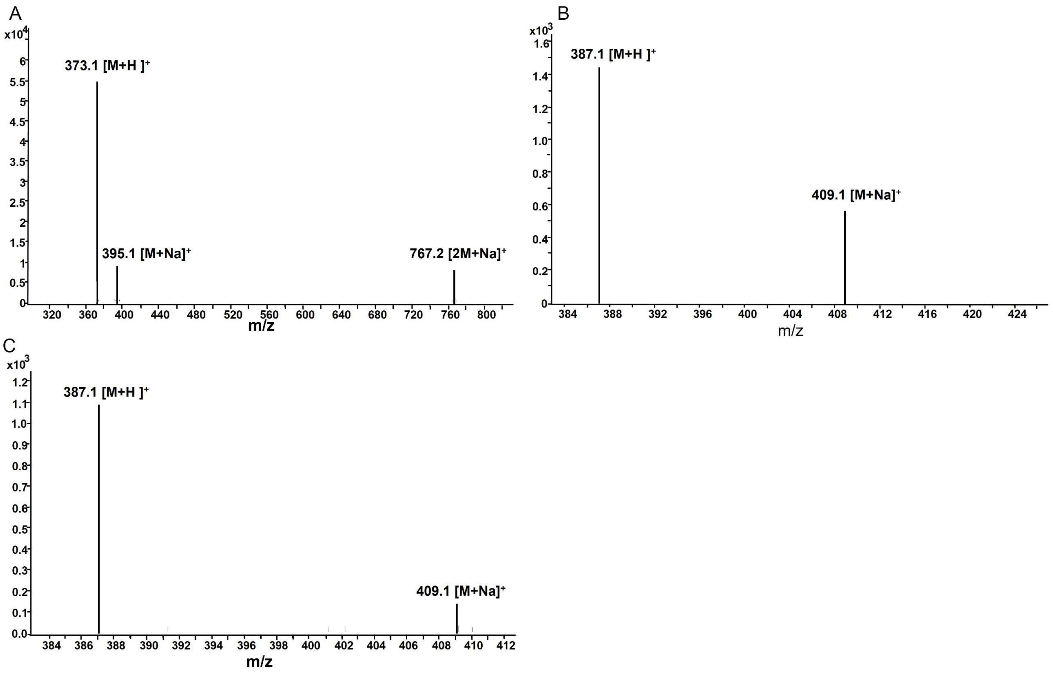

Figure S24 LC/MS extracted ion chromatograms of compounds from *Nicotiana benthamiana* leaves transiently expressing different BcOMTs: (A) compound 2 from BcOMT33-expressing leaves, (B) iristectorin from BcOMT33-expressing leaves, and (C) iristectorin from BcOMT03-expressing leaves.

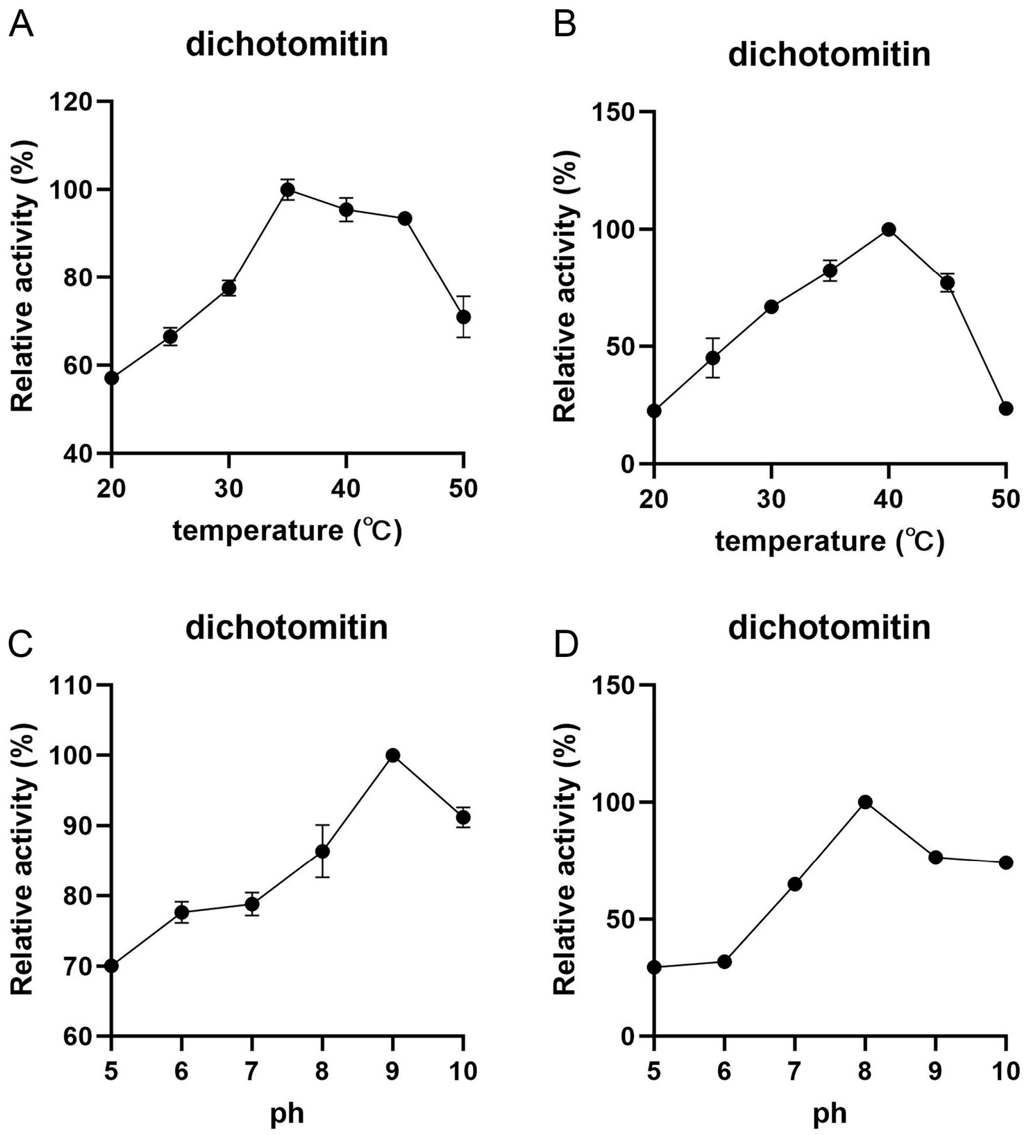

Figure S25 Optimal temperature (A, B) and pH (C, D) profiles for BcOMT3 (A, C) and BcOMT33 (B, D) enzyme activities. Values are presented as mean ± SD from three independent biological replicates.

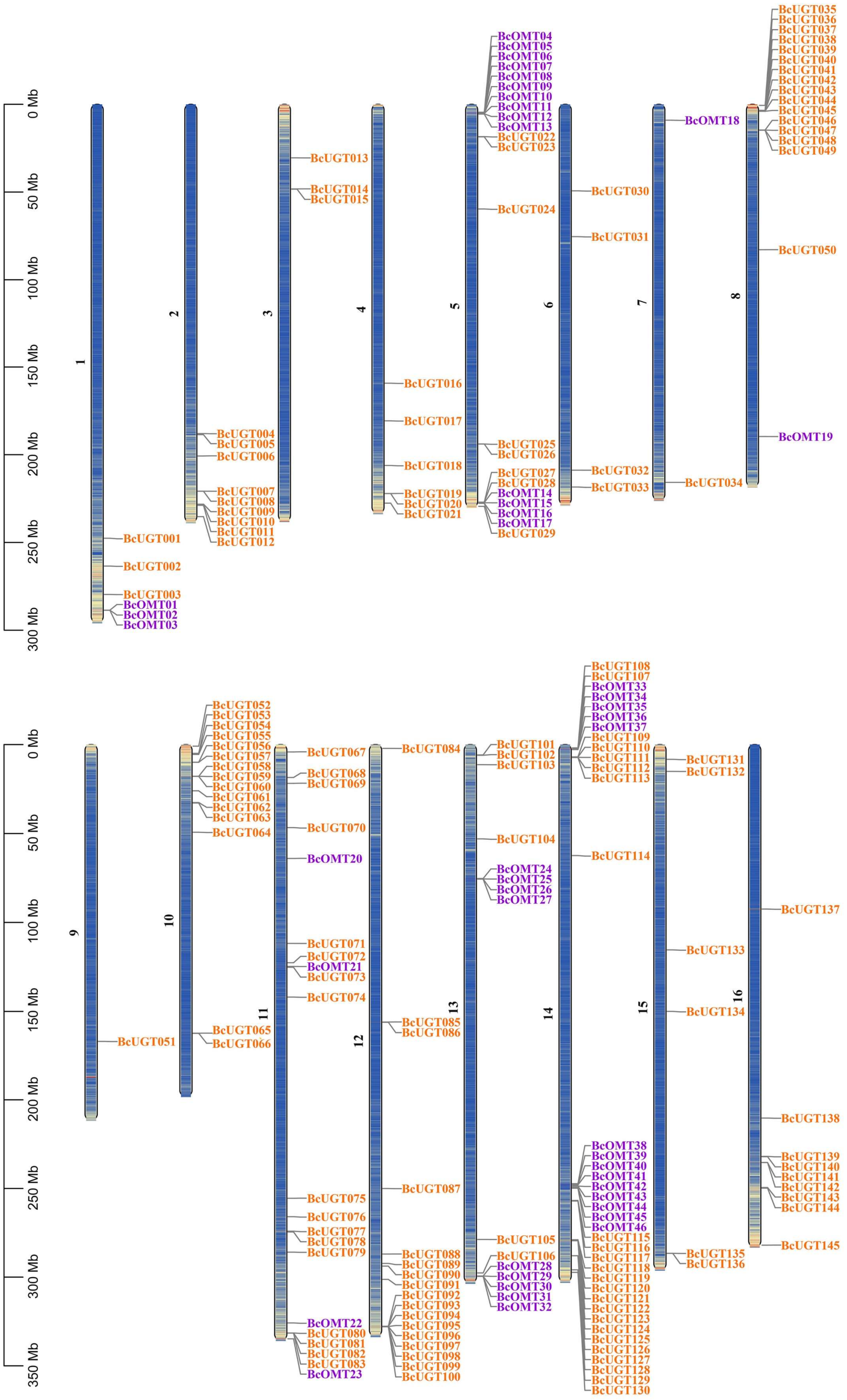

Figure S26 46 BcOMTs and 145 BcUGTs are located on the 16 chromosomes. The purple font represents BcOMTs, and the orange font represents BcUGTs.

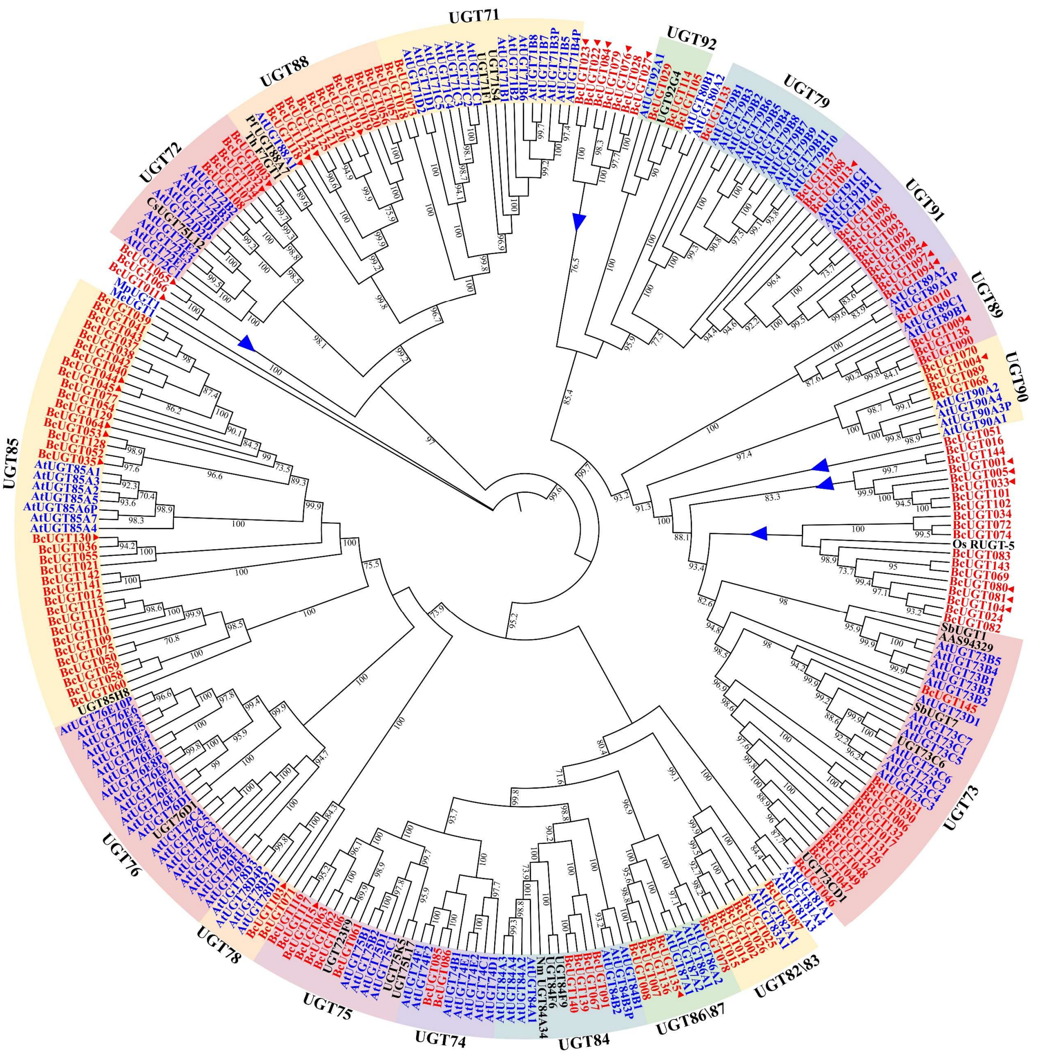

Figure S27 Phylogeny analysis of BcUGTs. The tree illustrates 19 phylogenetic subfamilies of UGTs, with UGTs from *A. thaliana* indicated by the prefix "At." in blue, UGTs with known functions in other species marked in black, and UGTs from *B. chinensis* highlighted in red. Branches with blue triangles represent the unique branches of *B. chinensis* relative to *A. thaliana*. Candidate BcUGTs genes are represented by red triangles.

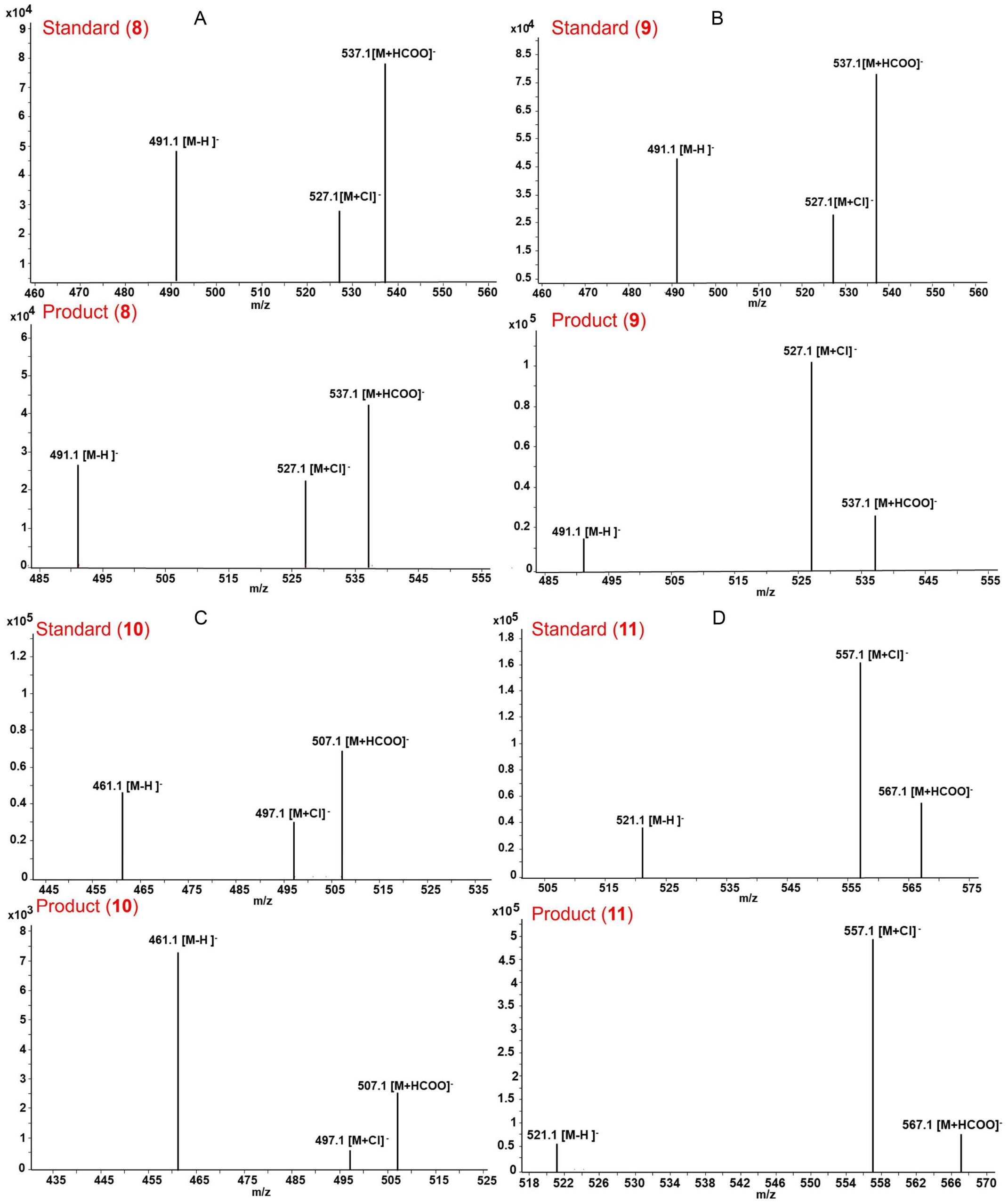

Figure S28 LC\MS analysis of compounds **8** (A), compounds **9** (B), compounds **10** (C), and compounds **11** (D) in standards and samples.

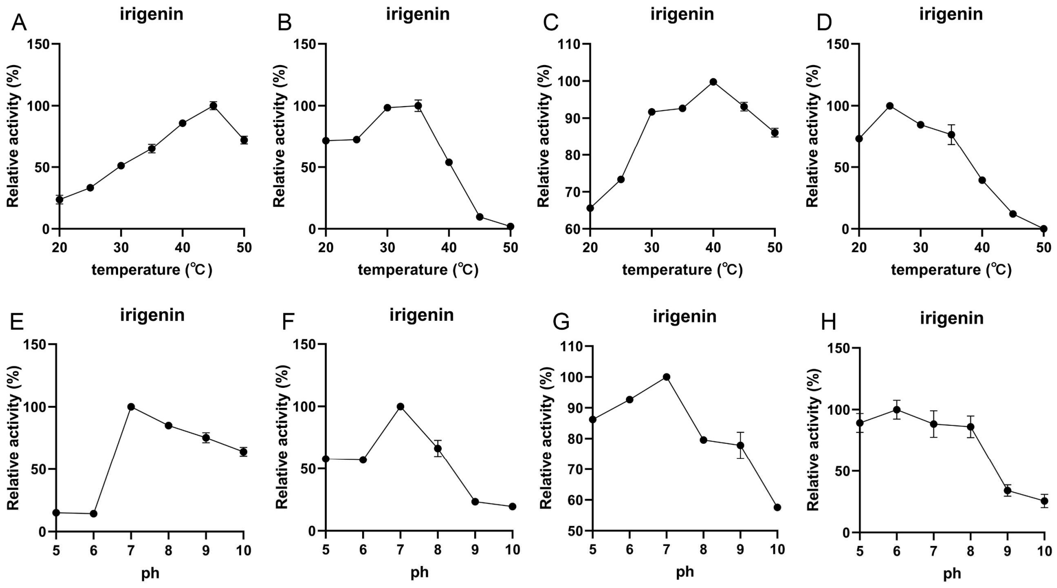

Figure S29 Optimal temperature (A-D) and pH (E-H) profiles for BcUGT009 (A, E), BcUGT032 (B, F), BcUGT119 (C, G), and BcUGT124 (D, H) enzyme activities. Values are presented as mean ± SD from three independent biological replicates.

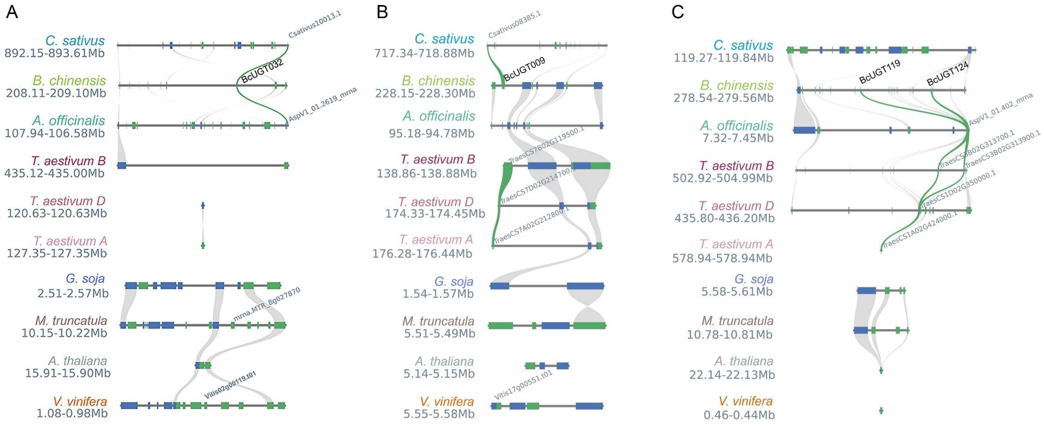

Figure S30 Microsynteny analysis of BcUGT032 (A), BcUGT009 (B), BcUGT119, and BcUGT124 (C) among *C. sativus*, *B. chinensis*, *A. officinalis*, *T. aestivum*, *G. soja*, *M. truncatula*, *A. trichopoda*, and *V. vinifera*. The syntenic BcUGTs are highlighted in blue. The genomic region is indicated under the species name.

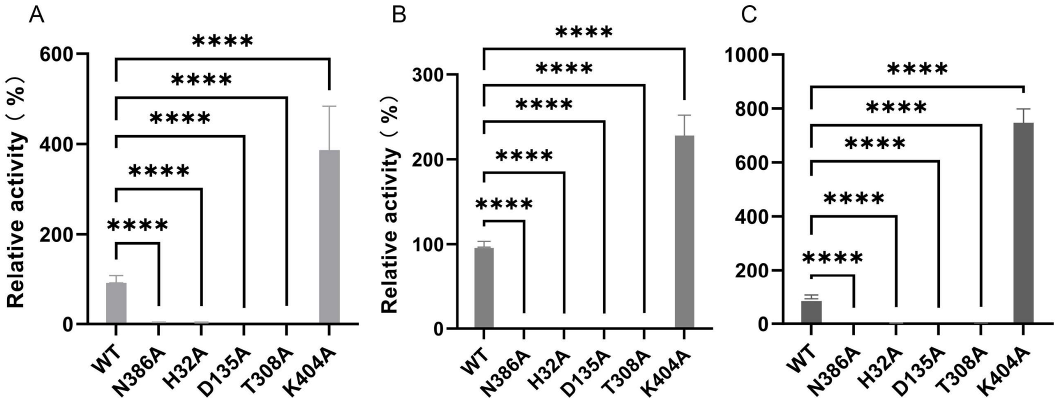

Figure S31 Changes in the relative catalytic activities of wild-type BcUGT009 and its mutants after adding the same dose of iristectorigenin A (A), tectorigenin (B), and irigenin (C). Statistical significance was determined by one-way ANOVA with Dunnett's multiple-comparison correction against the wild type as a common control. Data are presented as mean ± 95% CI of three independent biological replicates (n=3), with asterisks indicating ****P< 0.0001.

Table S1 Statistics of contigs results.

Type	Contig length (Mb)	Contig number
N90	66,287,037	21
N80	118,513,538	16
N70	154,912,975	13
N60	171,987,266	11
N50	209,613,489	8
N40	234,015,333	6
N30	238,632,635	5
N20	296,580,323	3
N10	334,140,931	2
Longest	336,376,737	1
Average length	65,491,088	
Total	4,191,429,687	64
Length>=10kb		64
Length>=20kb		64
Length>=50kb		49
Length>=1Gb		35

Table S2 Statistics of final *B. chinensis* genome assembly results.

Type	Contig length (Mb)	Contig number
N60	238,381,040	9
N50	283,435,706	7
N40	296,082,745	6
N30	303,193,782	4
N20	303,725,412	3
N10	333,808,457	2
Longest	336,010,529	1
Average length	261,165,926	
Total	4,178,654,823	16
Length>=10kb		16
Length>=20kb		16
Length>=50kb		16
Length>=1Gb		16

Table S3 Statistics of gap results.

Chromosome	Chromosome length	N_count	Proportion_N
1	296,082,745	200	0
2	239,037,593	200	0
3	238,381,040	0	0
4	233,798,159	0	0
5	230,387,390	0	0
6	229,045,783	0	0
7	226,392,888	200	0
8	218,682,584	0	0
9	211,879,600	200	0
10	198,488,806	0	0
11	336,010,529	0	0
12	333,808,457	0	0
13	303,725,412	0	0
14	303,193,782	0	0
15	296,304,349	0	0
16	283,435,706	0	0

note：200 N counts as a Gap.

Table S4 Statistics of telomeres results in the *B. chinensis* genome.

Chromosome	Chromosome length (bp)	Status	Left number	Left direction	Right number	Right direction
1	296,082,745	right	0		387	-
2	239,037,593	right	0		1,703	-
3	238,381,040	both	487	+	339	-
4	233,798,159	both	1,022	+	1,239	-
5	230,387,390	both	1,504	+	1,136	-
6	229,045,783	both	798	+	992	-
7	226,392,888	both	1,109	+	465	-
8	218,682,584	both	758	+	1,561	-
9	211,879,600	both	494	+	502	-
10	198,488,806	both	839	+	494	-
11	336,010,529	both	620	+	1,559	-
12	333,808,457	both	2,861	+	1,221	-
13	303,725,412	right	0		1216	-
14	303,193,782	right	0		1349	-
15	296,304,349	both	552	+	567	-
16	283,435,706	both	431	-	2,326	-

Table S5 Centromere region in *B. chinensis* genome.

Chromosome	Chromosome length (bp)	Start	End	Centromere length (bp)
1	296,082,745	38,875,324	45,620,106	6,744,782
2	239,037,593	29,900,541	33,250,003	3,349,462
3	238,381,040	130,042,729	135,449,005	5,406,276
4	233,798,159	99,851,471	104,835,183	4,983,712
5	230,387,390	135,673,846	141,565,279	5,891,433
6	229,045,783	105,986,480	111,118,046	5,131,566
7	226,392,888	112,492,967	120,048,304	7,555,337
8	218,682,584	130,155,818	133,199,901	3,044,083
9	211,879,600	103,123,578	109,511,033	6,387,455
10	198,488,806	121,478,953	127,004,879	5,525,926
11	336,010,529	164,961,624	170,654,854	5,693,230
12	333,808,457	124,128,238	126,875,844	2,747,606
13	303,725,412	91,999,934	96,426,171	4,426,237
14	303,193,782	119,839,223	123,780,152	3,940,929
15	296,304,349	205,366,543	210,267,498	4,900,955
16	283,435,706	38,853,132	40,994,609	2,141,477

Table S6 Statistics of repeated sequence classification.

Type		Number of elements	Length(bp)	Percentage of sequence%
LTR		4,221,647	3,224,298,406	77.16
	Copia	285,773	227,777,016	5.45
	Gypsy	1,353,491	1,348,715,373	32.28
	unknown	2,582,383	1,647,806,017	39.43
LINE		3,972	1,390,720	0.03
DNA		1,678,407	431,053,262	10.32
Unclassified		601,698	113,356,996	2.71
Total			3,770,099,384	90.22

Table S7 Summary of protein-coding gene.

Stat	Total	Average	Mode	Min	1%	5%	10%	25%	Median	75%	90%	95%	99%	Max
Number of genes	33,962	NA	NA	NA	NA	NA	NA	NA	NA	NA	NA	NA	NA	NA
Number of genes (coding)	33,962	NA	NA	NA	NA	NA	NA	NA	NA	NA	NA	NA	NA	NA
Number of monoexonic genes	7,244	NA	NA	NA	NA	NA	NA	NA	NA	NA	NA	NA	NA	NA
Transcripts per gene	33,962	1	1	1	1	1	1	1	1	1	1	1	1	1
Coding transcripts per gene	33,962	1	1	1	1	1	1	1	1	1	1	1	1	1
CDNA lengths	44,073,365	1,297.73	348	150	198	276	360	606	1,050	1,650	2,481	3,182	5,124	17,268
CDNA lengths (mRNAs)	44,073,365	1,297.73	348	150	198	276	360	606	1,050	1,650	2,481	3,182	5,124	17,268
CDS lengths	41,750,544	1,229.33	312	150	195	264	345	579	1,017	1,580	2,334	2,973	4,707	16,194
CDS lengths (mRNAs)	NA	1,229.33	312	150	195	264	345	579	1,017	1,580	2,334	2,973	4,707	16,194
CDS/cDNA ratio	NA	96.38	100	8	44	80	84	100	100	100	100	100	100	100
Monoexonic transcripts	7,244	878.01	312	150	177	225	273	399	642	1,189	1,794	2,253	3,089	4,354
MonoCDS transcripts	7,484	867.07	312	150	174	219	264	390	630	1,185	1,784	2,208	3,073	5,364
Exons per transcript	184,769	5.44	1	1	1	1	1	2	4	7	12	15	24	79
Exons per transcript (mRNAs)	2,658	5.44	1	1	1	1	1	2	4	7	12	15	24	79
Exon lengths	NA	238.53	72	1	18	46	59	84	132	246	514	801	1,752	7,959
Exon lengths (mRNAs)	NA	238.53	72	1	18	46	59	84	132	246	514	801	1,752	7,959
Intron lengths	NA	2,007.42	86	21	68	77	83	100	306	1,808	5,167	8,982	20,742	495,249
Intron lengths (mRNAs)	NA	2,007.42	86	21	68	77	83	100	306	1,808	5,167	8,982	20,742	495,249

CDS exons per transcript	2,488	5.39	1	1	1	1	1	2	4	7	12	15	24	79
CDS exons per transcript (mRNAs)	2,488	5.39	1	1	1	1	1	2	4	7	12	15	24	79
CDS exon lengths	41,750,544	227.95	72	1	15	45	58	82	129	233	477	759	1,697	7,959
CDS Intron lengths	292,794,706	1,962.54	85	20	67	76	82	99	299	1,783	5,098	8,846	20,192	495,248
5'UTR exon number	33,962	0.16	0	0	0	0	0	0	0	0	1	1	2	5
3'UTR exon number	33,962	0.17	0	0	0	0	0	0	0	0	1	1	2	6
5'UTR length	695,250	20.47	0	0	0	0	0	0	0	0	53	152	353	3,460
3'UTR length	1,627,571	47.92	0	0	0	0	0	0	0	0	210	350	650	3,406
Stop distance from junction	NA	2.63	0	0	0	0	0	0	0	0	0	0	47	2,174
Intergenic distances	NA	112,514.46	25	-432,008	40	1,088	1,861	4,998	23,976	108,813	293,633	500,774	1,194,242	12,888,761
Intergenic distances (coding)	NA	112,514.46	25	-432,008	40	1,088	1,861	4,998	23,976	108,813	293,633	500,774	1,194,242	12,888,761

Table S8 Non-coding RNAs predicted in the *B. chinensis* genome.

Non-coding RNAs	Number
tRNA	778
rRNA	4,968
snRNA	478
miRNA	235
Total	6,459

Table S9 Multiple gene function databases annotate *B. chinensis* protein-coding genes.

Annotation database	Number of annotation	Annotation percentage (%)
Nr	32,752	96.44
KEGG	32,371	95.32
KOG	30,656	90.27
TrEMBL	32,465	95.59
SwissProt	26,099	76.85
Pfam	27,888	82.12
InterProscan	32,694	96.27
KO	15,530	45.73
GO	25,813	76.01
All	33,291	98.02
Total	33,962	-

Table S10 BUSCO of *B. chinensis* genome annotation.

Description	Number	Percentage
Complete BUSCOs (C)	1,574	97.5%
Complete and single-copy BUSCOs (S)	1,329	82.3%
Complete and duplicated BUSCOs (D)	245	15.2%
Fragmented BUSCOs (F)	16	1.0%
Missing BUSCOs (M)	24	1.5%
Total BUSCO groups searched	1,614	100.0%

Table S11 Ratio of different gene replication types.

Types	NO. of gene pairs	percentage/%
WGD-pairs	16,120	62.35
TD-pairs	1,191	4.61
PD-pairs	518	2.00
TRD-pairs	5,756	22.26
DSD-pairs	2,271	8.78
Total	25,856	100.00

Table S12 BUSCO of the *B. chinensis* genome assembly.

Description	Number	Percentage
Complete BUSCOs (C)	1,593	98.70%
Complete and single-copy BUSCOs (S)	1,331	82.47%
Complete and duplicated BUSCOs (D)	262	16.23%
Fragmented BUSCOs (F)	5	0.31%
Missing BUSCOs (M)	16	0.99%
Total BUSCO groups searched	1,614	100.00%

Table S13 Summary of CRAQ results for the *B. chinensis* genome.

Chromosome	Covered.Rate	Low-confident.Rate	Avg.CRH	Avg.CSH	Avg.CRE(R-AQI)	Avg.CSE(S-AQI)
Genome	0.997826153	0	4.045318922	0	0.00861521264101344(99.9138849738793)	0(100)
1	0.997972644	0	5.629510718	0	0.0101528861531629(99.8985226615786)	0(100)
2	0.999207719	0	4.492347855	0	0.0125602783458164(99.874476063823)	0(100)
3	0.999962199	0	5.312636783	0	0.00839024608554013(99.9161327274173)	0(100)
4	0.996930309	0	4.293015962	0	0.00858072739538331(99.9142295299599)	0(100)
5	0.998812105	0	1.460070285	0	0.00869135501683872(99.9131242087176)	0(100)
6	0.997502312	0	5.076816682	0	0.00875374277214402(99.9125008751076)	0(100)
7	0.9962028	0	2.871228855	0	0.013301809168867(99.8670703381613)	0(100)
8	0.993015539	0	1.981898682	0	0.0092100025453684(99.9079423736022)	0(100)
9	0.997207305	0	1.995220525	0	0.0141986369526238(99.8581143834286)	0(100)
10	0.99743052	0	3.06654271	0	0.0101020919942462(99.8990300890109)	0(100)
11	0.997645943	0	5.06054766	0	0.00596623926547146(99.9403554018117)	0(100)
12	0.998154891	0	4.470237599	0	0.00600253524279021(99.9399926591827)	0(100)
13	0.999104273	0	3.769694063	0	0.00659079868679259(99.9341137276749)	0(100)
14	0.997957577	0	5.46703078	0	0.00660994160202524(99.9339224248313)	0(100)
15	0.998396524	0	3.462542184	0	0.00676065706204006(99.9324162774723)	0(100)
16	0.998378574	0	4.557936652	0	0.00706773374651116(99.9293476330819)	0(100)

Table S14 The OrthoFinder analysis result of 16 (14+2) species.

Item	Value
Number of species	14+2
Number of genes	560,785
Number of genes in orthogroups	521,028
Number of unassigned genes	39,757
Percentage of genes in orthogroups	92.9
Percentage of unassigned genes	7.1
Number of orthogroups	35,037
Number of species-specific orthogroups	9,841
Number of genes in species-specific orthogroups	44,196
Percentage of genes in species-specific orthogroups	7.9
Mean orthogroup size	14.9
Median orthogroup size	5
G50 (assigned genes)	31
G50 (all genes)	28
O50 (assigned genes)	4,119
O50 (all genes)	4,792
Number of orthogroups with all species present	6,041
Number of single-copy orthogroups	30

Table S15 Summary of ortholog analysis of 16 (14+2) species.

	A. trichopoda	*A. shenzhenica*	*A. thaliana*	*A. officinalis*	*B. chinensis*	*C. sativus*	*D. nobile*	*G. max*	*M. truncatula*	*P. guangdongensis*	*P. zijinensis*	*P. montana*	*T. aestivumA*	*T. aestivumB*	*T. aestivumD*	*V. vinifera*
Number of genes	26,846	21,743	27,551	27,395	33,962	60,656	29,476	55,539	50,444	22,439	24,377	35,278	35,239	35,523	36,783	37,534
Number of genes in orthogroups	22,889	20,169	25,571	23,562	31,726	56,922	26,270	51,911	45,129	20,121	22,771	32,635	34,246	34,946	36,209	35,951
Number of unassigned genes	3,957	1,574	1,980	3,833	2,236	3,734	3,206	3,628	5,315	2,318	1,606	2,643	993	577	574	1,583
Percentage of genes in orthogroups	85.3	92.8	92.8	86	93.4	93.8	89.1	93.5	89.5	89.7	93.4	92.5	97.2	98.4	98.4	95.8
Percentage of unassigned genes	14.7	7.2	7.2	14	6.6	6.2	10.9	6.5	10.5	10.3	6.6	7.5	2.8	1.6	1.6	4.2
Number of orthogroups containing species	12,242	10,997	11,921	11,928	12,356	13,959	11,860	14,767	14,676	11,307	11,453	14,027	17,255	17,162	17,583	12,626
Percentage of orthogroups containing species	34.9	31.4	34	34	35.3	39.8	33.8	42.1	41.9	32.3	32.7	40	49.2	49	50.2	36
Number of species-specific orthogroups	859	393	719	722	362	1563	431	790	1502	432	369	507	234	160	144	654
Number of genes in species-specific orthogroups	4,674	2,026	3,633	3,576	1,222	5,126	1,813	2,709	9,127	1,363	1,233	1,427	843	567	481	4,376
Percentage of genes in species-specific orthogroups	17.4	9.3	13.2	13.1	3.6	8.5	6.2	4.9	18.1	6.1	5.1	4	2.4	1.6	1.3	11.7

Table S16 Members of BcOMTs in the *B. chinensis* genome.

Gene ID	Gene ID rename
Bch01G002312.t1	BcOMT01
Bch01G002313.t1	BcOMT02
Bch01G002314.t1	BcOMT03
Bch05G000094.t1	BcOMT04
Bch05G000095.t1	BcOMT05
Bch05G000096.t1	BcOMT06
Bch05G000097.t1	BcOMT07
Bch05G000100.t1	BcOMT08
Bch05G000101.t1	BcOMT09
Bch05G000104.t1	BcOMT10
Bch05G000105.t1	BcOMT11
Bch05G000106.t1	BcOMT12
Bch05G000107.t1	BcOMT13
Bch05G001540.t1	BcOMT14
Bch05G001543.t1	BcOMT15
Bch05G001546.t1	BcOMT16
Bch05G001548.t1	BcOMT17
Bch07G000124.t1	BcOMT18
Bch08G001014.t1	BcOMT19
Bch11G000930.t1	BcOMT20
Bch11G001300.t1	BcOMT21
Bch11G002693.t1	BcOMT22
Bch11G003020.t1	BcOMT23
Bch13G001116.t1	BcOMT24
Bch13G001117.t1	BcOMT25
Bch13G001118.t1	BcOMT26
Bch13G001119.t1	BcOMT27
Bch13G002491.t1	BcOMT28
Bch13G002492.t1	BcOMT29
Bch13G002493.t1	BcOMT30
Bch13G002495.t1	BcOMT31
Bch13G002496.t1	BcOMT32
Bch14G000039.t1	BcOMT33
Bch14G000041.t1	BcOMT34
Bch14G000042.t1	BcOMT35
Bch14G000043.t1	BcOMT36
Bch14G000045.t1	BcOMT37
Bch14G001553.t1	BcOMT38
Bch14G001554.t1	BcOMT39
Bch14G001555.t1	BcOMT40
Bch14G001556.t1	BcOMT41
Bch14G001558.t1	BcOMT42
Bch14G001559.t1	BcOMT43
Bch14G001561.t1	BcOMT44
Bch14G001562.t1	BcOMT45
Bch14G001563.t1	BcOMT46

Table S17 ^{13}C-NMR and ^{1}H-NMR data of compound **2** in MeOD.

No.	δ_C	δ_H（J in Hz）
2	153.4	8.06 (s, 1H)
3	126.8	
4	177.5	
5	142.5	
6	136.7	
7	155.2	
8	94.1	6.79 (s, 1H)
9	156.4	
10	114.2	
1'	128.9	
2'	111.9	6.62 (d, J = 1.9 Hz, 1H)
3'	152.4	
4'	138.1	
5'	154.4	
6'	105.6	6.66 (d, J = 2.0 Hz, 1H)
7'	104	6.12 (s, 2H)
5-OMe	61.3	4.06 (s, 3H)
3'-OMe	56.5	3.86 (s, 3H)
4'-OMe	61	3.81 (s, 3H)

^{13}C-NMR recorded at 200 MHz; ^{1}H-NMR recorded at 800 MHz.

Table S18 Functional plant flavonoid OMTs.

Name	species	Gene ID	site
EgCCoAOMT	*Eucalyptus gunnii*	O04854	
NtCCoAOMT	*Nicotiana tabacum*	O24151	
StCCoAOMT	*Solanum tuberosum*	Q8H9B6	
GmSOMT9	*Glycine max*	NP_001236240	3'
VvFAOMT	*Vitis vinifera*	ABQ02272	3'5'
ObFOMT2	*Ocimum basilicum*	AFU50295	7
ObFOMT2	*Ocimum basilicum*	AFU50296	7
MpOMT1B	*Mentha x piperita*	AAR09599	7
MpOMT1A	*Mentha x piperita*	AAR09598	7
MpOMT4	*Mentha x piperita*	AAR09602	4'
ObFOMT5	*Ocimum basilicum*	AFU50299	64'
ObFOMT3	*Ocimum basilicum*	AFU50297	64'
ObFOMT6	*Ocimum basilicum*	AFU50300	64'
ObFOMT4	*Ocimum basilicum*	AFU50298	6
OsNOMT1	*Oryza sativa*	BAM13734	7
MpOMT3	*Mentha x piperita*	AAR09601	3'
ShMOMT3	*Solanum habrochaites*	AGK26768	3'
ShMOMT1	*Solanum habrochaites*	ADZ76433	3'
CdFOMT5	*Citrus depressa*	BAU51794	3567
PlOMT4	*Pueraria lobata*	KP057887	3'
TaOMT2	*Triticum aestivum*	Q38J50	3'
CiOMT2	*Citrus reticulata*	ADK97702	73'
AtOMT1	*Arabidopsis thaliana*	U70424	3'
CapFOMT3'	*Chrysosplenium americanum*	Q42654	3'
CaOMT1	*Chrysosplenium americanum*	P59049	3'
CaOMT2	*Chrysosplenium americanum*	Q42653	3'
HvF1-OMT1	*Hordeum vulgare*	CAA54616	7
TaOMT6	*Triticum aestivum*	ON108663	2'5
CiCOMT10	*Chrysanthemum indicum*		4'
ObF8OMT-1	*Ocimum basilicum*	AGQ21572	8
MpOMT2	*Mentha x piperita*	AAR09600	8
CrOMT6	*Catharanthus roseus*	AAR02419	4'
CrOMT2	*Catharanthus roseus*	Q8GSN1	3'
AcOMT1	*Acorus calamus*	LC387636	4'
MtIOMT6	*Medicago truncatula*	ABD83945	74'
LjHI4'OMT	*Lotus japonicus*	Q84KK4	4'
MtIOMT5	*Medicago truncatula*	AAY18581	4'
MtIOMT7	*Medicago truncatula*	ABD83946	74'
GeHI4'OMT	*Glycyrrhiza echinata*	Q84KK6	4'
MtIOMT4	*Medicago truncatula*	DQ419912	7
GmSOMT2	*Glycine max*	C6TAY1	4'
GeD7OMT	*Glycyrrhiza echinata*	Q84KK5	7
MtIOMT2	*Medicago truncatula*	ABD83942	74'
MtIOMT3	*Medicago truncatula*	ABD83943	74'
MsI7OMT	*Medicago sativa L*	AAC49928	7
MtIOMT1	*Medicago truncatula*	AAY18582	7

Table S19 Ka and Ks values of BcOMTs paralogous gene pairs.

Gene ID1	Gene ID2	Ka_NG86	Ks_NG86
Bch01G002312.t1	Bch01G002313.t1	0.1881	0.8041
Bch01G002313.t1	Bch01G002314.t1	0.083	0.133
Bch14G000026.t1	Bch05G000094.t1	0.3859	3.0016
Bch14G000045.t1	Bch05G000094.t1	0.5178	1.6982
Bch05G000094.t1	Bch05G000095.t1	0.3574	1.2361
Bch05G000098.t1	Bch05G000094.t1	0.3012	2.9407
Bch05G000096.t1	Bch05G000094.t1	0.3781	1.461
Bch05G000094.t1	Bch05G000107.t1	0.3543	1.182
Bch14G000043.t1	Bch05G000095.t1	0.5624	1.3268
Bch05G000095.t1	Bch05G000096.t1	0.0336	0.1396
Bch05G000099.t1	Bch05G000095.t1	0.0316	0.0871
Bch05G000097.t1	Bch05G000095.t1	0.0399	0.1254
Bch05G000095.t1	Bch05G000106.t1	0.0812	0.1729
Bch05G000107.t1	Bch05G000095.t1	0.0048	0.0828
Bch05G000096.t1	Bch05G000097.t1	0.0537	0.1217
Bch05G000100.t1	Bch05G000096.t1	0.0243	0.0867
Bch05G000098.t1	Bch05G000096.t1	0.0339	0.0906
Bch05G000096.t1	Bch05G000105.t1	0.0428	0.1139
Bch05G000106.t1	Bch05G000096.t1	0.0908	0.1391
Bch05G000101.t1	Bch05G000097.t1	0.0444	0.1319
Bch05G000098.t1	Bch05G000097.t1	0.0479	0.1327
Bch05G000099.t1	Bch05G000097.t1	0.0672	0.0991
Bch05G000103.t1	Bch05G000097.t1	0.0645	0.2652
Bch05G000097.t1	Bch05G000107.t1	0.0405	0.1148
Bch14G000027.t1	Bch05G000100.t1	0.6641	1.9785
Bch14G000041.t1	Bch05G000100.t1	0.4833	1.1962
Bch05G000098.t1	Bch05G000100.t1	0.0253	0.0836
Bch05G000103.t1	Bch05G000100.t1	0.0646	0.1957
Bch05G000099.t1	Bch05G000100.t1	0.0394	0.0697
Bch05G000100.t1	Bch05G000101.t1	0.0368	0.0996
Bch05G000100.t1	Bch05G000105.t1	0.0201	0.0731
Bch14G000039.t1	Bch05G000101.t1	0.4749	1.1447
Bch05G000099.t1	Bch05G000101.t1	0.0447	0.0786
Bch05G000104.t1	Bch05G000101.t1	0.0358	0.1251
Bch05G000103.t1	Bch05G000101.t1	0.0724	0.1927
Bch14G000041.t1	Bch05G000104.t1	0.4908	1.2153
Bch14G000039.t1	Bch05G000104.t1	0.4705	1.2196
Bch05G000103.t1	Bch05G000104.t1	0.0424	0.2295
Bch05G000104.t1	Bch05G000105.t1	0.0386	0.1342
Bch05G000098.t1	Bch05G000104.t1	0.0356	0.1554
Bch14G000027.t1	Bch05G000105.t1	0.6497	1.9333
Bch05G000105.t1	Bch05G000106.t1	0.096	0.1495
Bch05G000106.t1	Bch05G000107.t1	0.0772	0.18
Bch05G000099.t1	Bch05G000106.t1	0.0913	0.1425
Bch14G000042.t1	Bch05G000107.t1	0.7513	1.796
Bch14G000026.t1	Bch05G000107.t1	0.4122	1.7305
Bch07G000124.t1	Bch05G000107.t1	0.148	0.5885
Bch05G001543.t1	Bch05G001540.t1	0.0357	0.035
Bch05G001548.t1	Bch05G001540.t1	0.3556	1.3298

Bch05G001543.t1	Bch05G001548.t1	0.3624	1.2905
Bch05G001547.t1	Bch05G001543.t1	0.3233	2.3329
Bch05G001546.t1	Bch05G001548.t1	0.167	0.2333
Bch11G003020.t1	Bch13G001119.t1	0.1599	0.736
Bch13G001116.t1	Bch11G003020.t1	0.1593	1.0745
Bch13G001116.t1	Bch13G001117.t1	0.027	0.1015
Bch13G001117.t1	Bch13G001118.t1	0.0049	0.0211
Bch13G001118.t1	Bch13G001119.t1	0.0049	0.0126
Bch13G002492.t1	Bch13G002491.t1	0.0186	0.042
Bch13G002496.t1	Bch13G002491.t1	0.0775	0.1846
Bch13G002493.t1	Bch13G002492.t1	0.0253	0.0659
Bch13G002492.t1	Bch13G002496.t1	0.1829	0.2903
Bch13G002495.t1	Bch13G002492.t1	0.0275	0.05
Bch13G002495.t1	Bch13G002493.t1	0.0077	0.0056
Bch13G002494.t1	Bch13G002493.t1	0.0255	0.0612
Bch13G002496.t1	Bch13G002495.t1	0.0722	0.1736
Bch14G000041.t1	Bch14G000039.t1	0.0485	0.1666
Bch14G000027.t1	Bch14G000039.t1	0.3503	0.5828
Bch14G000042.t1	Bch14G000041.t1	0.3721	0.5512
Bch14G000041.t1	Bch14G000043.t1	0.0879	0.2428
Bch14G000041.t1	Bch14G000045.t1	0.1194	0.1899
Bch14G000044.t1	Bch14G000041.t1	0.0727	0.1806
Bch14G000043.t1	Bch14G000042.t1	0.2434	0.4162
Bch14G000042.t1	Bch14G000044.t1	0.1917	0.5685
Bch14G000044.t1	Bch14G000043.t1	0.1987	0.2873
Bch14G000043.t1	Bch14G000045.t1	0.1318	0.1676
Bch14G000045.t1	Bch14G000044.t1	0.0197	0
Bch14G000045.t1	Bch14G000039.t1	0.1153	0.1779
Bch14G001554.t1	Bch14G001553.t1	0.2641	0.7838
Bch14G001553.t1	Bch14G001555.t1	0.0396	0.0934
Bch14G001553.t1	Bch14G001562.t1	0.0744	0.1203
Bch14G001556.t1	Bch14G001554.t1	0	0
Bch14G001554.t1	Bch14G001563.t1	0.102	0.1636
Bch14G001554.t1	Bch14G001561.t1	0.0196	0.0426
Bch14G001558.t1	Bch14G001555.t1	0	0
Bch14G001555.t1	Bch14G001562.t1	0.1101	0.1778
Bch14G001555.t1	Bch14G001560.t1	0.108	0.1773
Bch14G001559.t1	Bch14G001556.t1	0.0025	0.0119
Bch14G001556.t1	Bch14G001561.t1	0.0196	0.0426
Bch14G001563.t1	Bch14G001556.t1	0.102	0.1636
Bch14G001558.t1	Bch14G001561.t1	0.2956	0.6352
Bch14G001558.t1	Bch14G001559.t1	0.2877	0.659
Bch14G001557.t1	Bch14G001558.t1	0.1051	0.1768
Bch14G001561.t1	Bch14G001559.t1	0.017	0.0302
Bch14G001559.t1	Bch14G001563.t1	0.1019	0.1587
Bch14G001563.t1	Bch14G001561.t1	0.0932	0.1272
Bch14G001562.t1	Bch14G001560.t1	0.0584	0.071
Bch14G001562.t1	Bch14G001557.t1	0.0554	0.0708
Average			0.4708

Table S20 Members of BcUGTs in the *B. chinensis* genome.

Gene ID	Gene ID rename	Gene ID	Gene ID rename	Gene ID	Gene ID rename	Gene ID	Gene ID rename
Bch01G000974.t1	BcUGT001	Bch08G000207.t1	BcUGT038	Bch11G001673.t1	BcUGT075	Bch14G000120.t1	BcUGT112
Bch01G001377.t1	BcUGT002	Bch08G000210.t1	BcUGT039	Bch11G001730.t1	BcUGT076	Bch14G000121.t1	BcUGT113
Bch01G001946.t1	BcUGT003	Bch08G000211.t1	BcUGT040	Bch11G001800.t1	BcUGT077	Bch14G000704.t1	BcUGT114
Bch02G000627.t1	BcUGT004	Bch08G000212.t1	BcUGT041	Bch11G001808.t1	BcUGT078	Bch14G001654.t1	BcUGT115
Bch02G000629.t1	BcUGT005	Bch08G000213.t1	BcUGT042	Bch11G001894.t1	BcUGT079	Bch14G001655.t1	BcUGT116
Bch02G000861.t1	BcUGT006	Bch08G000214.t1	BcUGT043	Bch11G002875.t1	BcUGT080	Bch14G001659.t1	BcUGT117
Bch02G001325.t1	BcUGT007	Bch08G000215.t1	BcUGT044	Bch11G002876.t1	BcUGT081	Bch14G001998.t1	BcUGT118
Bch02G001327.t1	BcUGT008	Bch08G000220.t1	BcUGT045	Bch11G002877.t1	BcUGT082	Bch14G001999.t1	BcUGT119
Bch02G001587.t1	BcUGT009	Bch08G000441.t1	BcUGT046	Bch11G002879.t1	BcUGT083	Bch14G002003.t1	BcUGT120
Bch02G001589.t1	BcUGT010	Bch08G000442.t1	BcUGT047	Bch12G000074.t1	BcUGT084	Bch14G002007.t1	BcUGT121
Bch02G001616.t1	BcUGT011	Bch08G000443.t1	BcUGT048	Bch12G001200.t1	BcUGT085	Bch14G002008.t1	BcUGT122
Bch02G001869.t1	BcUGT012	Bch08G000444.t1	BcUGT049	Bch12G001201.t1	BcUGT086	Bch14G002009.t1	BcUGT123
Bch03G000928.t1	BcUGT013	Bch08G000811.t1	BcUGT050	Bch12G001634.t1	BcUGT087	Bch14G002011.t1	BcUGT124
Bch03G001093.t1	BcUGT014	Bch09G000935.t1	BcUGT051	Bch12G002006.t1	BcUGT088	Bch14G002013.t1	BcUGT125
Bch03G001094.t1	BcUGT015	Bch10G000046.t1	BcUGT052	Bch12G002072.t1	BcUGT089	Bch14G002175.t1	BcUGT126
Bch04G000627.t1	BcUGT016	Bch10G000282.t1	BcUGT053	Bch12G002093.t1	BcUGT090	Bch14G002176.t1	BcUGT127
Bch04G000714.t1	BcUGT017	Bch10G000310.t1	BcUGT054	Bch12G002198.t1	BcUGT091	Bch14G002380.t1	BcUGT128
Bch04G000946.t1	BcUGT018	Bch10G000311.t1	BcUGT055	Bch12G002827.t1	BcUGT092	Bch14G002429.t1	BcUGT129
Bch04G001360.t1	BcUGT019	Bch10G000513.t1	BcUGT056	Bch12G002828.t1	BcUGT093	Bch14G002430.t1	BcUGT130
Bch04G001362.t1	BcUGT020	Bch10G000514.t1	BcUGT057	Bch12G002829.t1	BcUGT094	Bch15G000382.t1	BcUGT131
Bch04G001558.t1	BcUGT021	Bch10G000737.t1	BcUGT058	Bch12G002830.t1	BcUGT095	Bch15G000588.t1	BcUGT132
Bch05G000225.t1	BcUGT022	Bch10G000738.t1	BcUGT059	Bch12G002831.t1	BcUGT096	Bch15G001411.t1	BcUGT133
Bch05G000226.t1	BcUGT023	Bch10G000739.t1	BcUGT060	Bch12G002832.t1	BcUGT097	Bch15G001524.t1	BcUGT134
Bch05G000373.t1	BcUGT024	Bch10G000885.t1	BcUGT061	Bch12G002833.t1	BcUGT098	Bch15G001984.t1	BcUGT135
Bch05G000869.t1	BcUGT025	Bch10G000992.t1	BcUGT062	Bch12G002834.t1	BcUGT099	Bch15G001985.t1	BcUGT136
Bch05G000870.t1	BcUGT026	Bch10G000996.t1	BcUGT063	Bch12G002835.t1	BcUGT100	Bch16G000078.t1	BcUGT137
Bch05G001523.t1	BcUGT027	Bch10G001210.t1	BcUGT064	Bch13G000123.t1	BcUGT101	Bch16G000440.t1	BcUGT138

Bch05G001524.t1	BcUGT028	Bch10G001614.t1	BcUGT065	Bch13G000125.t1	BcUGT102	Bch16G000645.t1	BcUGT139
Bch05G001630.t1	BcUGT029	Bch10G001615.t1	BcUGT066	Bch13G000246.t1	BcUGT103	Bch16G000646.t1	BcUGT140
Bch06G000189.t1	BcUGT030	Bch11G000200.t1	BcUGT067	Bch13G000925.t1	BcUGT104	Bch16G000687.t1	BcUGT141
Bch06G000224.t1	BcUGT031	Bch11G000492.t1	BcUGT068	Bch13G002041.t1	BcUGT105	Bch16G000688.t1	BcUGT142
Bch06G000760.t1	BcUGT032	Bch11G000529.t1	BcUGT069	Bch13G002438.t1	BcUGT106	Bch16G000950.t1	BcUGT143
Bch06G000954.t1	BcUGT033	Bch11G000782.t1	BcUGT070	Bch14G000031.t1	BcUGT107	Bch16G000960.t1	BcUGT144
Bch07G001181.t1	BcUGT034	Bch11G001239.t1	BcUGT071	Bch14G000032.t1	BcUGT108	Bch16G002042.t1	BcUGT145
Bch08G000043.t1	BcUGT035	Bch11G001292.t1	BcUGT072	Bch14G000117.t1	BcUGT109		
Bch08G000203.t1	BcUGT036	Bch11G001303.t1	BcUGT073	Bch14G000118.t1	BcUGT110		
Bch08G000204.t1	BcUGT037	Bch11G001351.t1	BcUGT074	Bch14G000119.t1	BcUGT111		

Table S21 CAZy annotation of BcUGTs by dbCAN3.

Target Name	Subfam Name	Subfam Composition	Substrate	HMM Length	Target Length	i-Evalue	HMM From	HMM To	Target From	Target To	Coverage	HMM File Name
BcUGT001	GT1_e389	GT1:11	unknown	420	479	4.86E-126	3	418	32	444	0.9904762	dbCAN-sub
BcUGT002	GT1_e197	GT1:213	-	375	456	1.58E-123	59	374	125	434	0.8426667	dbCAN-sub
BcUGT003	GT1_e124	GT1:180	unknown	414	467	1.07E-177	2	413	31	443	0.9951691	dbCAN-sub
BcUGT004	GT1_e623	GT1:15	-	399	478	7.68E-115	8	394	57	441	0.9699248	dbCAN-sub
BcUGT005	GT1_e389	GT1:11	unknown	420	477	2.88E-127	2	419	34	442	0.9952381	dbCAN-sub
BcUGT006	GT1_e19	GT1:239	-;-;-	448	497	3.46E-184	1	448	24	468	1	dbCAN-sub
BcUGT007	GT1_e640	GT1:90;GT0:2	-	339	488	4.72E-120	1	338	114	457	0.9970501	dbCAN-sub
BcUGT008	GT1_e640	GT1:90;GT0:2	-	339	462	2.17E-122	1	338	91	431	0.9970501	dbCAN-sub
BcUGT009	GT1_e298	GT1:17	-	413	490	4.43E-141	1	413	49	465	1	dbCAN-sub
BcUGT010	GT1_e253	GT1:50	-;-	360	475	3.06E-109	6	360	108	444	0.9861111	dbCAN-sub
BcUGT011	GT1_e525	GT1:45	unknown;-	425	463	1.91E-163	1	424	22	445	0.9976471	dbCAN-sub
BcUGT012	GT1_e176	GT1:773	unknown;-;-;-	427	496	1.38E-162	5	425	28	470	0.9859485	dbCAN-sub
BcUGT013	GT1_e157	GT1:55	-	379	471	1.41E-140	5	379	59	447	0.9894459	dbCAN-sub

BcUGT014	GT1_e197	GT1:213	-	375	456	4.73E-137	60	374	120	434	0.84	dbCAN-sub
BcUGT015	GT1_e424	GT1:43	-	175	138	3.74E-47	71	174	9	113	0.5942857	dbCAN-sub
BcUGT016	GT1_e389	GT1:11	unknown	420	502	2.76E-157	2	419	61	474	0.9952381	dbCAN-sub
BcUGT017	GT1_e19	GT1:239	-;-;-	448	483	5.78E-153	1	448	16	458	1	dbCAN-sub
BcUGT018	GT1_e18	GT1:362	unknown;-;-;-;-;-	421	478	5.30E-122	2	419	15	457	0.9928741	dbCAN-sub
BcUGT019	GT1_e18	GT1:362	unknown;-;-;-;-;-	421	470	2.36E-145	1	420	15	448	0.9976247	dbCAN-sub
BcUGT020	GT1_e56	GT1:261	-;-;-	371	471	6.39E-142	6	371	71	448	0.9865229	dbCAN-sub
BcUGT021	GT1_e176	GT1:773	unknown;-;-;-	427	497	2.24E-184	5	424	36	467	0.9836066	dbCAN-sub
BcUGT022	GT1_e352	GT1:35	unknown	308	468	2.88E-95	8	303	158	439	0.961039	dbCAN-sub
BcUGT023	GT1_e352	GT1:35	unknown	308	422	3.14E-78	39	303	164	393	0.8603896	dbCAN-sub
BcUGT024	GT1_e203	GT1:372	unknown;-;-;-;-;-	392	492	2.07E-154	6	392	77	462	0.9872449	dbCAN-sub
BcUGT025	GT1_e197	GT1:213	-	375	458	3.67E-138	59	374	122	437	0.8426667	dbCAN-sub
BcUGT026	GT1_e197	GT1:213	-	375	456	2.81E-140	38	374	99	435	0.8986667	dbCAN-sub
BcUGT027	GT1_e642	GT1:156	-;-;-	406	530	1.14E-161	1	405	35	455	0.9975369	dbCAN-sub
BcUGT028	GT1_e642	GT1:156	-;-;-	406	480	6.33E-161	1	405	35	454	0.9975369	dbCAN-sub
BcUGT029	GT1_e500	GT1:88	unknown	396	482	4.12E-135	2	396	62	447	0.9974747	dbCAN-sub

BcUGT030	GT1_e19	GT1:239	-;-;-	448	497	2.31E-179	1	447	18	472	0.9977679	dbCAN-sub
BcUGT031	GT1_e19	GT1:239	-;-;-	448	497	2.99E-182	1	448	21	466	1	dbCAN-sub
BcUGT032	GT1_e124	GT1:180	unknown	414	470	6.87E-177	2	412	31	444	0.9927536	dbCAN-sub
BcUGT033	GT1_e389	GT1:11	unknown	420	480	5.38E-127	3	419	36	442	0.9928571	dbCAN-sub
BcUGT034	GT1_e389	GT1:11	unknown	420	470	2.16E-133	1	420	27	427	1	dbCAN-sub
BcUGT035	GT1_e176	GT1:773	unknown;-;-;-	427	503	2.42E-182	5	425	33	472	0.9859485	dbCAN-sub
BcUGT036	GT1_e176	GT1:773	unknown;-;-;-	427	484	1.42E-162	5	424	29	461	0.9836066	dbCAN-sub
BcUGT037	GT1_e176	GT1:773	unknown;-;-;-	427	498	2.47E-169	6	425	34	469	0.9836066	dbCAN-sub
BcUGT038	GT1_e176	GT1:773	unknown;-;-;-	427	498	9.34E-170	6	425	34	469	0.9836066	dbCAN-sub
BcUGT039	GT1_e176	GT1:773	unknown;-;-;-	427	498	1.23E-168	6	425	34	469	0.9836066	dbCAN-sub
BcUGT040	GT1_e176	GT1:773	unknown;-;-;-	427	498	7.38E-170	6	425	34	469	0.9836066	dbCAN-sub
BcUGT041	GT1_e176	GT1:773	unknown;-;-;-	427	498	1.23E-168	6	425	34	469	0.9836066	dbCAN-sub
BcUGT042	GT1_e176	GT1:773	unknown;-;-;-	427	498	9.34E-170	6	425	34	469	0.9836066	dbCAN-sub
BcUGT043	GT1_e176	GT1:773	unknown;-;-;-	427	498	1.23E-168	6	425	34	469	0.9836066	dbCAN-sub
BcUGT044	GT1_e176	GT1:773	unknown;-;-;-	427	492	9.57E-172	6	425	34	469	0.9836066	dbCAN-sub
BcUGT045	GT1_e176	GT1:773	unknown;-;-;-	427	492	2.41E-171	6	425	34	469	0.9836066	dbCAN-sub

BcUGT046	GT1_e19	GT1:239	-;-;-	448	489	1.15E-178	1	448	19	461	1	dbCAN-sub
BcUGT047	GT1_e19	GT1:239	-;-;-	448	487	1.84E-183	1	448	19	463	1	dbCAN-sub
BcUGT048	GT1_e19	GT1:239	-;-;-	448	485	2.52E-184	1	448	19	461	1	dbCAN-sub
BcUGT049	GT1_e19	GT1:239	-;-;-	448	487	5.00E-183	1	448	19	463	1	dbCAN-sub
BcUGT050	GT1_e463	GT1:12	-	388	498	7.42E-163	1	387	81	469	0.9974227	dbCAN-sub
BcUGT051	GT1_e19	GT1:239	-;-;-	448	470	4.32E-118	2	448	21	445	0.9977679	dbCAN-sub
BcUGT052	GT1_e176	GT1:773	unknown;-;-;-	427	509	4.31E-184	5	425	45	483	0.9859485	dbCAN-sub
BcUGT053	GT1_e176	GT1:773	unknown;-;-;-	427	511	4.67E-178	5	425	39	488	0.9859485	dbCAN-sub
BcUGT054	GT1_e176	GT1:773	unknown;-;-;-	427	492	1.02E-153	5	425	28	464	0.9859485	dbCAN-sub
BcUGT055	GT1_e176	GT1:773	unknown;-;-;-	427	496	9.36E-154	5	424	34	473	0.9836066	dbCAN-sub
BcUGT056	GT1_e56	GT1:261	-;-;-	371	469	5.41E-145	7	371	71	447	0.9838275	dbCAN-sub
BcUGT057	GT1_e18	GT1:362	unknown;-;-;-;-;-	421	463	2.78E-145	1	420	12	444	0.9976247	dbCAN-sub
BcUGT058	GT1_e179	GT1:166	unknown	376	496	9.36E-150	14	375	87	461	0.962766	dbCAN-sub
BcUGT059	GT1_e179	GT1:166	unknown	376	498	2.56E-151	15	375	88	463	0.9601064	dbCAN-sub
BcUGT060	GT1_e179	GT1:166	unknown	376	501	8.46E-151	13	375	87	466	0.9654255	dbCAN-sub
BcUGT061	GT1_e626	GT1:62	-	388	478	5.74E-117	3	309	56	370	0.7912371	dbCAN-sub

BcUGT061	GT1_e401	GT1:186	unknown;-;-	294	478	1.45E-39	205	293	371	459	0.3027211	dbCAN-sub
BcUGT062	GT1_e626	GT1:62	-	388	463	1.32E-153	5	387	64	444	0.9871134	dbCAN-sub
BcUGT063	GT1_e626	GT1:62	-	388	462	7.22E-155	5	386	63	446	0.9845361	dbCAN-sub
BcUGT064	GT1_e176	GT1:773	unknown;-;-;-	427	489	2.71E-191	5	425	37	467	0.9859485	dbCAN-sub
BcUGT065	GT1_e537	GT1:44	unknown;-;unknown	383	465	4.36E-151	2	382	58	436	0.9947781	dbCAN-sub
BcUGT066	GT1_e537	GT1:44	unknown;-;unknown	383	473	9.23E-148	2	382	58	442	0.9947781	dbCAN-sub
BcUGT067	GT1_e499	GT1:22	-	377	502	2.79E-169	4	377	74	459	0.9920424	dbCAN-sub
BcUGT068	GT1_e623	GT1:15	-	399	548	6.13E-123	2	394	45	433	0.9849624	dbCAN-sub
BcUGT069	GT1_e203	GT1:372	unknown;-;-;-;-;-	392	490	4.94E-153	3	391	71	460	0.9923469	dbCAN-sub
BcUGT070	GT1_e266	GT1:22	-	407	535	3.59E-135	5	406	26	462	0.987715	dbCAN-sub
BcUGT071	GT1_e283	GT1:29	-	351	459	5.30E-134	5	350	87	434	0.985755	dbCAN-sub
BcUGT072	GT1_e203	GT1:372	unknown;-;-;-;-;-	392	466	1.17E-134	14	391	69	433	0.9642857	dbCAN-sub
BcUGT073	GT1_e18	GT1:362	unknown;-;-;-;-;-	421	483	1.27E-123	2	419	15	462	0.9928741	dbCAN-sub
BcUGT074	GT1_e19	GT1:239	-;-;-	448	482	5.72E-139	2	448	13	437	0.9977679	dbCAN-sub
BcUGT075	GT1_e463	GT1:12	-	388	503	1.64E-156	1	387	70	464	0.9974227	dbCAN-sub
BcUGT076	GT1_e642	GT1:156	-;-;-	406	470	1.60E-166	1	405	42	449	0.9975369	dbCAN-sub

BcUGT077	GT1_e176	GT1:773	unknown;-;-;-	427	475	3.93E-162	6	425	35	456	0.9836066	dbCAN-sub
BcUGT078	GT1_e516	GT1:92	-	273	484	9.81E-121	11	272	178	450	0.959707	dbCAN-sub
BcUGT079	GT1_e642	GT1:156	-;-;-	406	473	8.47E-148	1	404	44	451	0.9950739	dbCAN-sub
BcUGT080	GT1_e19	GT1:239	-;-;-	448	507	1.31E-150	1	448	19	475	1	dbCAN-sub
BcUGT081	GT1_e389	GT1:11	unknown	420	467	5.91E-135	1	420	5	421	1	dbCAN-sub
BcUGT082	GT1_e19	GT1:239	-;-;-	448	488	1.45E-154	1	448	17	455	1	dbCAN-sub
BcUGT083	GT1_e203	GT1:372	unknown;-;-;-;-;-	392	489	1.13E-145	3	392	65	462	0.994898	dbCAN-sub
BcUGT084	GT1_e642	GT1:156	-;-;-	406	469	3.84E-154	1	405	37	446	0.9975369	dbCAN-sub
BcUGT085	GT1_e513	GT1:142	unknown;-	385	455	2.12E-152	5	385	58	435	0.9896104	dbCAN-sub
BcUGT086	GT1_e445	GT1:96	-;-;unknown	283	494	2.41E-111	64	283	255	472	0.7773852	dbCAN-sub
BcUGT087	GT1_e78	GT1:50	-	395	478	1.84E-167	2	394	27	455	0.9949367	dbCAN-sub
BcUGT088	GT1_e108	GT1:27	-	319	468	2.01E-126	2	317	103	441	0.9905956	dbCAN-sub
BcUGT089	GT1_e623	GT1:15	-	399	468	1.64E-123	2	394	45	435	0.9849624	dbCAN-sub
BcUGT090	GT1_e253	GT1:50	-;-	360	487	1.94E-143	3	360	104	459	0.9944444	dbCAN-sub
BcUGT091	GT1_e499	GT1:22	-	377	467	5.15E-162	1	377	59	440	1	dbCAN-sub
BcUGT092	GT1_e528	GT1:9	-	378	467	2.73E-124	16	378	81	441	0.9603175	dbCAN-sub

BcUGT093	GT1_e528	GT1:9	-	378	463	6.67E-128	17	378	81	440	0.957672	dbCAN-sub
BcUGT094	GT1_e528	GT1:9	-	378	470	4.00E-118	16	378	81	441	0.9603175	dbCAN-sub
BcUGT095	GT1_e528	GT1:9	-	378	464	9.66E-122	17	378	82	441	0.957672	dbCAN-sub
BcUGT096	GT1_e528	GT1:9	-	378	482	4.51E-110	17	378	83	442	0.957672	dbCAN-sub
BcUGT097	GT1_e528	GT1:9	-	378	460	2.55E-120	12	378	73	437	0.9708995	dbCAN-sub
BcUGT098	GT1_e528	GT1:9	-	378	479	6.13E-109	17	378	83	439	0.957672	dbCAN-sub
BcUGT099	GT1_e528	GT1:9	-	378	467	1.12E-121	12	378	77	441	0.9708995	dbCAN-sub
BcUGT100	GT1_e528	GT1:9	-	378	486	2.55E-112	17	378	83	442	0.957672	dbCAN-sub
BcUGT101	GT1_e389	GT1:11	unknown	420	469	1.38E-108	4	419	41	438	0.9904762	dbCAN-sub
BcUGT102	GT1_e389	GT1:11	unknown	420	451	4.47E-109	4	408	41	442	0.9642857	dbCAN-sub
BcUGT103	GT1_e283	GT1:29	-	351	459	5.57E-163	2	350	84	435	0.994302	dbCAN-sub
BcUGT104	GT1_e19	GT1:239	-;-;-	448	490	4.15E-152	2	448	25	464	0.9977679	dbCAN-sub
BcUGT105	GT1_e500	GT1:88	unknown	396	497	1.06E-147	1	396	68	471	1	dbCAN-sub
BcUGT106	GT1_e626	GT1:62	-	388	479	1.07E-163	3	387	58	453	0.992268	dbCAN-sub
BcUGT107	GT1_e124	GT1:180	unknown	414	474	1.69E-169	3	413	34	448	0.9927536	dbCAN-sub
BcUGT108	GT1_e124	GT1:180	unknown	414	474	2.40E-168	3	413	34	448	0.9927536	dbCAN-sub

BcUGT109	GT1_e179	GT1:166	unknown	376	487	1.76E-165	1	375	62	451	0.9973404	dbCAN-sub
BcUGT110	GT1_e179	GT1:166	unknown	376	491	1.47E-160	1	376	64	461	1	dbCAN-sub
BcUGT111	GT1_e179	GT1:166	unknown	376	489	2.31E-161	1	374	64	462	0.9946809	dbCAN-sub
BcUGT112	GT1_e463	GT1:12	-	388	489	7.17E-161	1	388	65	465	1	dbCAN-sub
BcUGT113	GT1_e463	GT1:12	-	388	489	1.31E-161	1	388	65	465	1	dbCAN-sub
BcUGT114	GT1_e500	GT1:88	unknown	396	496	4.69E-159	2	396	63	463	0.9974747	dbCAN-sub
BcUGT115	GT1_e626	GT1:62	-	388	519	3.53E-145	4	387	70	473	0.9896907	dbCAN-sub
BcUGT116	GT1_e626	GT1:62	-	388	473	2.93E-147	4	387	68	455	0.9896907	dbCAN-sub
BcUGT117	GT1_e626	GT1:62	-	388	481	1.06E-137	3	387	58	456	0.992268	dbCAN-sub
BcUGT118	GT1_e18	GT1:362	unknown;-;-;-;-	421	468	2.33E-172	1	419	16	445	0.9952494	dbCAN-sub
BcUGT119	GT1_e18	GT1:362	unknown;-;-;-;-	421	477	7.85E-173	1	419	15	454	0.9952494	dbCAN-sub
BcUGT120	GT1_e18	GT1:362	unknown;-;-;-;-	421	475	3.91E-170	1	420	15	453	0.9976247	dbCAN-sub
BcUGT121	GT1_e18	GT1:362	unknown;-;-;-;-	421	478	1.26E-172	1	420	15	456	0.9976247	dbCAN-sub
BcUGT122	GT1_e18	GT1:362	unknown;-;-;-;-	421	477	2.57E-171	1	419	16	454	0.9952494	dbCAN-sub
BcUGT123	GT1_e18	GT1:362	unknown;-;-;-;-	421	472	2.86E-163	1	420	15	445	0.9976247	dbCAN-sub
BcUGT124	GT1_e18	GT1:362	unknown;-;-;-;-	421	472	2.06E-161	1	419	15	444	0.9952494	dbCAN-sub

BcUGT125	GT1_e18	GT1:362	unknown;-;-;-;-;-	421	475	5.36E-162	1	419	15	447	0.9952494	dbCAN-sub
BcUGT126	GT1_e19	GT1:239	-;-;-	448	487	4.22E-177	2	448	19	463	0.9977679	dbCAN-sub
BcUGT127	GT1_e19	GT1:239	-;-;-	448	487	4.90E-177	2	448	19	463	0.9977679	dbCAN-sub
BcUGT128	GT1_e176	GT1:773	unknown;-;-;-	427	500	1.69E-182	5	425	30	464	0.9859485	dbCAN-sub
BcUGT129	GT1_e176	GT1:773	unknown;-;-;-	427	486	9.07E-173	7	425	36	459	0.9812646	dbCAN-sub
BcUGT130	GT1_e176	GT1:773	unknown;-;-;-	427	489	4.80E-149	5	424	31	467	0.9836066	dbCAN-sub
BcUGT131	GT1_e19	GT1:239	-;-;-	448	508	7.68E-166	1	448	29	487	1	dbCAN-sub
BcUGT132	GT1_e19	GT1:239	-;-;-	448	502	5.76E-176	1	448	29	477	1	dbCAN-sub
BcUGT133	GT1_e386	GT1:8	-	382	481	1.74E-155	5	375	78	444	0.9712042	dbCAN-sub
BcUGT134	GT1_e124	GT1:180	unknown	414	529	2.53E-180	3	413	95	505	0.9927536	dbCAN-sub
BcUGT135	GT1_e640	GT1:90;GT0:2	-	339	475	1.89E-128	1	338	101	448	0.9970501	dbCAN-sub
BcUGT136	GT1_e640	GT1:90;GT0:2	-	339	472	5.00E-113	2	338	103	445	0.9941003	dbCAN-sub
BcUGT137	GT1_e359	GT1:70	unknown;unknown	344	298	2.32E-98	101	342	12	271	0.7034884	dbCAN-sub
BcUGT138	GT1_e253	GT1:50	-;-	360	476	2.51E-130	4	360	105	449	0.9916667	dbCAN-sub
BcUGT139	GT1_e499	GT1:22	-	377	478	1.72E-173	3	377	63	441	0.994695	dbCAN-sub
BcUGT140	GT1_e499	GT1:22	-	377	477	4.86E-169	3	377	63	441	0.994695	dbCAN-sub

BcUGT141	GT1_e176	GT1:773	unknown;-;-;-	427	454	8.92E-120	5	357	27	391	0.8266979	dbCAN-sub
BcUGT142	GT1_e176	GT1:773	unknown;-;-;-	427	480	6.72E-156	6	424	28	457	0.9812646	dbCAN-sub
BcUGT143	GT1_e19	GT1:239	-;-;-	448	486	1.69E-150	2	448	19	457	0.9977679	dbCAN-sub
BcUGT144	GT1_e389	GT1:11	unknown	420	511	6.22E-156	1	419	50	470	0.997619	dbCAN-sub
BcUGT145	GT1_e19	GT1:239	-;-;-	448	480	4.55E-139	3	448	17	455	0.9955357	dbCAN-sub

Table S22 Contents of 9 characteristic isoflavones in the six organs of *B. chinensis* (n=3).

ug/g	dichotomitin	irisflorentin	iristectorigenin A	tectorigenin	irigenin	iristectorin B	iristectorin A	tectoridin	iridin
Root1	47.30±0.42	571.44±9.03	38.40±0.93	-	380.80±7.21	-	124.00±2.27	-	1415.06±26.30
Root2	165.47±4.52	945.84±1.80	153.62±0.75	129.76±2.19	1174.73±7.38	115.30±0.44	186.48±0.13	-	2344.95±10.73
Root3	179.13±1.06	573.15±5.54	165.80±1.36	65.90±0.88	939.14±8.06	85.92±0.74	148.41±0.52	141.82±1.55	1523.34±14.08
Rhizome1	-	867.72±1.47	310.72±2.99	323.32±2.72	1179.17±2.02	992.96±1.59	1913.88±8.30	4238.57±10.02	5712.11±11.15
Rhizome2	39.82±0.70	302.60±1.24	94.66±0.37	158.41±0.92	345.05±1.54	1126.99±42.42	1632.33±6.74	8890.98±20.50	2842.41±9.10
Rhizome3	26.07±0.21	228.80±3.68	237.73±2.98	222.98±9.11	752.82±7.79	1145.94±8.49	4636.18±39.05	5660.60±171.29	4794.82±35.89
Aerial Stem 1	-	32.72±0.27	-	-	-	227.85±0.06	602.53±1.04	994.40±3.34	523.45±1.52
Aerial Stem 2	-	-	-	-	-	266.11±1.64	486.53±0.43	1311.60±6.21	608.38±3.26
Aerial Stem 3	-	-	-	-	-	209.64±3.24	357.17±3.01	741.28±9.35	581.93±4.75
Leaf 1	-	-	-	-	-	-	56.32±0.49	-	32.03±0.49
Leaf 2	-	-	-	-	-	-	93.91±0.76	-	25.36±0.24
Leaf 3	-	-	-	-	-	-	52.56±0.45	-	11.30±0.70
Flower 1	-	-	-	-	-	-	56.68±0.34	-	45.92±0.27
Flower 2	-	-	-	-	-	-	-	-	45.04±0.30
Flower 3	-	-	-	-	-	-	-	-	-
Fruit 1	-	-	-	-	-	-	77.62±0.36	-	52.50±0.30
Fruit 2	-	-	-	-	21.13±0.26	-	85.62±0.52	-	39.45±0.71
Fruit 3	-	-	-	-	21.94±0.29	-	104.82±0.51	-	70.80±0.24

Note: Metabolite profiling data for *B. chinensis* were obtained from a previously published study (Tian et al., 2018).

Table S23 Primers used to construct recombinant BcOMTs plasmids in this study.

Primer name	Primer sequences (5' to 3')
BcOMT01-F	agcaaatgggtcgcggatccATGGAATCTAGTCCATTGATAAAAATCCCT
BcOMT01-R	cggagctcgaattcggatccTTATTTGTAGAACTCTAGGATCCAGCAAT
BcOMT02-F	agcaaatgggtcgcggatccATGGGATCTACAGAGCAGAACG
BcOMT02-R	cggagctcgaattcggatccCTACTTGTAGAACTCGATCACCCAT
BcOMT03-F	agcaaatgggtcgcggatccATGGGATCTACAGAGCAGAAGG
BcOMT03-R	cggagctcgaattcggatccCTACTTGTAGAACTCGAGCACCC
BcOMT05-F	agcaaatgggtcgcggatccATGTACCTCCAAACAGCTGCTA
BcOMT05-R	cggagctcgaattcggatccTCAGTGATATATCTCGATGATCGAACG
BcOMT06-F	agcaaatgggtcgcggatccATGAGGAAGCCGGGAATGTAC
BcOMT06-R	cggagctcgaattcggatccTCAGTGATATATCTCGATGATTGAACGC
BcOMT07-F	agcaaatgggtcgcggatccATGAGGCCGAATAGGAGCAT
BcOMT07-R	cggagctcgaattcggatccTCAGTGATATATCTCGATGATCGAACG
BcOMT08-F	agcaaatgggtcgcggatccATGTACGTCCAAACAGCTGC
BcOMT08-R	cggagctcgaattcggatccTCAGTGATATATCTCGATGATCGAACG
BcOMT09-F	agcaaatgggtcgcggatccATGTTCGTCCAAACGCCTG
BcOMT09-R	cggagctcgaattcggatccTCAGTGATATATCTCGATGATCGAACG
BcOMT13-F	agcaaatgggtcgcggatccATGGCTTCAAGCACCACC
BcOMT13-R	cggagctcgaattcggatccTCAGTGATATATCTCGATGATCGA
BcOMT18-F	agcaaatgggtcgcggatccATGGCATCGATCGAAGGAC
BcOMT18-R	cggagctcgaattcggatccTTAAGAATATGATATCTCGATGATGGAACG
BcOMT33-F	agcaaatgggtcgcggatccATGCATACAAGTCGGAGCA
BcOMT33-R	cggagctcgaattcggatccTCAAGGATAAACTTCAATGATCGAA

Table S24 Primers used to construct recombinant BcUGTs plasmids in this study.

Primer names	Primer sequences (5' to 3')
BcUGT001-F	agcaaatgggtcgcggatccATGGCCGTCGTTGAACC
BcUGT001-R	cggagctcgaattcggatccCTAATTGGGACGAGCAGATTGTG
BcUGT003-F	agcaaatgggtcgcggatccATGGCACCCTTTATTTCTCCC
BcUGT003-R	cggagctcgaattcggatccTCAGGGCATCCCCTTCC
BcUGT004-F	agcaaatgggtcgcggatccATGGATTCTTCTTCTTCTTCTTCTTCC
BcUGT004-R	cggagctcgaattcggatccCTAGACTCCATTCCCCGAAGC
BcUGT005-F	agcaaatgggtcgcggatccATGGGTGGCATCGAACCT
BcUGT005-R	cggagctcgaattcggatccTCAAACCAAAGTAGTCTCTTTTCCAG
BcUGT009-F	agcaaatgggtcgcggatccATGGATCTATCTCCGATGGAGAATTC
BcUGT009-R	cggagctcgaattcggatccTTAGGCCGGTTCCAAGTTTTT
BcUGT011-F	agcaaatgggtcgcggatccATGCCGAGCTCCGGC
BcUGT011-R	cggagctcgaattcggatccCTAGGCCCCCAACTTGCG
BcUGT013-F	agcaaatgggtcgcggatccATGGAAGATGGAGACAAGCTTCA
BcUGT013-R	cggagctcgaattcggatccTCAGAGCGGGGAGTTTCG
BcUGT022-F	agcaaatgggtcgcggatccATGTCTTCCTCCCGCGAG
BcUGT022-R	cggagctcgaattcggatccCTATTTCCTGTGTCCGCTGACG
BcUGT023-F	agcaaatgggtcgcggatccATGAGCACCGCCGCC
BcUGT023-R	cggagctcgaattcggatccCTATTTCCTGTGTCCGCTGAC
BcUGT027-F	agcaaatgggtcgcggatccATGGAGCAAGTGACGGTAGT
BcUGT027-R	cggagctcgaattcggatccTCAATCAATTGATGCATCTCTGGTG
BcUGT032-F	agcaaatgggtcgcggatccATGGACCAAACACCTCACATAGT
BcUGT032-R	cggagctcgaattcggatccTCAAACTAGCAGCCCCTTC
BcUGT033-F	agcaaatgggtcgcggatccATGGGTGGTTGTCCAGAATCC
BcUGT033-R	cggagctcgaattcggatccCTAAGAAAGTACTGTTTGTTCACTCTTTCT
BcUGT035-F	agcaaatgggtcgcggatccATGGGTTCATTAATGGCGGC
BcUGT035-R	cggagctcgaattcggatccCTAGATCTTTTGCTGCTGCTGC
BcUGT044-F	agcaaatgggtcgcggatccATGGGTTCTATCGGAGAGAGG
BcUGT044-R	cggagctcgaattcggatccTCAAGCTTCTTGCCTAATAGAATCCA
BcUGT045-F	agcaaatgggtcgcggatccATGGGTTCTATAGGAGAGAGGAAGG
BcUGT045-R	cggagctcgaattcggatccTTAGAGACCATTCCCTCCAGAAAG
BcUGT053-F	agcaaatgggtcgcggatccATGGGTTGTTCTTCTCCTCCT
BcUGT053-R	cggagctcgaattcggatccTCAACTAGCCTTGCTTGGGA
BcUGT064-F	agcaaatgggtcgcggatccATGGGTTCAATAGGCGAAGC
BcUGT064-R	cggagctcgaattcggatccTCATTTTTTTGGGAGGAGCACC
BcUGT065-F	agcaaatgggtcgcggatccATGTCTTCATCCTGCCCTCA
BcUGT065-R	cggagctcgaattcggatccTCATCCATGAGGAGAAGCAGAAG
BcUGT076-F	agcaaatgggtcgcggatccATGACTCGATCACAAGAAGTAGCC
BcUGT076-R	cggagctcgaattcggatccTCATCGGGTGATACGAGCAA
BcUGT080-F	agcaaatgggtcgcggatccATGGCCTCGGAAAGCCG
BcUGT080-R	cggagctcgaattcggatccTTACCAGATATCATCACATGGTGGTAG
BcUGT081-F	agcaaatgggtcgcggatccATGGCCAAGCACTTCGC
BcUGT081-R	cggagctcgaattcggatccTTACCAGATATCATCACATGGTGGTAG
BcUGT084-F	agcaaatgggtcgcggatccATGGAGCAACAACAACAAGTATCAT
BcUGT084-R	cggagctcgaattcggatccTTATGACCACCTAGTAACATAATCCATGAA
BcUGT094-F	agcaaatgggtcgcggatccATGGCTACCGACAGCCC
BcUGT094-R	cggagctcgaattcggatccCTAACAAACATTAATTTCTCGAGCAGGC
BcUGT095-F	agcaaatgggtcgcggatccATGGCCAGCGACAGCC

BcUGT095-R	cggagctcgaattcggatccTCAAGCAGACCTATTGGATTTAAGATGC
BcUGT097-F	agcaaatgggtcgcggatccATGGCTACCGACAGCCC
BcUGT097-R	cggagctcgaattcggatccTTAATTTTCTCGAGCAGGCCTATTG
BcUGT103-F	agcaaatgggtcgcggatccATGTGTTCGATCGACCATCC
BcUGT103-R	cggagctcgaattcggatccTCAACGAGCAATCACAATCTCCAAAA
BcUGT104-F	agcaaatgggtcgcggatccATGGGCAAAGAAAACCAGTGT
BcUGT104-R	cggagctcgaattcggatccTCAAACGCATGCATTCTTCTTGT
BcUGT115-F	agcaaatgggtcgcggatccATGAAGCAGAATGTTGTTGACCAAC
BcUGT115-R	cggagctcgaattcggatccTTACGTGGCATACACTCCAATCAG
BcUGT118-F	agcaaatgggtcgcggatccATGTCCGCCAAGCTGACG
BcUGT118-R	cggagctcgaattcggatccTCAGATTCCACGTTTCATCCTCTC
BcUGT119-F	agcaaatgggtcgcggatccATGCAGCTCAAAGAATCAGTAGTACT
BcUGT119-R	cggagctcgaattcggatccTCAGGCTTCCTTCCTGACAG
BcUGT123-F	agcaaatgggtcgcggatccATGGAGTTCACGCAATCAGTC
BcUGT123-R	cggagctcgaattcggatccTCAAGCTCCGGTCCCC
BcUGT124-F	agcaaatgggtcgcggatccATGGAGTTCACGCAATCAGTC
BcUGT124-R	cggagctcgaattcggatccTCAAGCTCTGGTGTCCG
BcUGT130-F	agcaaatgggtcgcggatccATGGTGATGGTGGGGAGG
BcUGT130-R	cggagctcgaattcggatccCTAAAATTTCCCAGAACTCAAGTCCT
BcUGT135-F	agcaaatgggtcgcggatccATGAGTCCAAGCAGCATGATG
BcUGT135-R	cggagctcgaattcggatccTCATTTGACAGTCACATCCCCC

Table S25 Gradient elution conditions of O-methylation reaction.

time\min	0.1% v/v formic acid aqueous solution\%	acetonitrile\%
0	70	30
5	58	42
15	58	42
20	58	80
25	20	30

Table S26 Gradient elution conditions of O-glycosylation reaction.

time\min	0.1% v/v formic acid aqueous solution\%	acetonitrile\%
0	90	10
10	85	15
18	75	25
23	65	35
35	40	60
36	90	10
41	90	10

Table S27 Primers used for transient expression in *Nicotiana benthamiana*.

Primer names	Primer sequences (5' to 3')
PEAQ-BcOMT03F	ctgcccaaattcgcgaccggtATGGGATCTACAGAGCAGAAGG
PEAQ-BcOMT03R	accagagttaaaggcctcgagCTACTTGTAGAACTCGAGCACCC
PEAQ-BcOMT33F	ctgcccaaattcgcgaccggtATGCATACAAGTCGGAGCATTTCC
PEAQ-BcOMT33R	accagagttaaaggcctcgagTCAAGGATAAACTTCAATGATCGAACG
PEAQ-BcUGT009F	ctgcccaaattcgcgaccggtATGGATCTATCTCCGATGGAGAATTC
PEAQ-BcUGT009R	accagagttaaaggcctcgagTTAGGCCGGTTCCAAGTTTTTGAG
PEAQ-BcUGT032F	ctgcccaaattcgcgaccggtATGGACCAAACACCTCACATAGTC
PEAQ-BcUGT032R	accagagttaaaggcctcgagTCAAACTAGCAGCCCCTTCCA
PEAQ-BcUGT119F	ctgcccaaattcgcgaccggtATGCAGCTCAAAGAATCAGTAGTACT
PEAQ-BcUGT119R	accagagttaaaggcctcgagTCAGGCTTCCTTCCTGACAGC
PEAQ-BcUGT124F	ctgcccaaattcgcgaccggtATGGAGTTCACGCAATCAGTCA
PEAQ-BcUGT124R	accagagttaaaggcctcgagTCAAGCTCTGGTGTCCGGAGT

Table S28 Primers used for site-directed mutagenesis.

Primer names	Primer sequences (5' to 3')
BcUGT009N386A-F	TGGgcgTCGATCCTGGAGGCCACCGCCGCGGG
BcUGT009N386A-R	TCCAGGATCGAcgcCCACCCGCAGTGCGTCAC
BcUGT009H32A-F	CCAAGGCgcgCTCCTCCCCCTCCTCGACCTCG
BcUGT009H32A-R	GGAGGAGcgcGCCTTGGGCCGGGAATGGGATT
BcUGT009T308A-F	ATGTATGCTTTGGCgcgCAGGTGGTGCTTAGTCAAGCACA
BcUGT009T308A-R	cgcGCCAAAGCATACATACAACACCGATCCAC
BcUGT009K404A-F	ATGgcgGCGGACCAGTTCGTCAACGCGAGGCT
BcUGT009K404A-R	AACTGGTCCGCcgcCATCGGCCACATCAGCGT
BcUGT009D135A-F	TCATCTCCgccTTCTTCCTCGGCTGGACGAAC
BcUGT009D135A-R	GAAGAAggcGGAGATGATGGAGGTGGGGGAT
